# Haematological toxicities with immune checkpoint inhibitors in digestive system tumors: a systematic review and network meta-analysis of randomized controlled trials

**DOI:** 10.1007/s10238-025-01688-x

**Published:** 2025-05-13

**Authors:** Xinpu Han, Jing Xu, Meichen Cui, Zhangjun Yun, Hongbin Zhao, Shaodan Tian, Suicai Mi, Li Hou

**Affiliations:** 1 Department of Oncology and Hematology, Dongzhimen Hospital, Beijing University of Chinese Medicine, Beijing, China; 2 Beijing University of Chinese Medicine, Beijing, China; 3 Hubei Provincial Hospital of Traditional Chinese Medicine, Hubei, China; 4 Xiamen Hospital, Dongzhimen Hospital, Beijing University of Chinese Medicine, Xiamen, China

**Keywords:** Immune checkpoint inhibitors, Haematological toxicities, Digestive system tumors, Systematic review, Network meta-analysis

## Abstract

This study aims to comprehensively evaluate the hematologic toxicity profiles, toxicity spectrum, and safety rankings of immune checkpoint inhibitors (ICIs) used for digestive system tumors. The PubMed, Cochrane Library, Web of Science, and Embase databases were systematically searched from inception to August 2024 to identify randomized controlled trials (RCTs). The primary outcome was anemia, while secondary outcomes included neutropenia, neutrophil count decreased, thrombocytopenia, platelet count decreased, leukopenia, white blood cell (WBC) count decreased, lymphocyte count decreased, and febrile neutropenia (FN). Subgroup analyses were performed based on tumor type, country category, study phase, ICI regimen, control group, chemotherapy regimen, ICI plus different chemotherapy regimens. Two reviewers independently selected the studies, extracted data according to pre-specified criteria, and assessed the risk of bias using the Cochrane Collaboration risk of bias tool. RevMan 5.4 software was utilized to visualize the risk of bias assessments. Stata 16.0 was used to conduct network meta-analysis, sensitivity analysis and meta-regression. 25 phase II and III RCTs (*n* = 15216) were included. The general safety of ICIs ranked from high to low for grade 1–5 anemia were as follows: avelumab, nivolumab, pembrolizumab, sintilimab, camrelizumab, and tislelizumab. For grade 3–5 anemia, the general safety profile of the ICIs were as follows, from highest to lowest: avelumab, nivolumab, pembrolizumab, sintilimab, and camrelizumab. Compared to chemotherapy, treatment-related hematologic toxicities with ICIs occurred primarily in grade 1–5 anemia, neutropenia, thrombocytopenia, leukopenia, and WBC count decreased. Taking ICI monotherapy, nivolumab plus ipilimumab were generally safer than taking chemotherapy, one ICI drug with chemotherapy, or two ICI drugs with chemotherapy. In terms of grade 1–5 hematologic toxicities, tislelizumab had the highest risk of neutropenia and leukopenia; the primary treatment-adverse events (AEs) for sintilimab was neutrophil count decreased and WBC count decreased; the primary treatment-related AE associated with nivolumab was platelet count decreased; camrelizumab posed the highest risk for lymphocyte count decreased. In terms of grade 3–5 hematologic toxicities, pembrolizumab was predominantly linked to neutropenia; sintilimab showed the greatest risk for neutrophil count decreased, platelet count decreased, and lymphocyte count decreased; avelumab was most associated with WBC count decreased. FN primarily manifested as grade 3–5, with camrelizumab having the highest risk. Among agents used in gastric or gastroesophageal junction cancer, avelumab demonstrated the most favorable safety profile for anemia. Each treatment regimen has its unique safety profile. Early identification and management of ICI-related hematologic toxicities are essential in clinical practice.

*Systematic Review Registration*: PROSPERO CRD42024571508.

**Supplementary Information:**

The online version contains supplementary material available at 10.1007/s10238-025-01688-x.

## Introduction

Digestive system tumors are malignant tumors that occur in the digestive tract and its related organs. The common feature of these tumors is that they originate from epithelial cells or other digestive system tissues and exhibit uncontrolled growth and metastatic ability [1]. According to the epidemiology of digestive diseases in 2023, digestive system tumors account for approximately 22% of newly diagnosed cancers as well as 37% of cancer deaths globally [2]. The use of immune checkpoint inhibitors (ICIs) and combination therapy for digestive system malignancies has grown in recent years. While ICIs have shown significant therapeutic effects, they also cause immune-related adverse events (irAEs) involving dermal, hepatic, and hematologic [3]. These irAEs are distinct in organ involvement, pathogenesis, and severity compared to those from conventional treatments like chemotherapy, radiotherapy, and targeted therapies [4].

Hematologic toxicities of ICIs refer to their AEs on blood components and bone marrow. In digestive system tumor patients, these toxicities are rare but can cause serious health issues, such as increased infection and bleeding risks. This can delay or interrupt treatment, impair quality of life, and may even be fatal [5]. Studies estimated the prevalence of ICI-related hematologic toxicities at 3.6% [6]. While hematologic toxicities are common in chemotherapy, oncologists have developed an evidence-based framework for managing chemotherapy-related hematologic issues [7, 8]. In contrast, ICI-related hematologic toxicities are difficult to diagnose, resulting in limited understanding [9]. High-quality research is urgently needed to improve early identification, management, and treatment of these toxicities.

Most clinical trials have reported hematologic toxicities associated with ICIs in digestive system tumors, offering valuable insights into these rare but clinically significant issues. However, a comprehensive understanding of irAEs from randomized controlled trials (RCTs) was hindered by study design limitations and practical constraints [10]. Previous meta-analyses on this topic have primarily focused on specific irAEs or particular ICI drugs, failing to provide an all-encompassing comparison [6, 11]. Additionally, the relevant meta-analysis was limited to phase III RCTs, affecting the reliability and generalizability of their findings [11]. In this study, we included both Phase II and Phase III RCTs and conducted a Bayesian network meta-analysis (NMA). We evaluated the hematologic safety of ICI monotherapies and combinations, examining both grade 1–5 and 3–5 hematologic toxicities. We also assessed the risk of hematologic toxicity across different tumor types and ICI drugs.

## Methods

This NMA was reported under the preferred reporting items for systematic reviews and meta-analysis (PRISMA) and the PRISMA extended statement for NMA (Table S1) [12, 13]. The registration number for this study was CRD42024571508.

### Search strategy and selection criteria

We searched Embase, Web of Science, PubMed, and Cochrane Library databases to include the period from the start of the databases through August 2024. Searches were conducted using search terms combined with digestive system tumors, ICIs, and hematologic toxicities. The search strategy was described in detail in Table S2. The following were the studies’ inclusion criteria: (1) used an RCT design, with full-text accessibility and results made available to the public; (2) included patients aged ≥ 18 years with digestive system tumor diagnoses; (3) compared two or more treatment modalities, including at least one ICI; (4) reported primary outcomes, including the incidence of anemia. Complete reporting of baseline characteristics and outcome indices was required. The following were the exclusion criteria: (1) failed to provide a clear description of the ICIs used in the methods; (2) reviews, meta-analyses, conference abstracts, letters, case reports, experimental studies, or non-RCTs, as these brief reports lack detailed data; and (3) did not assess hematologic toxicity-related outcomes associated with ICIs.

### Study screening and data extraction

All the retrieved studies were imported into Endnote V.X9. Duplicated studies were eliminated. Endnote V.X9 was used to import all of the recovered studies. Duplicate research was removed. Two reviewers (XPH and JX) initially screened all of the study titles and abstracts according to the inclusion and exclusion criteria. Two reviewers (XPH and JX) independently extracted data from all included studies. A third senior researcher (MCC) was consulted to resolve any disagreements. A structured data extraction form that was standardized was employed. The following information was taken from studies that qualified: general study characteristics (e.g., the first author’s name, year of publication, country of study, trial phase, clinical registration number), participant characteristics (e.g., participant gender, cancer type, age, and sample size), details of the intervention (e.g., drug name, dosage, and frequency of administration), and outcomes (including baseline and endpoint metrics to minimize baseline data bias across studies). To acquire complete datasets for any missing information, the corresponding authors were contacted by phone or email. The PRISMA guidelines were adhered to during the screening procedure.

### Quality assessment

The risk of bias (ROB) in RCTs and the methodological quality of the included studies were evaluated using the Cochrane System Evaluation Manual (Version 5.1.0). The evaluations were conducted independently by two reviewers (XPH and JX). Randomization methods, blinding, selective reporting, allocation concealment, and incomplete outcome data were all classified as having a low, high, or uncertain ROB [14]. RevMan 5.4 software was utilized to visualize the ROB assessments. If two or more domains were evaluated as high risk, the study was considered to have a high ROB. On the other hand, if five or more domains were assessed as low risk and none as high risk, the study was considered to have a low ROB. The ROB was deemed to be modest for all other research.

### Statistical analysis

Effect sizes were computed and compared using Stata software (version 16.0). The network geometry was shown by visualizing the evidence network for all findings [15]. The particular cycle and node-splitting method were used to assess the presence of inconsistency between global and local results [16], with *P* < 0.05 indicating significant inconsistency. We used a conservative approach to handling study heterogeneity to identify the best model for the NMA. We utilized a fixed-effects model for the meta-analysis if the study findings showed no heterogeneity (I^2^ ≤ 50%). We employed a random effects model if the study results showed heterogeneity (I^2^ > 50%) [17]. We extracted the number of patients who developed primary endpoint and secondary endpoints, as well as the total number of patients treated with the study drug for which toxicity could be assessed to calculate the grade 1–5 and 3–5 hematologic toxicities incidence. The weight of each study in the analysis was based on the sample size of individuals. The combined effect size ratio (odds ratio, OR) of different treatments in terms of hematologic toxicities was calculated in a Bayesian framework. Subgroup analyses were conducted based on the cancer type. The surface under the cumulative probability ranking curve (SUCRA) was used to determine the probability that each treatment was ranked as the safest. The safer the treatment, the higher the SUCRA rating, which varied from 0 to 100% [18]. Funnel plots for evaluating small-study effects and publication bias in ICI hematologic toxicities.

### Subgroup analysis

Subgroup analysis was performed according to: (1) tumor type: gastric or gastro-oesophageal junction cancer, esophageal cancer; (2) country category: MN, China; (3) study phase: phase II, phase III; (4) ICI regimen: ICI plus chemotherapy, ICI; (5) control group: chemotherapy with placebo, chemotherapy without placebo; (6) chemotherapy regimen: taxane-based, platinum-based, taxane-based plus irinotecan, platinum-based plus 5-fluorouracil, taxane-based plus platinum-based; (7) ICI plus different chemotherapy regimens: ICI plus platinum-based, ICI plus platinum-based and 5-fluorouracil, ICI plus taxane-based and platinum-based.

### Additional analyses (sensitivity analysis and meta-regression)

For sensitivity analysis, a new meta-analysis was conducted to determine whether the effect size had changed whenever research was deleted. However, the deleted study was considered when result of the new meta-analysis differed from that of the previous one to influence the total effect size. Influence analysis was conducted using Stata/SE with the metaninf command of NMA. Meta-regression was performed to determine which factors may contribute to the heterogeneity between included RCTs.

## Results

### Study retrieval results

**Fig. 1:**
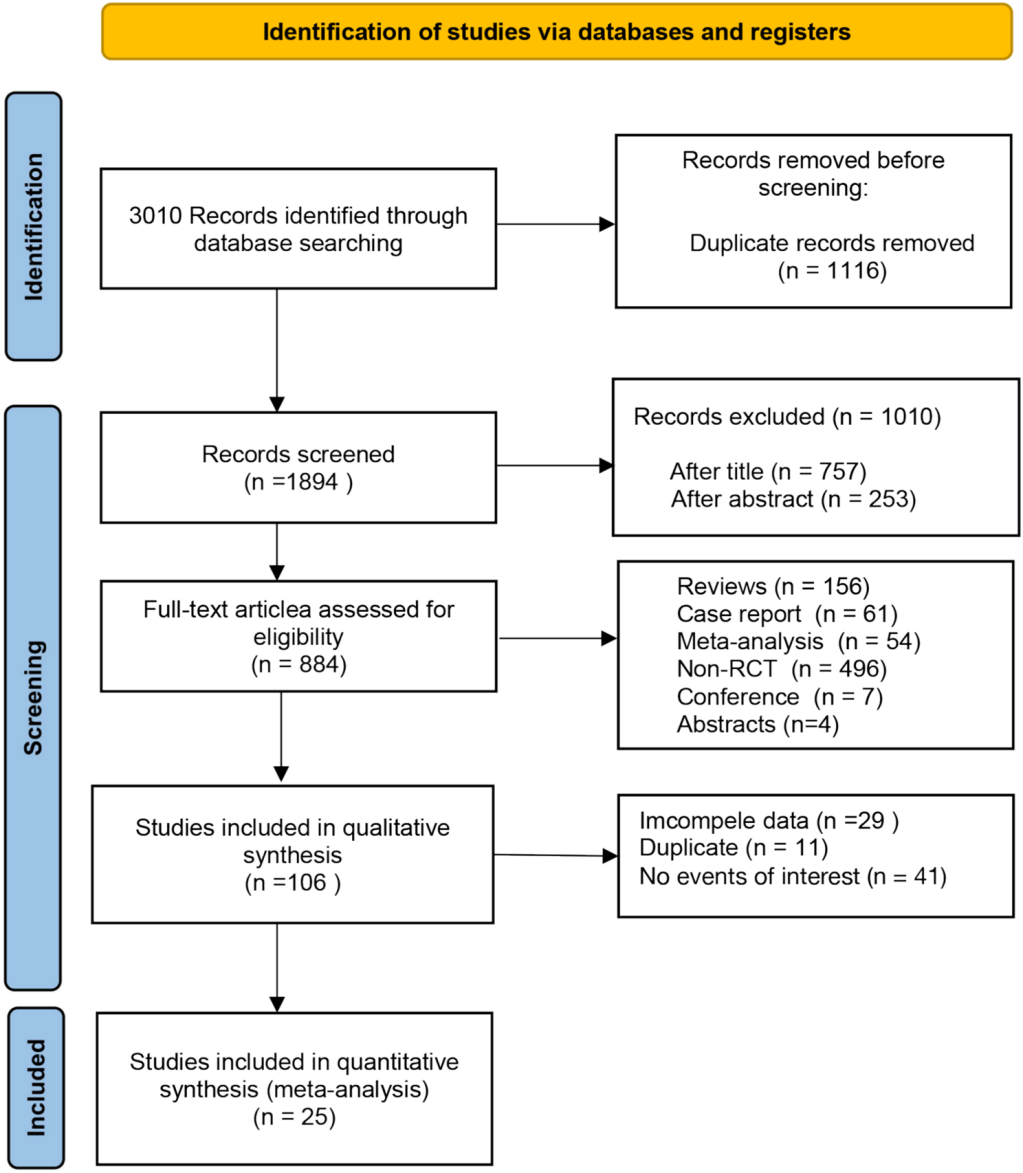
Flowchart of study selection and design

A total of 3010 potentially relevant studies were screened in this study with a comprehensive search strategy, of which 1116 were duplicates and 106 records were eligible for further full-text screening. Based on the inclusion and exclusion criteria, 25 RCTs (*n* = 15,216) evaluating treatments with 17 different drugs were included in the NMA [19–43]. Figure 1 showed the flowchart for the literature screening and inclusion process.

### Systematic review and characteristics

**Fig. 2:**
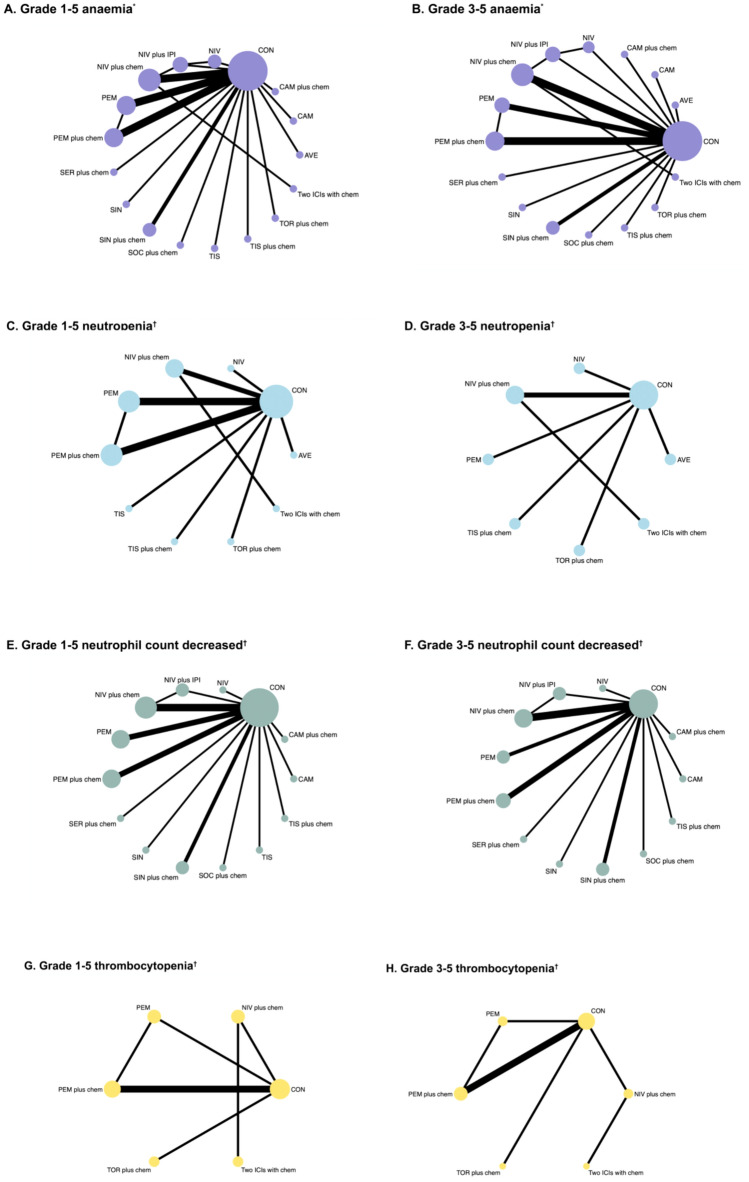

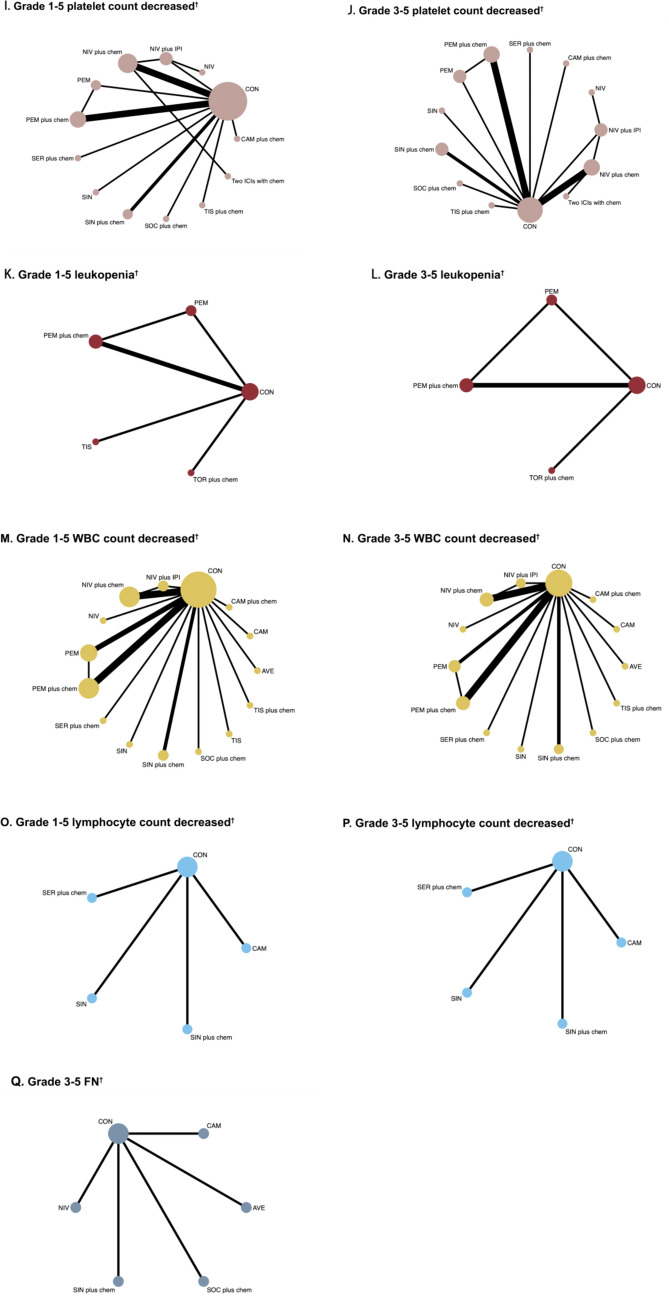
Network plots of eligible direct comparisons (primary and secondary outcomes). Each circular node represents a type of treatment. The circle size is proportional to the total number of patients. The width of lines is proportional to the number of studies performing head-to-head comparisons in the same study. ^*^Primary outcomes. ^†^Secondary outcomes. ICI immune checkpoint inhibitor, AVE avelumab, CAM camrelizumab, CAM plus chem camrelizumab plus chemotherapy, CON chemotherapy with/without placebo, NIV nivolumab, NIV plus IPI nivolumab plus ipilimumab, NIV plus chem nivolumab plus chemotherapy, PEM pembrolizumab, PEM plus chem pembrolizumab plus chemotherapy, SER plus chem serplulimab plus chemotherapy; SIN sintilimab, SIN plus chem sintilimab plus chemotherapy, SOC plus chem socazolimab plus chemotherapy, TIS tislelizumab, TIS plus chem tislelizumab plus chemotherapy, TOR plus chem toripalimab plus chemotherapy, Two ICIs with chem two ICI drugs with chemotherapy

Table 1 showed 25 RCTs published between 2018 and 2024. Four studies (16.00%) were phase II trials and 21 studies (84.00%) were phase III trials. Seventeen studies (68.00%) were multinational (MN) trials, followed by China (*n* = 7) and the USA (*n* = 1). The types of cancer tested in these studies included esophageal cancer (*n* = 12), gastric or gastro-oesophageal junction cancer (*n* = 10), hepatocellular carcinoma (*n* = 2) and biliary tract cancer (*n* = 1). Two groups dominated the study (*n* = 23, 92.00%) and only 8.00% (*n* = 2) had three groups. The control group was chemotherapy with/without placebo. Figure 2 showed the general network plots for 25 RCTs with hematologic safety assessment.

**Table 1:** Baseline characteristics of 25 RCTs for Bayesian NMA by cancer type

First author, year	Region	Tumour type	Stage	Clinical registration number	Trial phase	Arm	Sample Size	Age [Median (min, max)]	Sex (Male/Female)	Drug	CTCAE version
Bang, [19]	MN	Gastric or gastro- oesophageal junction cancer	Unresectable; recurrent; locally advanced; metastatic	NCT02625623	III	1	185	59 (29–86)	140/45	Avelumab	NCI-CTCAE v4.03
						2	186	61 (18–82)	127/59	Paclitaxel	
										Irinotecan	
Chung, [20]	MN	Gastric or gastroesophageal junction cancer	Advanced	NCT03019588	III	1	47	61 (32–75)	32/15	Pembrolizumab	RECIST v1.1
						2	47	61 (37–91)	37/10	Paclitaxel	
Doki, [21]	MN	Esophageal squamous cell carcinoma	Previously untreated; unresectable advanced; recurrent; metastatic	NCT03143153	III	1	321	64 (40–90)	253/68	Nivolumab	NCI-CTCAE v4.0
										5-fluorouracil	
										Cisplatin	
						2	325	63 (28–81)	269/56	Nivolumab	
										Ipilimumab	
						3	324	64 (26–81)	275/49	5-fluorouracil	
										Cisplatin	
Hegewisch-Becker, [22]	MN	Gastric or Gastroesophageal Junction Adenocarcinoma	Metastatic; locally advanced or unresectable	NCT03662659	II	1	138	62 (24–79)	94/44	Nivolumab	NCI-CTCAE v5.0
										Oxaliplatin	
										Capecitabin	
						2	136	63 (23–84)	98/38	Nivolumab	
										Relatlimab	
										Oxaliplatin	
										Capecitabin	
Huang, [43]	China	Oesophageal squamous cell carcinoma	Advanced	NCT03099382	III	1	228	60 (54–65)	208/20	Camrelizumab	NCI-CTCAE v4.0.3
						2	220	60 (54–65)	192/28	Docetaxel	
										Irinotecan	
Janjigian, [23]	MN	Gastric cancer/gastroesophageal junction cancer/oesophageal adenocarcinoma	Metastatic; locally advanced; locally recurrent	NCT03615326	III	1	789	62 (54–69)	540/249	Nivolumab	NCI-CTCAE v4.0
										Oxaliplatin	
										Capecitabin	
						2	792	61 (53–68)	560/232	Oxaliplatin	
										Capecitabin	
Kang, [24]	MN	Gastric or gastro-oesophageal junction cancer	Advanced	NCT02746796	III	1	362	64 (25–86)	253/109	Nivolumab	NCI-CTCAE v4.0
										Oxaliplatin	
						2	362	65 (27–89)	270/92	Placebo	
										Oxaliplatin	
Kang, [25]	MN	Gastric or gastro-oesophageal junction cancer	Stage IIIA-IIIC	NCT03006705	III	1	377	61 (51–70)	267/110	Nivolumab	NCI-CTCAE v4.0
										Capecitabin	
						2	378	61 (51–69)	263/115	Placebo	
										Capecitabin	
Kaseb, [26]	USA	Hepatocellular carcinoma	Resectable	NCT03222076	II	1	13	64 (56–68)	11/2	Nivolumab	RECIST v1.1
						2	14	62 (53–72)	8/6	Nivolumab	
										Ipilimumab	
Kato, [29]	MN	Oesophageal squamous cell carcinoma	Stage II-IV	NCT02569242	III	1	210	64 (57–69)	179/31	Nivolumab	NCI-CTCAE v4.0
						2	209	67 (57–72)	185/24	Paclitaxel	
										Docetaxel	
Kelley, [28]	MN	Biliary tract cancer	Locally advanced or metastatic	NCT04003636	III	1	533	64 (57–71)	280/253	Pembrolizumab	RECIST v1.1
										Gemcitabin	
										Cisplatin	
						2	536	63 (55–70)	272/264	Saline placebo	
										Gemcitabin	
										Cisplatin	
Kojima, [29]	MN	Esophageal Cancer	Metastatic; local advanced	NCT02559687	III	1	314	63 (23–84)	273/41	Pembrolizumab	RECIST V1.1
						2	314	62.0 (24–84)	271/43	Paclitaxel	
										Docetaxel	
										Irinotecan	
Li, [30]	China	Esophageal squamous cell carcinoma	Stage II-IVa	NCT04460066	II	1	32	61 (53–72)	23/9	Socazolimab	NCI-CTCAE v5.0
										Paclitaxel	
										Cisplatin	
						2	32	63 (47–74)	28/4	Placebo	
										Paclitaxel	
										Cisplatin	
Lu, [31]	MN	Oesophageal squamous cell carcinoma	Metastatic; Local advanced	NCT03748134	III	1	327	63 (57–67)	279/48	Sintilimab	RECIST v1.1
										Cisplatin	
										Paclitaxel	
						2	332	63 (56–67)	288/44	Placebo	
										Cisplatin	
										Paclitaxel	
Luo, [32]	China	Hepatocellular carcinoma	Advanced; metastatic	NCT03691090	III	1	298	62 (56–66)	260/38	Camrelizumab	NCI-CTCAE v4.03
										Paclitaxel	
										Cisplatin	
						2	298	62 (56–67)	263/35	Placebo	
										Paclitaxel	
										Cisplatin	
Rha, [33]	MN	Gastric cancer	Locally advanced; metastatic; missing	NCT03675737	III	1	790	61 (52–67)	527/263	Pembrolizumab	NCI-CTCAE v4.0
										5-fluorouracil	
										Cisplatin	
										Capecitabin	
										Oxaliplatin	
						2	789	62 (52–69)	544/245	Placebo	
										5-fluorouracil	
										Cisplatin	
										Capecitabin	
										Oxaliplatin	
Shen, [34]	MN	Esophageal squamous cell carcinoma	Metastatic or locally advanced	NCT03430843	III	1	256	62(40–86)	217/39	Tislelizumab	NCI-CTCAE v4.03
						2	256	63(35–81)	215/41	Paclitaxel	
										Docetaxel	
										Irinotecan	
Shitara, [35]	MN	Gastric or gastroesophageal junction cancer	Metastatic or locally advanced	NCT02370498	III	1	296	62.5 (54–70)	202/94	Pembrolizumab	RECIST v1.1
						2	296	60.0 (53–68)	208/88	Paclitaxel	
Shitara [36]	MN	Gastric cancer	Locally advanced/ unresectable or metastatic	NCT02494583	III	1	256	61.0 (20–83)	180/76	Pembrolizumab	NCI-CTCAE v4.0
						2	257	62.0 (22–83)	195/62	Pembrolizumab	
										Cisplatin	
										5-fluorouracil	
										Capecitabin	
						3	250	62.5 (23–87)	179/71	Cisplatin	
										5-fluorouracil	
										Capecitabin	
Song, [37]	China	Esophageal squamous cell carcinoma	Distantly metastatic or Locally advanced	NCT03958890	III	1	368	64 (57–68)	317/51	Srplulimab	RECIST v1.1
										5-fluorouracil	
						2	183	64 (57–68)	153/30	Placebo	
										Cisplatin	
										5-fluorouracil	
Sun, [38]	MN	Esophageal squamous cell carcinoma	Metastatic or unresectable locally advanced	NCT03189719	III	1	373	64 (28–94)	306/67	Pembrolizumab	NCI-CTCAE v4.0
										5-fluorouracil	
										Cisplatin	
						2	376	62 (27–89)	319/57	Saline placebo	
										5-fluorouracil	
										Cisplatin	
Wang, [39]	China	Esophageal squamous cell carcinoma	Metastatic; locally advanced not amendable for curative therapy; stage I/II-IV	NCT03829969	III	1	257	63 (20–75)	217/40	Toripalimab	NCI-CTCAE v5.0
										Paclitaxel	
										Cisplatin	
						2	257	62 (40–74)	220/37	Placebo	
										Paclitaxel	
										Cisplatin	
Xu, [40]	China	Esophageal squamous cell carcinoma	Advanced or metastatic; stage IIIA-IV	NCT03116152	II	1	95	60 (54–64)	88/7	Sintilimab	RECIST v1.1
						2	95	60 (54–64)	84/11	Irinotecan	
										Paclitaxel	
Xu, [41]	China	Gastric or Gastroesophageal Junction Cancer	Metastatic or locally advanced	NCT03745170	III	1	327	62 (55–67)	253/74	Sintilimab	RECIST v1.1
										Capecitabin	
										Oxaliplatin	
						2	323	60 (52–67)	230/93	Placebo	
										Capecitabin	
										Oxaliplatin	
Xu, [42]	MN	Oesophageal squamous cell carcinoma	Locally advanced or metastatic	NCT03783442	III	1	326	64 (59–68)	282/44	Tislelizumab	NCI-CTCAE v4.03
										Cisplatin	
						2	323	65 (58–70)	281/42	Placebo	
										Cisplatin	

*CTCAE* Common Terminology Criteria for Adverse Events, *MN* multinational

### Risk of bias assessment (ROB)

**Fig. 3:**
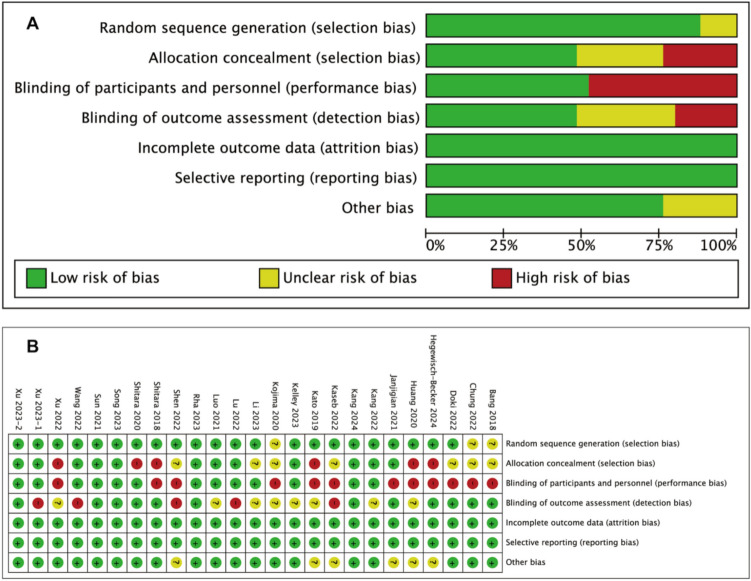
Risk of bias assessment. **A** Risk of bias across all studies. **B** Risk of bias for each item in each study

Figure 3 summarized the ROB for the 25 RCTs included in this study. Seven trials (28%) showed a high ROB due to lack of allocation concealment, investigator blinding, participant blinding, or blinding of results [22, 26, 27, 34, 35, 40, 43]. Nine trials (36%) with a moderate ROB [24, 25, 28, 30, 33, 37, 38, 42, 44] and nine trials (36%) with a low ROB [19–21, 23, 29, 31, 36, 39, 41].

### Outcomes of the network meta-analysis

#### Primary outcomes

##### Safety profile

**Fig. 4:**
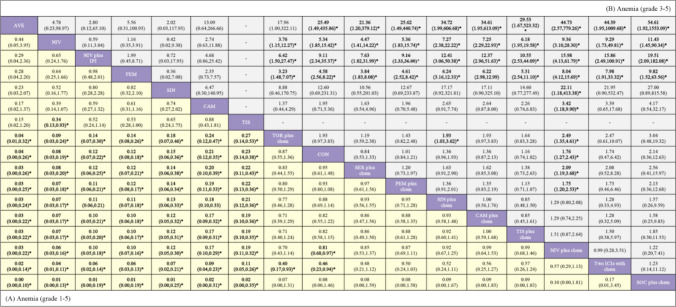
Safety profile according to the drug based network meta-analysis in the consistency model. The lower left corner shows grade 1–5 anemia, and the upper right corner shows grade 3–5 anemia. Each cell of the safety profile contains the pooled odds ratios and 95% credibility intervals for grade 1–5 and 3–5 anemia; significant results are in bold and with an asterisk

In the safety profile presented in Fig. 4, ICI monotherapy and nivolumab plus ipilimumab had a lower risk of anemia than combinations of one ICI with chemotherapy, chemotherapy with or without placebo, and two ICIs with chemotherapy. Furthermore, about the risk of grades 1–5 anemia, nivolumab exhibited a significantly lower risk compared to tislelizumab; toripalimab plus chemotherapy had a noticeably decreased risk than socazolimab plus chemotherapy; chemotherapy with or without placebo was linked to a lower risk compared to the combination of two ICIs with chemotherapy or socazolimab plus chemotherapy. In terms of the risk of grades 3–5 anemia, toripalimab plus chemotherapy showed a lower risk than sintilimab plus chemotherapy and nivolumab plus chemotherapy; nivolumab plus chemotherapy was associated with a higher risk compared to serplulimab plus chemotherapy, chemotherapy with or without placebo, pembrolizumab plus chemotherapy, and toripalimab plus chemotherapy.

##### Incidence

The incidence of grade 1–5 anemia for avelumab, pembrolizumab, camrelizumab, nivolumab, tislelizumab, and sintilimab was 0.54, 3.52, 10.53, 3.15, 10.98, and 8.51%, respectively. For the combination of one ICI with chemotherapy, the incidence rates were: nivolumab plus chemotherapy (21.82%), pembrolizumab plus chemotherapy (41.31%), socazolimab plus chemotherapy (100%), camrelizumab plus chemotherapy (76.85%), serplulimab plus chemotherapy (75.92%), toripalimab plus chemotherapy (78.21%), sintilimab plus chemotherapy (60.76%), and tislelizumab plus chemotherapy (53.40%). For nivolumab combined with ipilimumab, the incidence rate was 4.46%. When two ICIs were used in combination with chemotherapy, the incidence rate was 19.12%. In the control group, which included chemotherapy with or without placebo, the incidence was 37.15%.

In terms of grade 3–5 anemia (Table 2), the incidence of avelumab, pembrolizumab, camrelizumab, nivolumab, and sintilimab was 0, 1.74, 2.63, 1.80, and 0%, respectively. For the combination of one ICI with chemotherapy, the observed incidence rates included 5.72% for nivolumab plus chemotherapy, 15.10% for pembrolizumab plus chemotherapy, and 12.50% for socazolimab plus chemotherapy. Additionally, camrelizumab plus chemotherapy had a rate of 17.45%, while serplulimab plus chemotherapy, toripalimab plus chemotherapy were associated with rates of 17.54 and 10.90%, respectively. For sintilimab plus chemotherapy and tislelizumab plus chemotherapy, the rates were 12.52 and 14.51%, respectively. Nivolumab plus ipilimumab showed an incidence rate of 0.6%. Furthermore, when two ICIs were used alongside chemotherapy, the incidence was reported at 3.68%. In the control group receiving chemotherapy with or without placebo, the rate of anemia reached 9.72%.

**Table 2:** Estimated incidence and ranks, anemia

Intervention	Grade 1–5		Grade 3–5	
	Incidence (%, 95%CI)	SUCRA (%)	Incidence (%, 95%CI)	SUCRA (%)
AVE	0.54 (0.01, 2.97)	95.70	0 (0, 1.99)	92.80
NIV	3.15 (1.20, 6.65)	91.70	1.80 (0.42, 4.78)	88.00
NIV plus IPI	4.46 (2.25, 6.67)	83.50	0.6 (0.07, 3.51)	86.00
PEM	3.52 (2.32, 4.72)	83.40	1.74 (0.86, 2.62)	81.40
SIN	8.51 (3.36, 16.28)	77.80	0 (0, 4.00)	79.10
CAM	10.53 (6.55, 14.51)	69.70	2.63 (0.88, 5.87)	62.30
TIS	10.98 (7.16, 14.80)	66.80	/	/
TOR plus chem	78.21 (73.17, 83.25)	48.30	10.9 (7.1, 14.7)	57.10
CON	37.15 (36.03, 38.27)	44.80	9.72 (9.01, 10.43)	41.50
SER plus chem	75.92 (71.65, 80.19)	37.20	17.54 (13.74, 21.34)	50.10
PEM plus chem	41.31 (39.10, 43.52)	35.70	15.1 (13.49, 16.71)	40.70
SIN plus chem	60.76 (56.98, 64.54)	30.90	12.52 (9.99, 15.05)	21.50
CAM plus chem	76.85 (72.05, 81.65)	25.70	17.45 (13.16, 21.74)	22.30
TIS plus chem	53.40 (47.97, 58.83)	25.10	14.51 (10.69, 18.33)	31.30
NIV plus chem	21.82 (20.00, 23.64)	22.60	5.72 (4.68, 6.76)	10.30
Two ICI drugs with chem	19.12 (12.34, 25.90)	7.80	3.68 (0.75, 9.57)	19.40
SOC plus chem	100.00 (88.53, 100.00)	3.30	12.5 (3.08, 30.30)	16.20

/: No relevant data were recorded in the original literature

*CI* confidence interval, *SUCRA* surface under the cumulative probability ranking curve, *AVE* avelumab, *CAM* camrelizumab, *CAM plus chem* camrelizumab plus chemotherapy, *CON* chemotherapy with/without placebo, *NIV* nivolumab, *NIV plus IPI* nivolumab plus ipilimumab, *NIV plus chem* nivolumab plus chemotherapy, *PEM* pembrolizumab, *PEM plus chem* pembrolizumab plus chemotherapy, *SER plus chem* serplulimab plus chemotherapy, *SIN* sintilimab, *SIN plus chem* sintilimab plus chemotherapy, *SOC plus chem* socazolimab plus chemotherapy, *TIS* tislelizumab, *TIS plus chem* tislelizumab plus chemotherapy, *TOR plus chem* toripalimab plus chemotherapy, *Two ICIs with chem* two ICI drugs with chemotherapy

SUCRA values can range from 0 to 100%. Lower rates represent a less safe option (negative outcome)

##### Rank probabilities

**Fig. 5:**
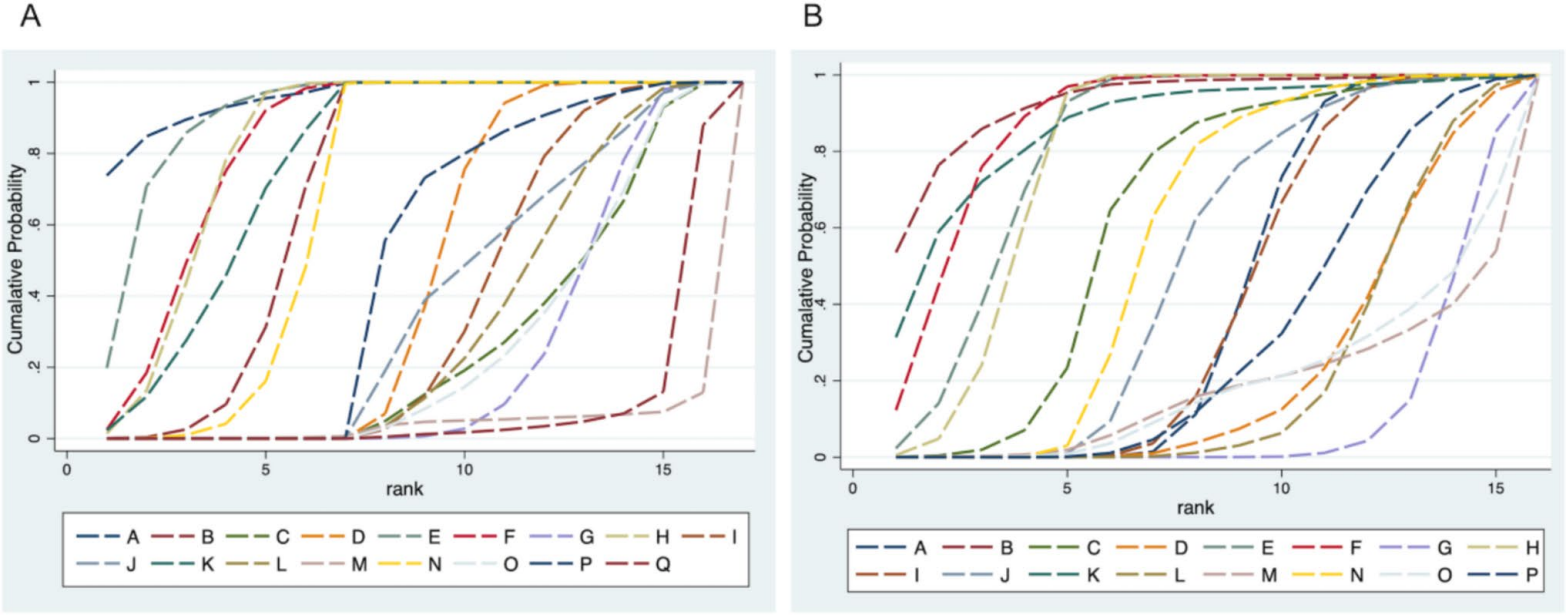
Ranking curves of grade 1–5 (**A**), ranking curves of grade 3–5 (**B**) according to the drug based network meta-analysis in the consistency model. Ranking curves indicate the probability of the highest risk of grade 1–5 anemia and grade 3–5 anemia, the second highest, the third highest, and so on. ICI immune checkpoint inhibitor. (A): A avelumab, B camrelizumab, C = camrelizumab plus chemotherapy, D chemotherapy with/without placebo, E nivolumab, F nivolumab plus ipilimumab, G nivolumab plus chemotherapy, H pembrolizumab, I pembrolizumab plus chemotherapy, J serplulimab plus chemotherapy, K sintilimab, L sintilimab plus chemotherapy, M socazolimab plus chemotherapy, N tislelizumab, O tislelizumab plus chemotherapy, P toripalimab plus chemotherapy, Q two ICI drugs with chemotherapy. **B**: A chemotherapy with/without placebo, B avelumab, C = camrelizumab, D = camrelizumab plus chemotherapy, E nivolumab, F nivolumab plus ipilimumab, G nivolumab plus chemotherapy, H pembrolizumab, I pembrolizumab plus chemotherapy, J serplulimab plus chemotherapy, K sintilimab, L sintilimab plus chemotherapy, M socazolimab plus chemotherapy, N toripalimab plus chemotherapy, O two ICI drugs with chemotherapy, P tislelizumab plus chemotherapy

Based on the SUCRA values (Table 2), the ranking overview aligns with the results of the original NMA. Figure 5A highlighted that avelumab was linked to the best safety ranking for grade 1–5 anemia (probability = 95.70%). This was followed by nivolumab (91.70%), nivolumab plus ipilimumab (83.50%), pembrolizumab (83.40%), sintilimab (77.80%), camrelizumab (69.70%), and tislelizumab (66.80%). Among the regimens involving chemotherapy, toripalimab plus chemotherapy ranks higher (48.30%), followed by chemotherapy with/without placebo (44.80%) and serplulimab plus chemotherapy (37.20%). Pembrolizumab plus chemotherapy (35.70%) and sintilimab plus chemotherapy (30.90%) occupy intermediate ranks. Lower safety rankings were seen for camrelizumab plus chemotherapy (25.70%), tislelizumab plus chemotherapy (25.10%), and nivolumab plus chemotherapy (22.60%). Two ICIs with chemotherapy had a notably low ranking (7.80%), with socazolimab plus chemotherapy ranked last (3.30%).

Figure 5B illustrated the safety rankings for grade 3–5 anemia. Avelumab again leads with the highest probability (92.80%), followed by nivolumab (88.00%) and nivolumab plus ipilimumab (86.00%). Pembrolizumab ranks fourth (81.40%), with sintilimab (79.10%) and camrelizumab (62.30%) rounding out the higher safety tiers. For combinations involving chemotherapy, toripalimab plus chemotherapy ranks higher (57.10%), followed by serplulimab plus chemotherapy (50.10%) and chemotherapy with/without placebo (41.50%). Pembrolizumab plus chemotherapy (40.70%) and tislelizumab plus chemotherapy (31.30%) occupy intermediate ranks, with camrelizumab plus chemotherapy (22.30%) and sintilimab plus chemotherapy (21.50%) scoring lower. Two ICIs with chemotherapy (19.40%) and socazolimab plus chemotherapy (16.20%) rank near the bottom, with nivolumab plus chemotherapy ranking the lowest (10.30%).

##### Secondary outcomes

We observed broader safety profiles across all secondary outcomes for monotherapy with any single ICI and for nivolumab plus ipilimumab. These treatments demonstrated superior safety compared to combinations of one ICI drug with chemotherapy, chemotherapy with or without placebo, and two ICI drugs with chemotherapy. Other findings on this basis were presented below.

#### Neutropenia

The analysis of grade 1–5 neutropenia comprised a total of 13 RCTs with 8,730 participants. Toripalimab plus chemotherapy was significantly linked to an increased risk of neutropenia compared to chemotherapy with or without placebo, pembrolizumab plus chemotherapy, and nivolumab plus chemotherapy (Fig. S1A). Avelumab ranked as the safest treatment (SUCRA 91.6%), while tislelizumab was the lowest-ranked single ICI (74.7%). Toripalimab plus chemotherapy had the worst overall ranking (1.4%) (Fig. S1B). For grade 3–5 neutropenia, 8 trials involving 5,084 participants were analyzed. Toripalimab plus chemotherapy was significantly linked to a higher risk compared to tislelizumab plus chemotherapy and chemotherapy with or without placebo (Fig. S1A). Nivolumab achieved the highest safety ranking (93.3%), while pembrolizumab ranked the lowest (17.4%) (Fig. S1C).

#### Neutrophil count decreased

The analysis of grade 1–5 neutrophil count decreased included 20 trials involving 13,295 participants. Tislelizumab was found to be safer than both camrelizumab and sintilimab (Fig. S2A). Among the treatments, tislelizumab ranked the highest (92.3%). The lowest-ranked single ICI was sintilimab (65.7%), while the worst overall ranking was for camrelizumab plus chemotherapy (17.5%) (Fig. S2B). For grade 3–5 neutrophil count decreased, and data from 18 trials involving 12,660 participants were analyzed. Nivolumab achieved the highest safety ranking (89.4%). Once again, sintilimab was the lowest-ranked single ICI (70%). The worst overall ranking was observed for pembrolizumab plus chemotherapy (12.6%) (Fig. S2C).

#### Thrombocytopenia

The analysis of grade 1–5 and 3–5 thrombocytopenia included 6 trials with a total of 4562 participants. Toripalimab plus chemotherapy was significantly linked to an increased risk of grade 1–5 thrombocytopenia compared to chemotherapy with or without placebo and pembrolizumab plus chemotherapy (Fig. S3A). Pembrolizumab ranked as the safest treatment (99.9%), while toripalimab plus chemotherapy had the worst ranking (6.1%) (Fig. S3B). For grade 3–5 thrombocytopenia, pembrolizumab remained the top-ranked treatment (96.1%). In contrast, nivolumab plus chemotherapy ranked the lowest (15.9%) (Fig. S3C).

#### Platelet count decreased

The analysis of grade 1–5 and 3–5 platelet count decreased included 17 trials with a total of 11,710 participants. For grade 1–5 platelet count decreased, and the combination of two ICI drugs with chemotherapy was shown to be safer than socazolimab plus chemotherapy (Fig. S4A). Pembrolizumab ranked as the safest treatment (95%), while nivolumab was the lowest-ranked single ICI (66.6%). Socazolimab plus chemotherapy had the worst overall ranking (3.6%) (Fig. S4B). For grade 3–5 platelet count decreased, and pembrolizumab again achieved the highest ranking (87.9%). Among single ICIs, sintilimab ranked the lowest (33.8%). Tislelizumab plus chemotherapy had the worst overall ranking (16.3%) (Fig. S4C).

#### Leukopenia

The analysis of grade 1–5 leukopenia included 4 trials (2497 participants). Chemotherapy with or without placebo, pembrolizumab plus chemotherapy were both safer than toripalimab plus chemotherapy (Fig. S5A). Pembrolizumab ranked the highest in safety (98.4%), while toripalimab plus chemotherapy ranked the lowest (0.1%). For grade 3–5 leukopenia, 3 trials (2002 participants) (Fig. S5B). Pembrolizumab was again the top-ranked treatment (84.7%), whereas toripalimab plus chemotherapy had the lowest ranking (32.2%) (Fig. S5C).

#### White blood cell (WBC) count decreased

The analysis of grade 1–5 WBC count decreased included 21 trials with 13,834 participants. Nivolumab was found to be safer than Sintilimab. Chemotherapy with or without placebo was the safest option compared to pembrolizumab with chemotherapy, nivolumab plus chemotherapy (Fig. S6A). Nivolumab ranked highest in safety (93.4%), while the lowest-ranked single ICI was sintilimab (65.3%). The overall lowest-ranked treatment was socazolimab plus chemotherapy (11.5%) (Fig. S6B). For grade 3–5 WBC count decreased, and 20 trials involving 13,248 participants were included. Tislelizumab plus chemotherapy showed greater safety compared to pembrolizumab plus chemotherapy, nivolumab plus chemotherapy, serplulimab plus chemotherapy, and socazolimab plus chemotherapy. Similarly, sintilimab plus chemotherapy was safer than pembrolizumab plus chemotherapy, nivolumab plus chemotherapy, and serplulimab plus chemotherapy. Chemotherapy with or without placebo was also safer than pembrolizumab plus chemotherapy. Camrelizumab ranked highest in safety (90.3%), while avelumab was the lowest-ranked single ICI (73.1%). The worst overall ranking was observed for socazolimab plus chemotherapy (7.1%) (Fig. S6C).

#### Lymphocyte count decreased

The analysis of grade 1–5 and 3–5 lymphocyte count decreased included 4 trials involving 1,838 participants (Fig. S7A). For grade 1–5 lymphocyte count decreased, sintilimab ranked highest in safety (75.30%), while serplulimab plus chemotherapy ranked the lowest (30.80%) (Fig. S7B). In contrast, for grade 3–5 lymphocyte count decreased, serplulimab plus chemotherapy had the highest ranking (69.80%), whereas serplulimab alone ranked the lowest (31.90%) (Fig. S7C).

#### Febrile neutropenia (FN)

Overall, FN was primarily observed in grades 3–5, based on data from 5 trials involving 1949 participants (Fig. S8A). Nivolumab ranked as the safest treatment (69.8%), while socazolimab plus chemotherapy had the lowest ranking (31.9%) (Fig. S8B).

#### Heterogeneity and consistency

The results indicated that there was no inconsistency among the included RCTs in terms of global consistency (Table S3). For example, the *p* value for studies on grade 1–5 anemia was 0.7773, while on grade 3–5 anemia was 0.4606. In terms of local inconsistency, there were no statistically significant differences (*P* > 0.05) in the discrepancies between direct and indirect comparisons of studies assessing grade 1–5 and 3–5 anemia. This suggests the absence of local inconsistency. Similarly, subgroup analyses revealed no evidence of either overall or local inconsistency. The heterogeneity of hematologic toxicities graded as 1–5 and 3–5 was evaluated and was summarized in Table S1. The Ι^2^ values indicated heterogeneity across most toxicity categories, except for grade 3–5 thrombocytopenia, platelet count decreased, and lymphocyte count decreased.

#### Subgroup analysis and meta-regression

Subgroup analysis showed no significant heterogeneity among the included studies except for the ICI regimen (Table 3, Fig. S9–S15). Meta-regression also observed the ICI regimen as a significant source of heterogeneity in all treatment comparisons (*p* < 0.001) (Table 4). Subgroup analysis was performed according to tumor type, country category, study phase, ICI regimen, control group, chemotherapy regimen and ICI plus different chemotherapy regimens. We found that patients with gastric or gastro-oesophageal cancer had a higher risk of hematologic toxicity than patients with esophageal cancer. The risk of hematologic toxicity was higher in China than in MN. Patients with Phase III have a higher risk of hematologic toxicity than those with phase II. Patients receiving ICI plus chemotherapy have a higher risk of hematologic toxicity than ICI. Patients in the control group who received chemotherapy with placebo had a higher risk of hematologic toxicity than those who received chemotherapy without placebo. The risk of hematologic toxicity due to different chemotherapy regimens was from high to low as paxane-based plus platinum-based, platinum-based plus 5-fluorouracil, platinum-based, taxane-based plus irinotecan, and taxane-based. The risk of hematologic toxicity due to ICI plus different chemotherapy regimens was from high to low as ICI plus taxane-based and platinum-based, ICI plus platinum-based and 5-fluorouracil, ICI plus platinum-based.

**Table 3:** Subgroup analysis of OR and heterogeneity

Subgroup analysis	Grade	No. of studies	OR (95% CI)	Heterogeneity	
				I^2^ (%)	*p* value
Tumor type					
Esophageal cancer	1–5	12	0.45 (0.27, 0.76)	93.4	0.000
	3–5	11	0.68 (0.46, 1.01)	72.2	0.000
Gastric or gastro-oesophageal junction cancer	1–5	10	0.63 (0.42, 0.93)	89.1	0.000
	3–5	9	0.84 (0.52, 1.37)	77.7	0.000
Country category					
MN	1–5	17	0.52 (0.37, 0.73)	92.4	0.000
	3–5	15	0.75 (0.53, 1.04)	78.4	0.000
China	1–5	7	0.67 (0.38. 1.19)	89.1	0.000
	3–5	7	0.97 (0.67. 1.40)	48.9	0.068
Study phase					
Phase II	1–5	4	0.55 (0.16. 1.91)	74.4	0.008
	3–5	4	0.82 (0.20. 3.44)	44.6	0.164
Phase III	1–5	21	0.57 (0.42. 0.77)	91.9	0.000
	3–5	19	0.81 (0.62. 1.06)	75.7	0.000
ICI regimen					
ICI plus chemotherapy vs CON	1–5	15	1.14 (1.04, 1.24)	2.8	0.420
	3–5	15	1.15 (0.98, 1.36)	37.8	0.069
ICI vs CON	1–5	9	0.15 (0.11, 0.20)	33.7	0.148
	3–5	7	0.22 (0.11, 0.41)	44.5	0.094
Control group					
Chemotherapy without placebo	1–5	13	0.37 (0.23. 0.60)	93.4	0.000
	3–5	11	0.54 (0.31. 0.94)	79.1	0.000
Chemotherapy with placebo	1–5	12	0.90 (0.68. 1.18)	80.9	0.000
	3–5	12	1.01 (0.79. 1.31)	63.9	0.000
Chemotherapy regimen					
Taxane-based	1–5	3	0.185 (0.116, 0.253)	/	/
	3–5	2	0.057 (0.036, 0.077)	/	/
Platinum-based	1–5	6	0.340 (0.186, 0.494)	98.902	0.000
	3–5	6	0.097 (0.044, 0.150)	97.702	0.000
Taxane-based plus irinotecan	1–5	5	0.286 (0.191, 0.380)	91.828	0.000
	3–5	4	0.062 (0.045, 0.079)	0.000	0.633
Platinum-based plus 5-fluorouracil	1–5	5	0.400 (0.245, 0.555)	98.099	0.000
	3–5	5	0.117 (0.071, 0.163)	90.546	0.000
Taxane-based plus platinum-based	1–5	4	0.759 (0.699, 0.818)	74.735	0.008
	3–5	4	0.120 (0.091, 0.148)	40.659	0.168
ICI plus different chemotherapy regimen					
ICI plus platinum-based	1–5	7	0.312 (0.180, 0.444)	98.629	0.000
	3–5	7	0.095 (0.045, 0.146)	97.383	0.000
ICI plus platinum- based and 5-fluorouracil	1–5	4	0.337 (0.296, 0.377)	65.983	0.032
	3–5	4	0.102 (0.080, 0.124)	52.282	0.098
ICI plus taxane-based and platinum-based	1–5	4	0.766 (0.738, 0.794)	/	/
	3–5	4	0.134 (0.103, 0.165)	43.229	0.152

/: When using the “metaprop” to conduct subgroup analysis on single-group data, if the number of included literatures is three or less, the results of the heterogeneity test will not be displayed

*OR* odds ratio, *MN* multinational, *ICI* immune checkpoint inhibitor, *CON* chemotherapy with/without placebo

**Table 4:** Meta-regression analysis of factors affecting heterogeneitya

Variable	Grade 1–5		Grade 3–5	
	Coefficient (95%CI)	*p* value	Coefficient (95%CI)	*p* value
Tumor type	0.89 (− 0.66, 2.45)	0.225	0.45 (− 1.81, 2.71)	0.649
Country category	− 0.78 (− 2.20, 0.63)	0.248	0.67 (− 1.41, 2.76)	0.478
Study phase	3.03 (0.90, 6.97)	0.118	− 1.85 (− 4.15, 0.45)	0.101
ICI regimen	− 2.02 (− 2.65, − 1.38)	*p* < 0.001*	− 1.07 (− 1.50, − 0.63)	*p* < 0.001*
Control group	− 0.50 (− 1.89, 0.90)	0.448	0.46 (− 1.64, 2.56)	0.627
Chemotherapy regimen	3.34 (− 6.69, 13.37)	0.497	1.27 (− 51.31, 53.86)	0.960
ICI plus chemotherapy regimen	4.89 (− 51.93, 61.71)	0.856	1.40 (− 2.77, 5.57)	0.485

^*^Statistically significant at *p* < 0.05

*ICI* immune checkpoint inhibitor, *CI* confidence interval

### Tumor type

#### Esophageal cancer

Overall, the analysis of grade 1–5 anemia in esophageal cancer included 12 trials with 6259 participants (Fig. S16A). Nivolumab and pembrolizumab were safer than tislelizumab. Among combination therapies, pembrolizumab plus chemotherapy was the safest compared to sintilimab plus chemotherapy and nivolumab plus chemotherapy. Chemotherapy with or without placebo showed greater safety than nivolumab plus chemotherapy (Fig. S17A). Nivolumab ranked the highest in safety (93.80%), while the lowest-ranked single ICI was tislelizumab (66.90%). The overall lowest ranking was for socazolimab plus chemotherapy (2.70%) (Fig. S17B).

For grade 3–5 anemia in esophageal cancer, data from 11 trials with 5764 participants were included (Fig. S16B). Toripalimab plus chemotherapy and pembrolizumab plus chemotherapy had lower risks compared to nivolumab plus chemotherapy (Fig. S17A). Nivolumab plus ipilimumab ranked the highest in safety (89.90%), while the lowest-ranked single ICI was camrelizumab (60.1%). The overall lowest ranking was for nivolumab plus chemotherapy (8.50%) (Fig. S17C).

#### Gastric or gastro-oesophageal junction cancer

The gastric or gastro-oesophageal junction cancer subgroup analysis of grades 1–5 combined with anemic pooled 10 trials (7272 participants) (Fig. S16C). Chemotherapy with/without placebo was safer than pembrolizumab plus chemotherapy and two ICI drugs with chemotherapy (Fig. S11A). The highest-ranked treatment was avelumab (98.6%), while the worst-ranked was two ICI drugs with chemotherapy (3.1%) (Fig. S18B). Grade 3–5 combined anemia pooled 9 trials (7181 participants) (Fig. S16D). The highest-ranked treatment was avelumab (96.8%), while the worst-ranked was nivolumab plus chemotherapy (23.5%) (Fig. S18C).

#### Sensitivity analysis

**Fig. 6:**
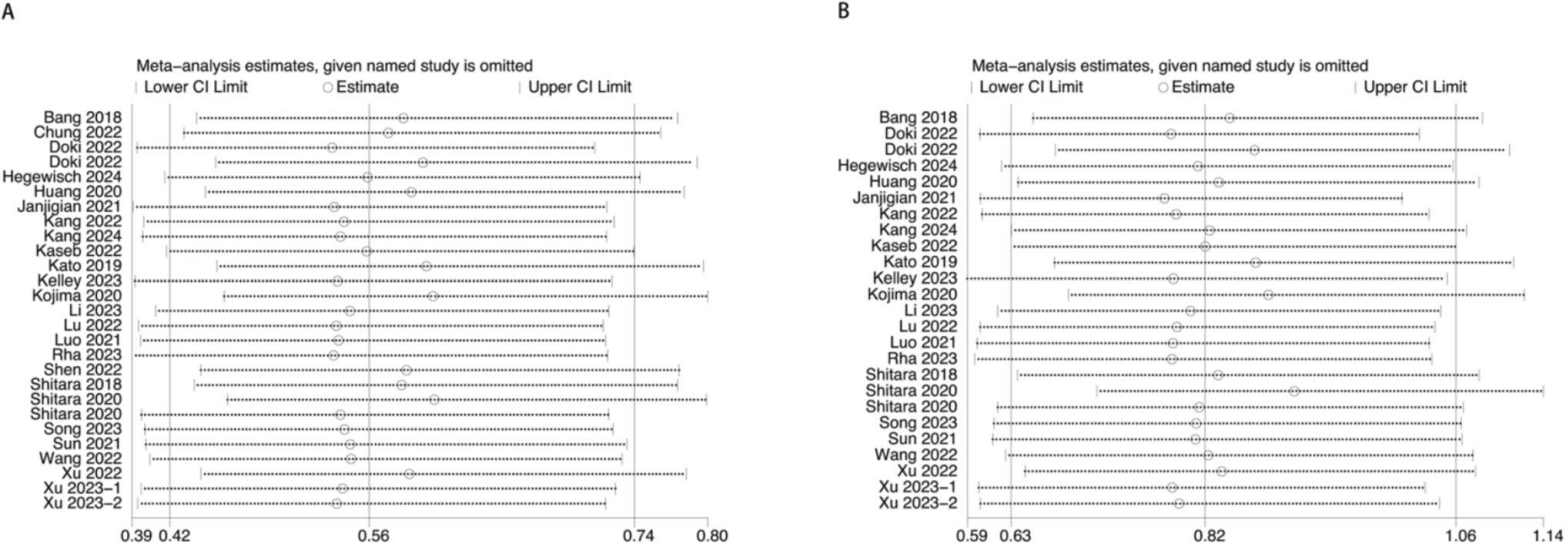
Sensitivity analysis: the influence of single study on the total merger effect. **A** grade 1–5 anemia; **B** grade 3–5 anemia

The results of the sensitivity analyses for the primary outcome were shown in Fig. 6, and the sensitivity analyses for the secondary outcomes were shown in Figs. S19–S26. We found that the points for the combined effect sizes after deleting a study all fell within the 95% confidence interval for the total combined effect size, indicating a low sensitivity and robustness of the findings. The effect of potential sources of heterogeneity was low.

#### Publication bias

**Fig. 7:**
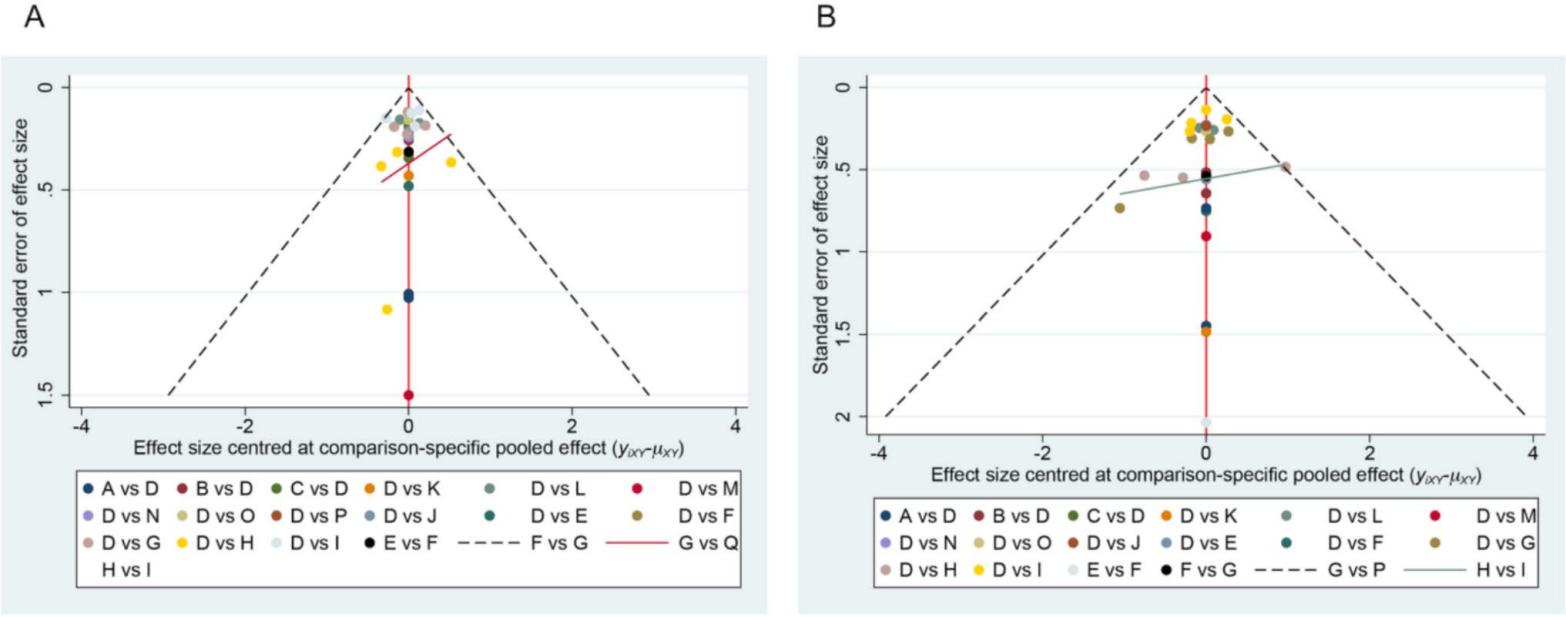
Funnel plot. **A** Funnel plot of adverse reactions occurring in grade 1–5 anemia. **B** Funnel plot of adverse reactions occurring in grade 3–5 anemia. Abbreviations in Fig. 5A: A avelumab, B camrelizumab, C camrelizumab plus chemotherapy, D chemotherapy with/without placebo, E nivolumab, F nivolumab plus ipilimumab, G nivolumab plus chemotherapy, H pembrolizumab, I pembrolizumab plus chemotherapy, J serplulimab plus chemotherapy, K sintilimab, L sintilimab plus chemotherapy, M socazolimab plus chemotherapy, N tislelizumab, O tislelizumab plus chemotherapy, P toripalimab plus chemotherapy, Q two ICI drugs with chemotherapy. Abbreviations in Fig. 5B: A avelumab, B camrelizumab, C camrelizumab plus chemotherapy, D chemotherapy with/without placebo, E nivolumab, F nivolumab plus ipilimumab, G nivolumab plus chemotherapy, H pembrolizumab, I pembrolizumab plus chemotherapy, J serplulimab plus chemotherapy, K sintilimab, L sintilimab plus chemotherapy, M socazolimab plus chemotherapy, N tislelizumab plus chemotherapy, O toripalimab plus chemotherapy, P two ICI drugs with chemotherapy

The corrected-comparison funnel plots for grade 1–5 and 3–5 anemia (primary outcomes) are shown in Fig. 7A, B, indicating that all study points were distributed roughly symmetrically on both sides of the midline, suggesting that the risk of publication bias was less likely. However, the studies of grade 1–5 anemia deviated far from the regression line, suggesting that there may be some small sample events or publication bias.

## Discussion

### Principal findings and strengths

Safety is a critical factor in drug evaluation. Previous meta-analyses focused mainly on the general safety of ICIs [10] or hematologic toxicities across multiple cancer types [6, 45]. However, their clinical applicability was limited, hindering individualized treatment. The prior NMA included only phase III trials from PubMed, Cochrane, and Embase, with a simple search strategy that may have missed early studies crucial to understanding the risks and benefits of these drugs [11]. As a result, a comprehensive safety profile was not achieved. Pharmacokinetics and pharmacodynamics are the primary emphasis of phase I trials, which have small sample sizes. Therefore, to reduce bias and confounding, our study comprised phase II and III studies. Previous NMA on this topic reported hematologic toxicities for only some ICIs, lacking SUCRA probabilities and subgroup analyses [11]. This limited a comprehensive understanding of ICIs’ hematologic toxicities and provided insufficient cancer-specific insights. Some treatment-related AEs, such as severe thrombocytopenia and neutropenia, are life-threatening, necessitating a thorough investigation of ICIs’ toxicity profiles. To our knowledge, this is the first NMA to assess both grade 1–5 and 3–5 hematologic toxicities in patients with digestive system tumors treated with ICIs in phase II and III trials. With stringent inclusion criteria, our NMA showed excellent transitivity, ensuring valid and reliable results. These findings will aid clinicians in managing life-threatening AEs, optimizing trial designs, and refining ICI prescribing practices.

Consistent with previous studies investigating the hematologic toxicities of ICIs [11], our research confirms that combinations of chemotherapy with/without placebo and one or two ICIs with chemotherapy are associated with more severe hematologic toxicities compared to single ICI therapy or nivolumab plus ipilimumab. Notably, we identified several unique findings. First, each treatment has a distinct safety profile, warranting special attention to each ICI drug. The same ICI ranked differently in terms of the risk and probability of hematologic toxicities in grades 1–5 and 3–5. Anemia, the most common hematologic toxicity [6], demonstrated that the risk of grade 1–5 and 3–5 anemia was increased by tislelizumab and camrelizumab, respectively. Among secondary outcomes, tislelizumab had the highest risk of grade 1–5 neutropenia, while pembrolizumab had the highest risk of grade 3–5. Sintilimab had the highest risk for neutrophil count decreased, grade 3–5 platelet count decreased and lymphocyte count decreased, and grade 1–5 WBC count decreased. Nivolumab had the highest risk for grade 1–5 platelet count decreased. Avelumab had the highest risk for grade 3–5 WBC count decreased. Camrelizumab had the highest risk for grade 1–5 lymphocyte count decreased and the highest risk of FN. Second, chemotherapy alone is known for its high hematologic toxicity, and our study found that some ICI-chemotherapy combinations posed a higher risk of hematologic toxicities than chemotherapy alone. Treatment-related AEs with ICIs, compared with chemotherapy, were mainly observed in grade 1–5 anemia, neutropenia, thrombocytopenia, leukopenia, and WBC count decreased. The use of ICIs with chemotherapy requires careful consideration to ensure hematologic safety. This comprehensive analysis highlights the hematologic toxicities that should be monitored with each ICI plus chemotherapy regimen, providing insights distinct from previous studies [11].

- Socazolimab plus chemotherapy: grade 1–5 anemia and platelet count decreased, grade 1–5 and 3–5 WBC count decreased, grade 3–5 FN.
- Toripalimab plus chemotherapy: grade 1–5 neutropenia and thrombocytopenia, grade 1–5 and 3–5 leukopenia.
- Nivolumab plus chemotherapy: grade 3–5 anemia and thrombocytopenia.
- Camrelizumab plus chemotherapy: grade 1–5 neutrophil count decreased.
- Pembrolizumab plus chemotherapy: grade 3–5 neutrophil count decreased.
- Tislelizumab plus chemotherapy: grade 3–5 platelet count decreased.
- Serplulimab plus chemotherapy: grade 1–5 lymphocyte count decreased.

Moreover, we observed that all cases of FN in the studies were classified as grade 3–5. These events mainly occurred in patients receiving chemotherapy with or without placebo, or one ICI with chemotherapy. No cases of FN were reported in patients receiving a single ICI. Once FN occurs, it significantly prolongs hospital stays, increases medical costs, and disrupts treatment plans. FN also raises the risk of severe complications, such as sepsis and other infections, requiring careful attention and proactive management.

Subgroup analyses of gastric or gastro-oesophageal junction cancer and esophageal cancer provided key insights into ICI hematologic toxicities for specific tumor types. These findings aid in developing individualized treatment strategies. Our study showed that avelumab had the best safety profile for anemia in gastric or gastro-oesophageal junction cancer. In contrast, the combination of two ICIs with chemotherapy posed the highest risk of anemia across grade 1–5, while nivolumab plus chemotherapy carried the highest risk for grade 3–5 anemia. For esophageal cancer, socazolimab plus chemotherapy had the highest risk of anemia across grade 1–5, while nivolumab plus chemotherapy demonstrated the greatest risk for grade 3–5 anemia.

### Potential underlying mechanisms

Immune-related hematologic toxicities are rare but potentially life-threatening complications of ICIs. The mechanisms behind these toxicities in digestive system tumors are complex [46]. ICI disrupts immune suppression by blocking the pathways of programmed death ligand 1 (PD-L1), programmed death receptor 1 (PD-1), or cytotoxic T-lymphocyte-associated antigen 4. This enhances the anti-tumor immune response by unleashing T cells against cancer cells. Understanding these mechanisms is crucial for predicting, preventing, and managing these AEs effectively.

### Excessive activation of the immune system

#### Aberrant activation of immune cells

Under normal conditions, the human immune system maintains self-tolerance mechanisms to prevent autoimmune responses. PD-1 inhibitors directly block the binding of PD-1 to its ligands PD-L1 and PD-L2, which completely deregulates the immunosuppressive signaling, strongly activates T cells, and enhances the anti-tumor immune response. However, over-activated T cells are more likely to mistakenly attack normal hematopoietic cells and cause hematologic toxicity. In contrast, PD-L1 inhibitors mainly block the binding of PD-L1 to PD-1 and B7.1, which not only activates T cells but also reduces the interference of immune activation with other immune regulatory mechanisms. This makes the immune activation triggered by PD-L1 inhibitors relatively milder, with a lower risk of attacking normal hematopoietic cells and a lower likelihood of hematological toxicity. However, while targeting tumor cells, activated T cells may mistakenly recognize normal hematopoietic cells in the peripheral blood or bone marrow as “non-self”. This disruption of immune tolerance can trigger immune-mediated attacks, impairing the production and survival of blood cells, ultimately leading to hematologic toxicities [47, 48]. Reports indicated that anemia occurred in approximately 5% of patients treated with ipilimumab and in less than 10% of those receiving anti-PD-1 agents. This anemia is frequently mediated by hemolysis or autoimmune mechanisms [3]. The immune environment altered by ICIs also impacts B cell activation, proliferation, and antibody secretion. Under the dysregulated influence of T cells and other immune cells, B cells may produce various autoantibodies targeting normal blood cells. This abnormal antibody production contributes to the development of immune-mediated hematologic toxicities [49]. When autoantibodies bind to blood cells, they often activate the complement system. This results in the formation of complement components, such as membrane attack complexes, which damage the cell membrane and other structures. These processes accelerate blood cell lysis and exacerbate hematologic toxicities. In autoimmune hemolytic anemia, complement activation directly disrupts red blood cell membranes, releasing their contents and resulting in anemia symptoms. PD-1 is often expressed on the surface of activated T-cells, B-cells, and NK-cells, while PD-L1 is often expressed on tumor cells, antigen-presenting cells, and so on. Therefore, the complement activation of PD-1 is stronger than that of PD-L1. Avelumab belongs to PD-L1, and this study also found that avelumab has the lowest risk of anemia, especially in the subgroup analysis of patients with gastroesophageal junction cancer.

#### Cytokine storm (CS)

Blocking immune checkpoints with ICIs activates the immune system, triggering immune cells like macrophages and natural killer cells to secrete large amounts of cytokines. Key cytokines, such as interleukins (IL-1, IL-6, IL-18), interferon-*γ* (IFN-*γ*), and tumor necrosis factor-*α* (TNF-*α*) play a central role in ICI-related immunopathology [50–53]. Excessive cytokine production leads to a CS [54]. The amount of cytokines secreted was proportional to CS and hematologic toxicities. Excessive cytokines can disrupt hematopoiesis by affecting hematopoietic Stem Cells (HSCs) and progenitor cells in the bone marrow, suppressing cell proliferation and differentiation, and reducing peripheral blood cell counts. IFN-*γ* and TNF-*α* inhibit erythropoiesis in vitro [55–57]. Lin et al. further discovered that IFN-*γ* and TNF-*α* can disrupt the production of common myeloid progenitors (CMPs). This disruption, along with the impaired proliferation of CMPs, granulocyte-monocyte progenitors, and megakaryocyte-erythroid progenitors, synergistically suppresses hematopoiesis. Ultimately, this leads to bone marrow aplasia and pancytopenia [58]. High IFN-*γ* levels inhibit erythroid progenitor differentiation, leading to anemia. PD-1 inhibitors act mainly through T-cell-mediated cytotoxicity, as their stronger T-cell activation secretes more cytokines that may lead to severe CS, resulting in a systemic inflammatory state throughout the body, causing changes in the survival environment of blood cells in the peripheral blood, accelerating the destruction and depletion of blood cells, such as causing neutrophils to over-aggregate at the site of inflammation and to be depleted, thus triggering peripheral blood neutropenia [59]. PD-L1 inhibitors secrete moderate amounts of IFN-*γ*, TNF-*α*, etc., and triggering a local inflammatory response is less hematologically toxic than PD-1 inhibitors.

#### Gut microbiota dysbiosis

Immune modulation is significantly influenced by the gut microbiome. Evidence suggests that its composition influences the host’s response to ICIs [60, 61]. Imbalances in the gut microbiota can exacerbate immune overactivation, worsening hematologic toxicities [44]. ICI treatment can alter microbiota composition, reducing beneficial bacteria and increasing potentially harmful ones [61]. For instance, immunoregulatory bacteria like bifidobacterium may decrease, while pro-inflammatory bacteria may increase [44]. Dysbiosis can impact immune function via pathways like the gut-bone marrow and gut-liver axes, disrupting HSC function and peripheral immune cell activity. This imbalance worsens blood cell damage and promotes hematologic toxicities [62]. Additionally, dysbiosis can affect the liver and other organs’ metabolism and transport of blood cell-related substances, such as iron and vitamins, impairing blood cell production and function [63].

The intensity of hematological toxicity caused by different ICIs affecting the intestinal flora varies. The antibody structure of PD-1 inhibitors makes their Fc segments more susceptible to binding to Fc receptors on the surface of immune cells in the intestinal tract, thereby activating the complement system or mediating antibody-dependent cell-mediated cytotoxicity. This process may directly damage intestinal epithelial cells and intestinal commensal microorganisms, disrupting the intestinal micro-ecological balance and causing haematological toxicity. PD-1 targets are mainly enriched in intestinal lamina propria T-cells. The antibody structure of the PD-1 inhibitor makes the Fc segment of the PD-1 inhibitor more susceptible to binding to Fc receptors on the surface of intestinal immune cells, which may mistakenly attack the gut epithelial cells through the strong activation of intestinal T-cells and other immune cells. This may result in damage to the intestinal barrier and increased permeability of the intestinal epithelium, giving endotoxins such as lipopolysaccharides the opportunity to enter the blood circulation and activate the immune system, triggering a systemic inflammatory response. Increased release of pro-inflammatory factors (IL-17, IFN-*γ*) disrupts the balance of flora, interferes with the bone marrow hematopoietic microenvironment, affects the normal function of hematopoietic stem cells, and ultimately triggers hematological toxicity, resulting in anemia, leukopenia, and other symptoms. PD-L1 is highly expressed in intestinal epithelial cells, macrophages, etc. The antibody structure of PD-L1 inhibitors has a relatively weak binding to Fc receptors, which is less disruptive to the intestinal microecology, and accordingly the risk of haematological toxicity is lower than that of PD-1 inhibitors.

#### Genetic factors

Genetic polymorphisms vary among patients, and certain genetic variations may increase susceptibility to immune ICIs, raising the risk of hematologic toxicities. For example, single nucleotide polymorphisms in genes related to immune regulation, apoptosis, and DNA damage repair have been linked to heightened vulnerability. These variations can lead to abnormal immune activation or impair tissue repair from immune attacks during immunotherapy, increasing the risk of hematologic toxicities [64]. Patients with genetic variations affecting HSC function or blood cell stability are more prone to these toxicities when treated with ICIs. Additionally, polymorphisms in HLA genes may influence immune response patterns, further affecting treatment outcomes [65].

#### Synergistic toxicity of combination therapy

HSCs are the origin of blood cell production and can differentiate into various blood cell types. ICIs are often combined with chemotherapy or targeted therapies, where overlapping mechanisms may directly or indirectly damage HSCs, exacerbating hematologic toxicities [66]. Chemotherapeutic agents like platinum-based drugs, paclitaxel, and cyclophosphamide can independently activate the immune system. When combined with ICIs, this dual activation may lead to excessive immune cell proliferation, which can attack HSCs, suppress bone marrow hematopoiesis, and reduce blood cell production, increasing hematologic toxicities [67]. Some targeted therapies, when combined with ICIs, may disrupt the HSC microenvironment, impairing HSC proliferation and differentiation, further contributing to these toxicities [68]. Certain biologics combined with ICIs can elevate inflammatory mediators such as TNF-*α* and IL-6, disrupting hematopoiesis in the bone marrow [69]. This inflammatory environment suppresses hematopoietic cells, reduces blood cell production, and accelerates blood cell destruction, worsening hematologic toxicities. A summary of the potential mechanisms for hematologic toxicities induced by ICIs is shown in Table S4.

## Limitations

This study has several limitations. First, the variation in drug doses among the included trials was minimal. Consequently, this study did not explore differences in hematologic toxicities across varying doses of the same drug. Second, 12 of the 25 trials included in this analysis used an open-label design, which may have introduced ascertainment bias. Third, the heterogeneity among the included studies—an inherent limitation of NMA—was also present in this study. Through subgroup analysis and meta regression, we found that ICI regimen may be responsible for the high heterogeneity observed in some of the results of this study.

## Conclusion

Safety is a critical factor in drug evaluation. This study conducted a comprehensive assessment of the hematologic toxicities of ICIs in digestive system tumors through a systematic review and NMA of multiple RCTs. This study identified variations in hematologic toxicities across different drugs and treatment combinations, considering cancer types and study phases. Each treatment regimen exhibited unique safety profiles. Compared with single ICI or nivolumab plus ipilimumab, chemotherapy with/without placebo or one or two ICIs with chemotherapy was associated with more severe hematologic toxicities. FN primarily presented as grade 3–5 events. Therefore, careful attention should be given to individual ICI drugs. In clinical practice, early detection and treatment of hematologic toxicities associated with ICIs are crucial. For mild (Grade 1–2) hematologic toxicity, routine blood tests should be performed every 2 weeks for the first 2 months after the start of ICIs, and every 3–4 weeks thereafter if 2 consecutive blood tests are stable and there are no signs of worsening hematologic toxicity. For moderate hematologic toxicity (Grade 3), routine blood tests should be performed once a week and changes in blood parameters should be closely monitored. For severe hematologic toxicity (Grade 4–5), routine blood tests should be performed every day or every other day to keep track of changes in blood parameters. Treatment with granulocyte colony-stimulating factor (G-CSF) is recommended for patients presenting with FN, severe neutropenia, and neutrophil count decreased. Dose is a key factor affecting toxicity, and different doses of ICIs cause differences in the degree of immune system activation. However, the relationship between the dose of ICIs and hematologic toxicity is still unclear, and there is often a lack of a precise basis for guiding the choice of dose of ICIs in clinical practice. Therefore, we strongly urge that in future clinical practice, physicians should pay great attention to the issue of dose adjustment of ICIs, conduct more prospective dose-exploratory clinical trials, pay attention to the issue of dose optimization in combination therapies, establish real-world research databases, and explore biomarkers related to the hematological toxicity of ICIs for early prediction and diagnosis by using modern precision medicine techniques. Additionally, patient education must be strengthened to improve awareness and management of ICIs-related hematologic toxicities among both patients and healthcare professionals.

## Supplementary Information

Below is the link to the electronic supplementary material.

Supplementary file1 (DOCX 9315 KB)

## Data Availability

No datasets were generated or analysed during the current study.

## Abbreviations

5-FU: 5-Fuorouracil
AE: Adverse event
AVE: Avelumab
CAM: Camrelizuma
CAP: Capecitabin
CIS: Cisplatin
CTCAE: Common terminology criteria for adverse events
DOC: Docetaxel
FLU: Fluorouracil
FN: Febrile neutropenia
GEM: Gemcitabine
ICIs: Immune checkpoint inhibitors
IPI: Ipilimumab
irAE: Immune-related adverse event
IRI: Irinotecan
MN: Multinational
NIV: Nivolumab
NMA: Network meta-analysis
OXA: Oxaliplatin
PAC: Paclitaxel
PD-1: Programmed death receptor 1
PEM: Pembrolizumab
PRISMA: Preferred reporting items for systematic reviews and meta-analysis
RCT: Randomized controlled trial
REL: Relatlimab
ROB: Risk of bias
SER: Serplulimab
SIN: Sintilimab
SOC: Socazolimab
SUCRA: Surface under the cumulative probability ranking curve
TIS: Tislelizumab
TOR: Toripalimab
WBC: White blood cell
