# Supplementary material for: Haematological toxicities with immune checkpoint inhibitors in digestive system tumors: a systematic review and network meta-analysis of randomized controlled trials
Source: Clin Exp Med. 2025 May 13;25(1):157. doi: 10.1007/s10238-025-01688-x (PMC12075026; doi:10.1007/s10238-025-01688-x)
Supplement: Supplementary file 1 — Supplementary file1 (DOCX 9315 KB) [file 10238_2025_1688_MOESM1_ESM.docx]

Supplementary Material

**Haematological toxicities with immune checkpoint inhibitors in digestive system tumors: a systematic review and network meta-analysis of randomized controlled trials**

Xinpu Han^1,2†^, Jing Xu^3†^, Meichen Cui1^1,2†^, Zhangjun Yun^1,2^, Hongbin Zhao^1,2^, Shaodan Tian^1^, Suicai Mi^4*^, Li Hou^1*^

^1^Department of Oncology and Hematology, Dongzhimen Hospital, Beijing University of Chinese Medicine, Beijing, China

^2^Beijing University of Chinese Medicine, China

^3^Hubei Provincial Hospital of Traditional Chinese Medicine, Hubei, China

^4^Xiamen Hospital of Traditional Chinese Medicine, Xiamen, China

*** Correspondence:**

Li Hou and Suicai Mi contributed equally to this work and share correspondence

Li Hou：[houli1203@126.com](mailto:houli1203@126.com)

Suicai Mi: [mihecai123@163.com](mailto:mihecai123@163.com)

^†^These authors contributed equally to this work.

**Supplementary Table 1.** Checklist of the PRISMA extension for network meta-analysis.

| **Section/Topic** | **Item #** | **Checklist Item** | **Reported on Page #** |
| --- | --- | --- | --- |
| **TITLE** |  |  |  |
| Title | 1 | Identify the report as a systematic review incorporating a network meta-analysis (or related form of meta-analysis). | **1** |
|  |  |  |  |
| **ABSTRACT** |  |  |  |
| Structured summary | 2 | Provide a structured summary including, as applicable:  **Background:** main objectives  **Methods:** data sources; study eligibility criteria, participants, and interventions; study appraisal; and synthesis methods, such as network meta-analysis.  **Results:** number of studies and participants identified; summary estimates with corresponding confidence/credible intervals; treatment rankings may also be discussed. Authors may choose to summarize pairwise comparisons against a chosen treatment included in their analyses for brevity.  **Discussion/Conclusions:** limitations; conclusions and implications of findings.  **Other:** primary source of funding; systematic review registration number with registry name. | 2-3 |
|  |  |  |  |
| **INTRODUCTION** |  |  |  |
| Rationale | 3 | Describe the rationale for the review in the context of what is already known, including mention of why a network meta-analysis has been conducted. | **3-4** |
| Objectives | 4 | Provide an explicit statement of questions being addressed, with reference to participants, interventions, comparisons, outcomes, and study design (PICOS). | 3-4 |
|  |  |  |  |
| **METHODS** |  |  |  |
| Protocol and registration | 5 | Indicate whether a review protocol exists and if and where it can be accessed (e.g., Web address); and, if available, provide registration information, including registration number. | 5 |
| Eligibility criteria | 6 | Specify study characteristics (e.g., PICOS, length of follow-up) and report characteristics (e.g., years considered, language, publication status) used as criteria for eligibility, giving rationale. Clearly describe eligible treatments included in the treatment network, and note whether any have been clustered or merged into the same node (with justification). | **5** |
| Information sources | 7 | Describe all information sources (e.g., databases with dates of coverage, contact with study authors to identify additional studies) in the search and date last searched. | 5 |
| Search | 8 | Present full electronic search strategy for at least one database, including any limits used, such that it could be repeated. | Supplementary table S2 |
| Study selection | 9 | State the process for selecting studies (i.e., screening, eligibility, included in systematic review, and, if applicable, included in the meta-analysis). | Figure 1 |
| Data collection process | 10 | Describe method of data extraction from reports (e.g., piloted forms, independently, in duplicate) and any processes for obtaining and confirming data from investigators. | 5-6 |
| Data items | 11 | List and define all variables for which data were sought (e.g., PICOS, funding sources) and any assumptions and simplifications made. | 5-6 |
| **Geometry of the network** | **S1** | Describe methods used to explore the geometry of the treatment network under study and potential biases related to it. This should include how the evidence base has been graphically summarized for presentation, and what characteristics were compiled and used to describe the evidence base to readers. | **5-6** |
| Risk of bias within individual studies | 12 | Describe methods used for assessing risk of bias of individual studies (including specification of whether this was done at the study or outcome level), and how this information is to be used in any data synthesis. | 6 |
| Summary measures | 13 | State the principal summary measures (e.g., risk ratio, difference in means). Also describe the use of additional summary measures assessed, such as treatment rankings and surface under the cumulative ranking curve (SUCRA) values, as well as modified approaches used to present summary findings from meta-analyses. | 6-7 |
| Planned methods of analysis | 14 | Describe the methods of handling data and combining results of studies for each network meta-analysis. This should include, but not be limited to:   - Handling of multi-arm trials; - Selection of variance structure; - Selection of prior distributions in Bayesian analyses; and - Assessment of model fit. | 6-7 |
| **Assessment of Inconsistency** | **S2** | Describe the statistical methods used to evaluate the agreement of direct and indirect evidence in the treatment network(s) studied. Describe efforts taken to address its presence when found. | 6-7 |
| Risk of bias across studies | 15 | Specify any assessment of risk of bias that may affect the cumulative evidence (e.g., publication bias, selective reporting within studies). | **6-7** |
| Additional analyses | 16 | Describe methods of additional analyses if done, indicating which were pre-specified. This may include, but not be limited to, the following:   - Sensitivity or subgroup analyses; - Meta-regression analyses; - Alternative formulations of the treatment network; and - Use of alternative prior distributions for Bayesian analyses (if applicable). | **6-8** |
| **RESULTS†** |  |  |  |
| Study selection | 17 | Give numbers of studies screened, assessed for eligibility, and included in the review, with reasons for exclusions at each stage, ideally with a flow diagram. | Figure 1 |
| **Presentation of network structure** | **S3** | Provide a network graph of the included studies to enable visualization of the geometry of the treatment network. | **Figure 2** |
| **Summary of network geometry** | **S4** | Provide a brief overview of characteristics of the treatment network. This may include commentary on the abundance of trials and randomized patients for the different interventions and pairwise comparisons in the network, gaps of evidence in the treatment network, and potential biases reflected by the network structure. | **8** |
| Study characteristics | 18 | For each study, present characteristics for which data were extracted (e.g., study size, PICOS, follow-up period) and provide the citations. | Table 1 |
| Risk of bias within studies | 19 | Present data on risk of bias of each study and, if available, any outcome level assessment. | 9 |
| Results of individual studies | 20 | For all outcomes considered (benefits or harms), present, for each study: 1) simple summary data for each intervention group, and 2) effect estimates and confidence intervals. Modified approaches may be needed to deal with information from larger networks. | 9 |
| Synthesis of results | 21 | Present results of each meta-analysis done, including confidence/credible intervals. In larger networks, authors may focus on comparisons versus a particular comparator (e.g. placebo or standard care), with full findings presented in an appendix. League tables and forest plots may be considered to summarize pairwise comparisons. If additional summary measures were explored (such as treatment rankings), these should also be presented. | **9-15**  **Figure 4, Supplementary** **figure S1-S8** |
| **Exploration for inconsistency** | **S5** | Describe results from investigations of inconsistency. This may include such information as measures of model fit to compare consistency and inconsistency models, P values from statistical tests, or summary of inconsistency estimates from different parts of the treatment network. | **15, Supplementary table S3** |
| Risk of bias across studies | 22 | Present results of any assessment of risk of bias across studies for the evidence base being studied. | 9, Figure 5 |
| Results of additional analyses | 23 | Give results of additional analyses, if done (e.g., sensitivity or subgroup analyses, meta-regression analyses, alternative network geometries studied, alternative choice of prior distributions for Bayesian analyses, and so forth). | **15-17, Supplementary** **figure S9-S26** |
|  |  |  |  |
| **DISCUSSION** |  |  |  |
| Summary of evidence | 24 | Summarize the main findings, including the strength of evidence for each main outcome; consider their relevance to key groups (e.g., healthcare providers, users, and policy-makers). | 17-24 |
| Limitations | 25 | Discuss limitations at study and outcome level (e.g., risk of bias), and at review level (e.g., incomplete retrieval of identified research, reporting bias). Comment on the validity of the assumptions, such as transitivity and consistency. Comment on any concerns regarding network geometry (e.g., avoidance of certain comparisons). | 24-25 |
| Conclusions | 26 | Provide a general interpretation of the results in the context of other evidence, and implications for future research. | 25-26 |
|  |  |  |  |
| **FUNDING** |  |  |  |
| Funding | 27 | Describe sources of funding for the systematic review and other support (e.g., supply of data); role of funders for the systematic review. This should also include information regarding whether funding has been received from manufacturers of treatments in the network and/or whether some of the authors are content experts with professional conflicts of interest that could affect use of treatments in the network. | **27** |
|  |  |  |  |

PICOS = population, intervention, comparators, outcomes, study design.

* Text in italics indicates wording specific to reporting of network meta-analyses that has been added to guidance from the PRISMA statement.

† Authors may wish to plan for use of appendices to present all relevant information in full detail for items in this section.

**Supplementary Table 2.** Studies search criteria.

**Supplementary Table 2-1.** Pubmed, from database inception to August 8, 2024.

| Search number | Query |
| --- | --- |
| 6 | 1 AND 4 AND 5 |
| 5 | ((((((randomized controlled trial[Title/Abstract]) OR (controlled clinical trial)) OR (randomized)) OR (placebo)) OR (clinical trials as topic)) OR (randomly)) OR (trial) |
| 4 | 2 OR 3 |
| 3 | ((((((((((((((((Immune checkpoint inhibitor[Title/Abstract]) OR (Ipilimumab)) OR (Nivolumab)) OR (Pembrolizumab)) OR (Sintilimab)) OR (Tislelizumab)) OR (Pidilizumab)) OR (Atezolizumab)) OR (Avelumab)) OR (Camrelizumab)) OR (Serplulimab)) OR (Socazolimab)) OR (Tislelizumab)) OR (Toripalimab)) OR (CTLA-4) OR (PD-1) OR (PD-L1) |
| 2 | ((((((((((((((((((((((((((((((((Primary Myelofibrosis[MeSH Terms]) OR (Anemias)) OR (Neutropenia)) OR (Neutropenias)) OR ([Cytopeni](https://www.ncbi.nlm.nih.gov/mesh/2106396)a)) OR (Thrombocytopenia)) OR (thrombocytopenia)) OR (Leukopenia)) OR (Abnormal hematopoiesis)) OR (Anaemia)) OR (Anemia)) OR (lymphadenopathy)) OR (Hemorrhage)) OR (Hematologic toxicity)) OR (Bone Marrow suppression)) OR (Thrombocytopenias)) OR (Thrombopenia)) OR (Thrombopenias)) OR (Bone Marrow Fibrosis)) OR (Myelofibrosis)) OR (Myelosclerosis)) OR (Myeloscleroses)) OR (Nonleukemic Myeloses)) OR (Nonleukemic Myelosis)) OR (Chronic Idiopathic Myelofibrosis)) OR (Idiopathic Myelofibrosis)) OR (Hepatosplenomegaly)) OR (Lymphadenopathies)) OR (Adenopathy)) OR (Adenopathies)) OR (bleeding)) OR (Bloodletting)) OR (Phlebotomy) |
| 1 | (((((((((((((((((((((((((((((((((((((((((((((((((((((((((((((((((((((Digestive System Neoplasms[MeSH Terms]) OR (Gastrointestinal Neoplasms)) OR (Digestive System Neoplasm)) OR (Cancer of Digestive System)) OR (Digestive System Cancers)) OR (Cancer of the Digestive System)) OR (Digestive System Cancers)) OR (Stomach Neoplasm)) OR (Cancer of Stomach)) OR (Stomach Cancers)) OR (Cancer of the Stomach)) OR (Stomach Cancers)) OR (Cancer of the Stomach)) OR (Gastric Cancer)) OR (Stomach Cancer)) OR ([Stomach Neoplasms](https://www.ncbi.nlm.nih.gov/mesh/68013274))) OR (Gastrointestinal Neoplasm)) OR (Intestinal Neoplasms)) OR (Intestines Neoplasms)) OR (Intestines Cancers)) OR (Intestinal Cancers)) OR (Cancer of the Intestines)) OR (Intestines Cancer)) OR (Cancer of Gastrointestinal Tract)) OR (Gastrointestinal Cancer)) OR (Esophageal cancer)) OR (Esophageal Neoplasm)) OR (Esophagus Neoplasm)) OR (Cancer of Esophagus)) OR (Esophageal Cancer)) OR (Cancer of the Esophagus)) OR (Esophagus Cancers)) OR (Colonic Neoplasm)) OR (Neoplasm, Colonic)) OR (Neoplasms, Colonic)) OR (Colon Neoplasms)) OR (Colon Neoplasm)) OR (Neoplasm, Colon)) OR (Neoplasms, Colon)) OR (Cancer of Colon)) OR (Colon Cancers)) OR (Colon Cancer)) OR (Cancer, Colon)) OR (Cancers, Colon)) OR (Cancer of the Colon)) OR (Colonic Cancer)) OR (Cancer, Colonic)) OR (Cancers, Colonic)) OR (Colonic Cancers)) OR (Colon Adenocarcinoma)) OR (Adenocarcinoma, Colon)) OR (Adenocarcinomas, Colon)) OR (Colon Adenocarcinomas)) OR (Hepatic Neoplasm)) OR (Hepatic Neoplasm)) OR (Neoplasm, Hepatic)) OR (Neoplasms, Hepatic)) OR (Neoplasms, Liver)) OR (Hepatic Cancers)) OR (Hepatocellular Cancer) OR (Liver Cancer) OR (Pancreatic Neoplasm) OR (Pancreas Neoplasms) OR (Pancreas Cancers) OR (Pancreas Cancer) OR (Pancreatic Cancer) OR (Pancreatic Carcinoma) OR (Pancreatic Carcinomas) OR (Pancreatic Acinar Carcinoma) OR (Pancreatic Acinar Carcinomas) OR (Biliary Tract Neoplasm) OR (Biliary Tract Cancer) OR (Biliary Tract Cancers) OR (Cancer of the Biliary Tract) OR (Cancer of Biliary Tract) OR (Rectal Neoplasms) OR (Rectum Neoplasms)) OR (Rectal Tumors)) OR (Cancer of Rectum)) OR (Rectum Cancers)) OR (Cancer of the Rectum)) OR (Rectal Cancer)) OR (Rectum Cancer)) OR (Anus Neoplasms)) OR (Anus Neoplasm)) OR (Anal Neoplasms)) OR (Anal Cancer) OR (Cancer of Anus) OR (Anus Cancer) OR (Cancer of the Anus) |

**Supplementary Table 2-2.** Embase, from database inception to August 8, 2024.

| No. | Query Results |
| --- | --- |
| #6. | #1 AND #4 AND #5 |
| #5. | randomized controlled trial:ti,ab,kw OR controlled clinical trial:ti,ab,kw OR randomized:ti,ab,kw OR placebo:ti,ab,kw OR clinical trials as topic:ti,ab,kw OR randomly:ti,ab,kw OR trial:ti,ab,kw |
| #4. | #2 OR #3 |
| #3. | Immunotherapies:ti,ab,kw OR Ipilimumab:ti,ab,kw OR Nivolumab:ti,ab,kw OR Pembrolizumab:ti,ab,kw OR Sintilimab:ti,ab,kw OR Tislelizumab:ti,ab,kw OR Pidilizumab: ti,ab,kw OR Atezolizumab:ti,ab,kw OR Avelumab:ti,ab,kw OR Camrelizumab:ti,ab,kw OR Serplulimab:ti,ab,kw OR Socazolimab:ti,ab,kw OR Tislelizumab:ti,ab,kw OR Toripalimab: ti,ab,kw OR CTLA-4:ti,ab,kw OR PD-1:ti,ab,kw OR PD-L1:ti,ab,kw |
| #2. | Primary Myelofibrosis:ti,ab,kw OR Anemias:ti,ab,kw OR Neutropenia:ti,ab,kw OR Neutropenias:ti,ab,kw OR [Cytopeni](https://www.ncbi.nlm.nih.gov/mesh/2106396)a:ti,ab,kw OR Thrombocytopenia:ti,ab,kw OR Leukopenia:ti,ab,kw OR Abnormal hematopoiesis:ti,ab,kw OR Anemia:ti,ab,kw OR lymphadenopathy:ti,ab,kw OR Hemorrhage:ti,ab,kw OR Hematologic toxicity:ti,ab,kw OR Bone Marrow suppression:ti,ab,kw OR Thrombocytopenias:ti,ab,kw OR Thrombopenia:ti,ab,kw OR Thrombopenias:ti,ab,kw OR Bone Marrow Fibrosis:ti,ab,kw OR Myelofibrosis:ti,ab,kw OR Myelosclerosis:ti,ab,kw OR Myeloscleroses:ti,ab,kw OR Nonleukemic Myeloses:ti,ab,kw OR Nonleukemic Myelosis:ti,ab,kw OR Chronic Idiopathic Myelofibrosis:ti,ab,kw OR Idiopathic Myelofibrosis:ti,ab,kw OR Hepatosplenomegaly:ti,ab,kw OR Lymphadenopathies:ti,ab,kw OR Adenopathy:ti,ab,kw OR Adenopathies:ti,ab,kw OR bleeding:ti,ab,kw OR Bloodletting:ti,ab,kw OR Phlebotomy:ti,ab,kw |
| #1. | Digestive System Neoplasms:ti,ab,kw OR Gastrointestinal Neoplasms:ti,ab,kw OR Digestive System Neoplasm:ti,ab,kw OR Cancer of Digestive System:ti,ab,kw OR Digestive System Cancers:ti,ab,kw OR Cancer of the Digestive System:ti,ab,kw OR Digestive System Cancers:ti,ab,kw OR Stomach Neoplasm:ti,ab,kw OR Cancer of Stomach:ti,ab,kw OR Stomach Cancers:ti,ab,kw OR Cancer of the Stomach:ti,ab,kw OR Stomach Cancers:ti,ab,kw OR Cancer of the Stomach:ti,ab,kw OR Gastric Cancer:ti,ab,kw OR Stomach Cancer:ti,ab,kw OR [Stomach Neoplasms](https://www.ncbi.nlm.nih.gov/mesh/68013274):ti,ab,kw OR Gastrointestinal Neoplasm:ti,ab,kw OR Intestinal Neoplasms:ti,ab,kw OR Intestines Neoplasms:ti,ab,kw OR Intestines Cancers:ti,ab,kw OR Intestinal Cancers:ti,ab,kw OR Cancer of the Intestines:ti,ab,kw OR Intestines Cancer:ti,ab,kw OR Cancer of Gastrointestinal Tract:ti,ab,kw OR Gastrointestinal Cancer:ti,ab,kw OR Esophageal cancer:ti,ab,kw OR Esophageal Neoplasm:ti,ab,kw OR Esophagus Neoplasm:ti,ab,kw OR Cancer of Esophagus:ti,ab,kw OR Esophageal Cancer:ti,ab,kw OR Cancer of the Esophagus:ti,ab,kw OR Esophagus Cancers:ti,ab,kw OR Colonic Neoplasm:ti,ab,kw OR Neoplasm, Colonic:ti,ab,kw OR Neoplasms, Colonic:ti,ab,kw OR Colon Neoplasms:ti,ab,kw OR Colon Neoplasm:ti,ab,kw OR Neoplasm, Colon:ti,ab,kw OR Neoplasms, Colon:ti,ab,kw OR Cancer of Colon:ti,ab,kw OR Colon Cancers:ti,ab,kw OR Colon Cancer:ti,ab,kw OR Cancer, Colon:ti,ab,kw OR Cancers, Colon:ti,ab,kw OR Cancer of the Colon:ti,ab,kw OR Colonic Cancer:ti,ab,kw OR Cancer, Colonic:ti,ab,kw OR Cancers, Colonic:ti,ab,kw OR Colonic Cancers:ti,ab,kw OR Colon Adenocarcinoma:ti,ab,kw OR Adenocarcinoma, Colon:ti,ab,kw OR Adenocarcinomas, Colon:ti,ab,kw OR Colon Adenocarcinomas:ti,ab,kw OR Hepatic Neoplasm:ti,ab,kw OR Hepatic Neoplasm:ti,ab,kw OR Neoplasm, Hepatic:ti,ab,kw OR Neoplasms, Hepatic:ti,ab,kw OR Neoplasms, Liver:ti,ab,kw OR Hepatic Cancers:ti,ab,kw OR Hepatocellular Cancer:ti,ab,kw OR Liver Cancer:ti,ab,kw OR Pancreatic Neoplasm:ti,ab,kw OR Pancreas Neoplasms:ti,ab,kw OR Pancreas Cancers:ti,ab,kw OR Pancreas Cancer:ti,ab,kw OR Pancreatic Cancer:ti,ab,kw OR Pancreatic Carcinoma:ti,ab,kw OR Pancreatic Carcinomas:ti,ab,kw OR Pancreatic Acinar Carcinoma:ti,ab,kw OR Pancreatic Acinar Carcinomas:ti,ab,kw OR Biliary Tract Neoplasm:ti,ab,kw OR Biliary Tract Cancer:ti,ab,kw OR Biliary Tract Cancers:ti,ab,kw OR Cancer of the Biliary Tract:ti,ab,kw OR Cancer of Biliary Tract:ti,ab,kw OR Rectal Neoplasms:ti,ab,kw OR Rectum Neoplasms:ti,ab,kw OR Rectal Tumors:ti,ab,kw OR Cancer of Rectum:ti,ab,kw OR Rectum Cancers:ti,ab,kw OR Cancer of the Rectum:ti,ab,kw OR Rectal Cancer:ti,ab,kw OR Rectum Cancer:ti,ab,kw OR Anus Neoplasms:ti,ab,kw OR Anus Neoplasm:ti,ab,kw OR Anal Neoplasms:ti,ab,kw OR Anal Cancer:ti,ab,kw OR Cancer of Anus:ti,ab,kw OR Anus Cancer:ti,ab,kw OR Cancer of the Anus:ti,ab,kw |

**Supplementary Table 2-3.** COCHRANE, from database inception to August 8, 2024.

| No. | Query Results |
| --- | --- |
| #6. | #1 AND #4 AND #5 |
| #5. | randomized controlled trial OR controlled clinical trial:ti,ab,kw OR randomized:ti,ab,kw OR placebo:ti,ab,kw OR clinical trials as topic:ti,ab,kw OR randomly:ti,ab,kw OR trial:ti,ab,kw |
| #4. | #2 OR #3 |
| #3. | Immunotherapies:ti,ab,kw OR Ipilimumab:ti,ab,kw OR Nivolumab:ti,ab,kw OR Pembrolizumab:ti,ab,kw OR Sintilimab:ti,ab,kw OR Tislelizumab:ti,ab,kw OR Pidilizumab: ti,ab,kw OR Atezolizumab:ti,ab,kw OR Avelumab:ti,ab,kw OR Camrelizumab:ti,ab,kw OR Serplulimab:ti,ab,kw OR Socazolimab:ti,ab,kw OR Tislelizumab:ti,ab,kw OR Toripalimab: ti,ab,kw OR CTLA-4:ti,ab,kw OR PD-1:ti,ab,kw OR PD-L1:ti,ab,kw |
| #2. | Primary Myelofibrosis:ti,ab,kw OR Anemias:ti,ab,kw OR Neutropenia:ti,ab,kw OR Neutropenias:ti,ab,kw OR [Cytopeni](https://www.ncbi.nlm.nih.gov/mesh/2106396)a:ti,ab,kw OR Thrombocytopenia:ti,ab,kw OR Leukopenia:ti,ab,kw OR Abnormal hematopoiesis:ti,ab,kw OR Anemia:ti,ab,kw OR lymphadenopathy:ti,ab,kw OR Hemorrhage:ti,ab,kw OR Hematologic toxicity:ti,ab,kw OR Bone Marrow suppression:ti,ab,kw OR Thrombocytopenias:ti,ab,kw OR Thrombopenia:ti,ab,kw OR Thrombopenias:ti,ab,kw OR Bone Marrow Fibrosis:ti,ab,kw OR Myelofibrosis:ti,ab,kw OR Myelosclerosis:ti,ab,kw OR Myeloscleroses:ti,ab,kw OR Nonleukemic Myeloses:ti,ab,kw OR Nonleukemic Myelosis:ti,ab,kw OR Chronic Idiopathic Myelofibrosis:ti,ab,kw OR Idiopathic Myelofibrosis:ti,ab,kw OR Hepatosplenomegaly:ti,ab,kw OR Lymphadenopathies:ti,ab,kw OR Adenopathy:ti,ab,kw OR Adenopathies:ti,ab,kw OR bleeding:ti,ab,kw OR Bloodletting:ti,ab,kw OR Phlebotomy:ti,ab,kw |
| #1. | Digestive System Neoplasms:ti,ab,kw OR Gastrointestinal Neoplasms:ti,ab,kw OR Digestive System Neoplasm:ti,ab,kw OR Cancer of Digestive System:ti,ab,kw OR Digestive System Cancers:ti,ab,kw OR Cancer of the Digestive System:ti,ab,kw OR Digestive System Cancers:ti,ab,kw OR Stomach Neoplasm:ti,ab,kw OR Cancer of Stomach:ti,ab,kw OR Stomach Cancers:ti,ab,kw OR Cancer of the Stomach:ti,ab,kw OR Stomach Cancers:ti,ab,kw OR Cancer of the Stomach:ti,ab,kw OR Gastric Cancer:ti,ab,kw OR Stomach Cancer:ti,ab,kw OR [Stomach Neoplasms](https://www.ncbi.nlm.nih.gov/mesh/68013274):ti,ab,kw OR Gastrointestinal Neoplasm:ti,ab,kw OR Intestinal Neoplasms:ti,ab,kw OR Intestines Neoplasms:ti,ab,kw OR Intestines Cancers:ti,ab,kw OR Intestinal Cancers:ti,ab,kw OR Cancer of the Intestines:ti,ab,kw OR Intestines Cancer:ti,ab,kw OR Cancer of Gastrointestinal Tract:ti,ab,kw OR Gastrointestinal Cancer:ti,ab,kw OR Esophageal cancer:ti,ab,kw OR Esophageal Neoplasm:ti,ab,kw OR Esophagus Neoplasm:ti,ab,kw OR Cancer of Esophagus:ti,ab,kw OR Esophageal Cancer:ti,ab,kw OR Cancer of the Esophagus:ti,ab,kw OR Esophagus Cancers:ti,ab,kw OR Colonic Neoplasm:ti,ab,kw OR Neoplasm, Colonic:ti,ab,kw OR Neoplasms, Colonic:ti,ab,kw OR Colon Neoplasms:ti,ab,kw OR Colon Neoplasm:ti,ab,kw OR Neoplasm, Colon:ti,ab,kw OR Neoplasms, Colon:ti,ab,kw OR Cancer of Colon:ti,ab,kw OR Colon Cancers:ti,ab,kw OR Colon Cancer:ti,ab,kw OR Cancer, Colon:ti,ab,kw OR Cancers, Colon:ti,ab,kw OR Cancer of the Colon:ti,ab,kw OR Colonic Cancer:ti,ab,kw OR Cancer, Colonic:ti,ab,kw OR Cancers, Colonic:ti,ab,kw OR Colonic Cancers:ti,ab,kw OR Colon Adenocarcinoma:ti,ab,kw OR Adenocarcinoma, Colon:ti,ab,kw OR Adenocarcinomas, Colon:ti,ab,kw OR Colon Adenocarcinomas:ti,ab,kw OR Hepatic Neoplasm:ti,ab,kw OR Hepatic Neoplasm:ti,ab,kw OR Neoplasm, Hepatic:ti,ab,kw OR Neoplasms, Hepatic:ti,ab,kw OR Neoplasms, Liver:ti,ab,kw OR Hepatic Cancers:ti,ab,kw OR Hepatocellular Cancer:ti,ab,kw OR Liver Cancer:ti,ab,kw OR Pancreatic Neoplasm:ti,ab,kw OR Pancreas Neoplasms:ti,ab,kw OR Pancreas Cancers:ti,ab,kw OR Pancreas Cancer:ti,ab,kw OR Pancreatic Cancer:ti,ab,kw OR Pancreatic Carcinoma:ti,ab,kw OR Pancreatic Carcinomas:ti,ab,kw OR Pancreatic Acinar Carcinoma:ti,ab,kw OR Pancreatic Acinar Carcinomas:ti,ab,kw OR Biliary Tract Neoplasm:ti,ab,kw OR Biliary Tract Cancer:ti,ab,kw OR Biliary Tract Cancers:ti,ab,kw OR Cancer of the Biliary Tract:ti,ab,kw OR Cancer of Biliary Tract:ti,ab,kw OR Rectal Neoplasms:ti,ab,kw OR Rectum Neoplasms:ti,ab,kw OR Rectal Tumors:ti,ab,kw OR Cancer of Rectum:ti,ab,kw OR Rectum Cancers:ti,ab,kw OR Cancer of the Rectum:ti,ab,kw OR Rectal Cancer:ti,ab,kw OR Rectum Cancer:ti,ab,kw OR Anus Neoplasms:ti,ab,kw OR Anus Neoplasm:ti,ab,kw OR Anal Neoplasms:ti,ab,kw OR Anal Cancer:ti,ab,kw OR Cancer of Anus:ti,ab,kw OR Anus Cancer:ti,ab,kw OR Cancer of the Anus:ti,ab,kw |

**Supplementary Table 2-4.** Web of Science, from database inception to August 8, 2024.

| # | Query |
| --- | --- |
| 1 | TS=(Gastrointestinal Neoplasms OR Stomach Neoplasm OR Cancer of Stomach OR Stomach Cancers OR Cancer of the Stomach OR Stomach Cancers OR Cancer of the Stomach OR Gastric Cancer OR Stomach Cancer OR [Stomach Neoplasms](https://www.ncbi.nlm.nih.gov/mesh/68013274) OR Gastrointestinal Neoplasm OR Cancer of Gastrointestinal Tract OR Gastrointestinal Cancer OR Esophageal cancer OR Esophageal Neoplasm OR Esophagus Neoplasm OR Cancer of Esophagus OR Esophageal Cancer OR Cancer of the Esophagus OR Esophagus Cancers OR Colonic Neoplasm OR Colon Neoplasms OR Colon Neoplasm OR Cancer of Colon OR Colon Cancers OR Colon Cancer OR Cancer of the Colon OR Colonic Cancer OR Colonic Cancers OR Colon Adenocarcinoma OR Colon Adenocarcinomas OR Hepatic Neoplasm OR Hepatic Neoplasm OR Hepatic Cancers OR Hepatocellular Cancer OR Liver Cancer OR Pancreatic Neoplasm OR Pancreas Neoplasms OR Pancreas Cancers OR Pancreas Cancer OR Pancreatic Cancer OR Pancreatic Carcinoma OR Pancreatic Carcinomas OR Pancreatic Acinar Carcinoma OR Pancreatic Acinar Carcinomas OR Biliary Tract Neoplasm OR Biliary Tract Cancer OR Biliary Tract Cancers OR Cancer of the Biliary Tract OR Cancer of Biliary Tract OR Digestive System Neoplasms OR Intestinal Neoplasms OR Intestines Neoplasms OR Intestines Cancers OR Intestinal Cancers OR Cancer of the Intestines OR Intestines Cancer OR Rectal Neoplasms OR Rectum Neoplasms OR Rectal Tumors OR Cancer of Rectum OR Rectum Cancers OR Cancer of the Rectum OR Rectal Cancer OR Rectum Cancer OR Anus Neoplasms OR Anus Neoplasm OR Anal Neoplasms OR Anal Cancer OR Cancer of Anus OR Anus Cancer OR Cancer of the Anus) |
| 2 | TS=(Primary Myelofibrosis OR Anemias OR Neutropenia OR Neutropenias OR [Cytopeni](https://www.ncbi.nlm.nih.gov/mesh/2106396)a OR Thrombocytopenia OR Leukopenia OR Abnormal hematopoiesis OR Anemia OR Lymphadenopathy OR Hemorrhage OR Hematologic toxicityn OR Bone Marrow suppression OR Thrombocytopenias OR Thrombopenia OR Thrombopenias OR Bone Marrow Fibrosis OR Myelofibrosis OR Myelosclerosis OR Myeloscleroses OR Nonleukemic Myeloses OR Nonleukemic Myelosis OR Chronic Idiopathic Myelofibrosis OR Idiopathic Myelofibrosis OR Hepatosplenomegaly OR Lymphadenopathies OR Adenopathy OR Adenopathies OR bleeding OR Bloodletting OR Phlebotomy) |
| 3 | TS=(Immunotherapies OR Ipilimumab OR Nivolumab OR Pembrolizumab OR Sintilimab OR Tislelizumab OR Pidilizumab OR Atezolizumab OR Avelumab OR Camrelizumab OR Serplulimab OR Socazolimab OR Tislelizumab OR Toripalimab OR CTLA-4 OR PD-1 OR PD-L1) |
| 4 | #2 OR #3 |
| 5 | TS=(randomized controlled trial OR controlled clinical trial OR randomized OR placebo OR clinical trials as topic OR randomly OR trial) |
| 6 | #1 AND #4 AND #5 |

**Supplementary Table 3.** The P value results of inconsistency model analysis and the heterogeneity in network meta-analysis.

| Intervention | Grade 1-5 | | Grade 3-5 | |
| --- | --- | --- | --- | --- |
|  | **Consistency (P value)** | **Heterogeneity (%)** | **Consistency (P value)** | **Heterogeneity (%)** |
| Anaemia | 0.7773 | 91.2 | 0.4606 | 72.9 |
| Neutropenia | 0.0583 | 83.3 | 0.4552 | 73.1 |
| Neutrophil count decreased | 0.7019 | 91.5 | 0.6088 | 77.5 |
| Thrombocytopenia | 0.7048 | 67.4 | 0.3169 | 29.5 |
| Platelet count decreased | 0.7328 | 62.9 | 0.2113 | 31.3 |
| Leukopenia | 0.9911 | 87.9 | 0.0463 | 54.6 |
| White blood cell count decreased | 0.6431 | 91.9 | 0.4247 | 74.2 |
| Lymphocyte count decreased | 0.7547 | 0 | 0.5497 | 0 |
| Febrile neutropenia | 0.5708 | 70.8 | 0.5708 | 70.8 |
| Esophageal cancer | 0.5132 | 93.4 | 0.1889 | 72.2 |
| Gastric or gastro-oesophageal junction cancer | 0.4642 | 89.1 | 0.0889 | 77.7 |

**Supplementary Table 4.** Summary of potential mechanisms for the occurrence of hematologic toxicities induced by ICIs.

| **Category** | | **Specific Mechanism** | **Outcome** | **References** | |
| --- | --- | --- | --- | --- | --- |
| Excessive activation of the immune system | Abnormal activation of immune cells | Abnormally activated T cells, while attacking tumor cells, mistakenly recognize normal hematopoietic cells in the bone marrow or peripheral blood as "non-self," breaking immune tolerance and initiating immune attacks. | Disruption of normal hematopoiesis, including production and survival of blood cells, leading to hematologic toxicity. | | [47,48] |
|  |  | Abnormally activated B cells produce various autoantibodies targeting self-blood cells. These autoantibodies bind to blood cells, often activating the complement system and forming membrane attack complexes. | Further damage to blood cell membranes, accelerating blood cell lysis and exacerbating hematologic toxicity. | | [49] |
|  | Cytokine Storm | Excessive cytokines directly affect hematopoietic stem cells and precursor cells in the bone marrow, interfering with their normal proliferation and differentiation processes. | Suppression of hematopoiesis, resulting in reduced blood cell counts in peripheral blood. | | [55,56,57,58] |
|  |  | Systemic inflammatory state alters the survival environment of blood cells in peripheral blood. | Accelerated destruction and consumption of blood cells. | | [59] |
| Gut microbiota dysbiosis | | Impairs absorption of essential nutrients such as iron, folic acid, and vitamin B12; disrupts the function of hematopoietic stem cells in the bone marrow; exacerbates immune system overactivation. | Indirectly worsens hematopoiesis and blood cell function maintenance, promoting the development of hematologic toxicity. | | [60,61,62,63] |
| Genetic factors | | Single nucleotide polymorphisms in genes related to immune regulation, apoptosis, and DNA damage repair; mutations in genes affecting hematopoietic stem cell function or blood cell stability; polymorphisms in HLA genes. | Increased risk of hematologic toxicity. | | [64,65] |
| Synergistic toxicity of combination therapy | | Double immune activation and increased inflammatory response lead to excessive proliferation and activation of immune cells, accelerating the destruction of blood cells. | Attack hematopoietic stem cells, inhibit normal bone marrow hematopoietic, reduce blood cell production, and further aggravate the occurrence and development of blood toxicity. | | [66,67,68,69] |

**
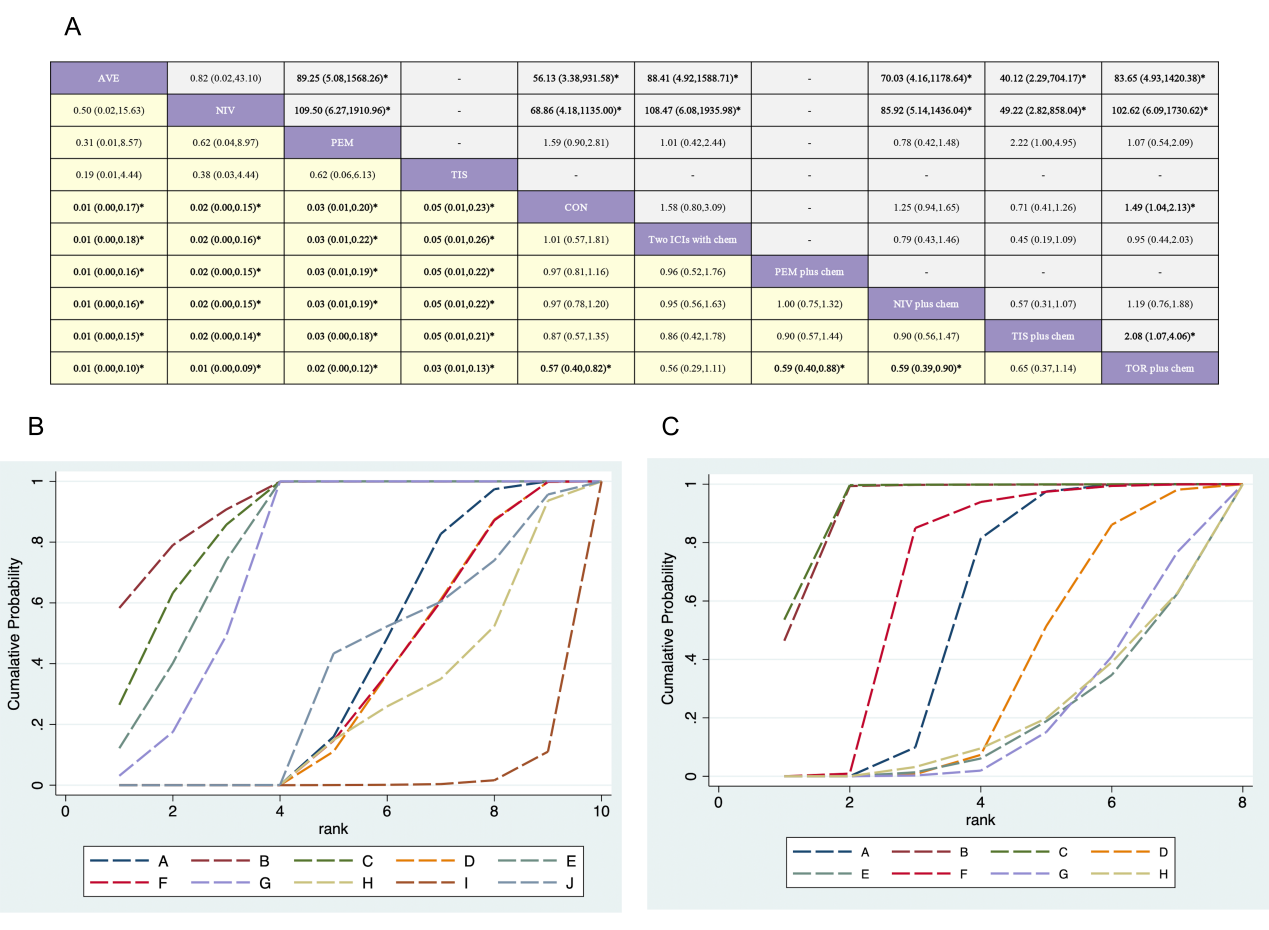
**

**Supplementary Figure 1.** Safety profile (A), ranking curves of grade 1-5 (B), ranking curves of grade 3-5 (C) according to the drug based network meta-analysis in neutropenia. ICI=immune checkpoint inhibitor.

* High certainty of evidence.

Abbreviations in Fig S1B: A = chemotherapy with/without placebo; B = avelumab; C = nivolumab; D = nivolumab plus ipilimumab; E = pembrolizumab; F = pembrolizumab plus chemotherapy; G = tislelizumab; H = tislelizumab plus chemotherapy; I = toripalimab plus chemotherapy; J = two ICI drugs with chemotherapy.

Abbreviations in Fig S1C: A = chemotherapy with/without placebo; B = avelumab; C = nivolumab; D = nivolumab plus ipilimumab; E = pembrolizumab; F = tislelizumab plus chemotherapy; G = toripalimab plus chemotherapy; H = two ICI drugs with chemotherapy.


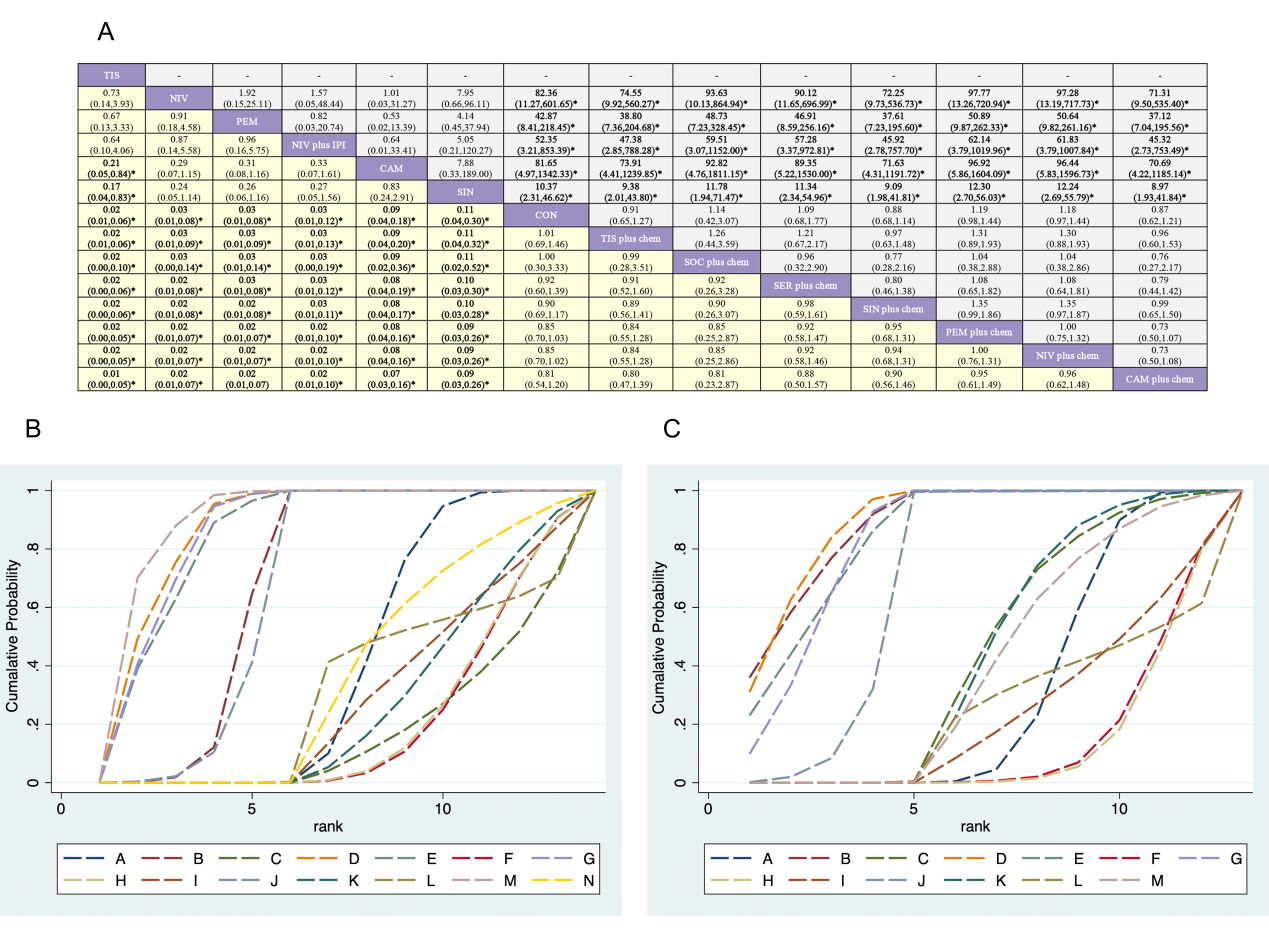


**Supplementary Figure 2.** Safety profile (A), ranking curves of grade 1-5 (B), ranking curves of grade 3-5 (C) according to the drug based network meta-analysis in neutrophil count decreased.

* High certainty of evidence.

Abbreviations in Fig S2B: A = chemotherapy with/without placebo; B = camrelizumab; C= camrelizumab plus chemotherapy; D= nivolumab; E = nivolumab plus ipilimumab; F = nivolumab plus chemotherapy; G = pembrolizumab; H = pembrolizumab plus chemotherapy; I = serplulimab plus chemotherapy; J = sintilimab; K = sintilimab plus chemotherapy; L = socazolimab plus chemotherapy; M = tislelizumab; N = tislelizumab plus chemotherapy.

Abbreviations in Fig S2C: A = chemotherapy with/without placebo; B = camrelizumab; C = camrelizumab plus chemotherapy; D = nivolumab; E = nivolumab plus ipilimumab; F = nivolumab plus chemotherapy; G = pembrolizumab; H = pembrolizumab plus chemotherapy; I = serplulimab plus chemotherapy; J = sintilimab; K = sintilimab plus chemotherapy; L = socazolimab plus chemotherapy; M = tislelizumab plus chemotherapy.


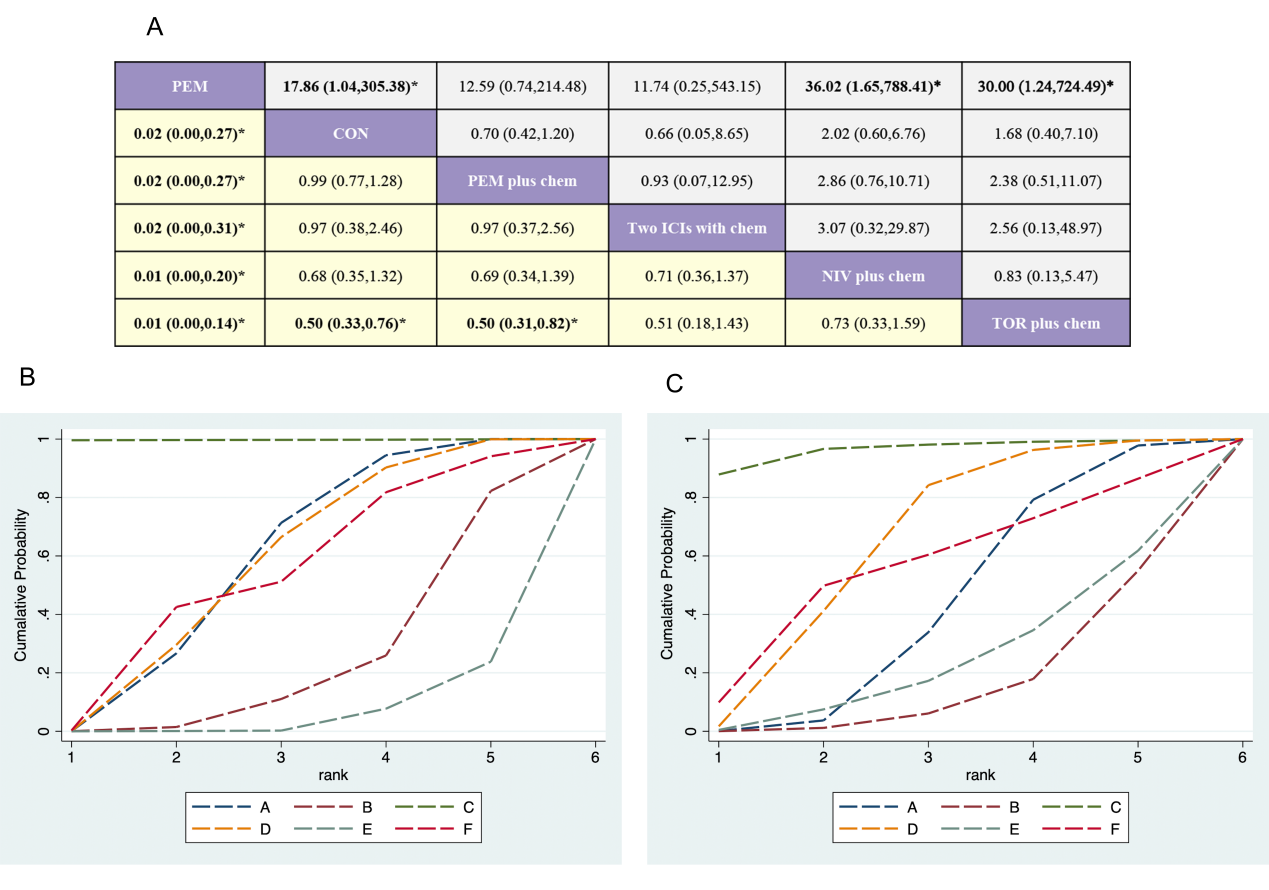


**Supplementary Figure 3.** Safety profile (A); ranking curves of grade 1-5 (B), ranking curves of grade 3-5 (C) according to the drug based network meta-analysis in thrombocytopenia. ICI=immune checkpoint inhibitor.

* High certainty of evidence.

Abbreviations in Fig S3B: A = chemotherapy with/without placebo; B = nivolumab plus chemotherapy; C = pembrolizumab; D = pembrolizumab plus chemotherapy; E = toripalimab plus chemotherapy; F = two ICI drugs with chemotherapy.

Abbreviations in Fig S3C: A = chemotherapy with/without placebo; B = nivolumab plus chemotherapy; C= pembrolizumab; D= pembrolizumab plus chemotherapy; E = toripalimab plus chemotherapy; F = two ICI drugs with chemotherapy.


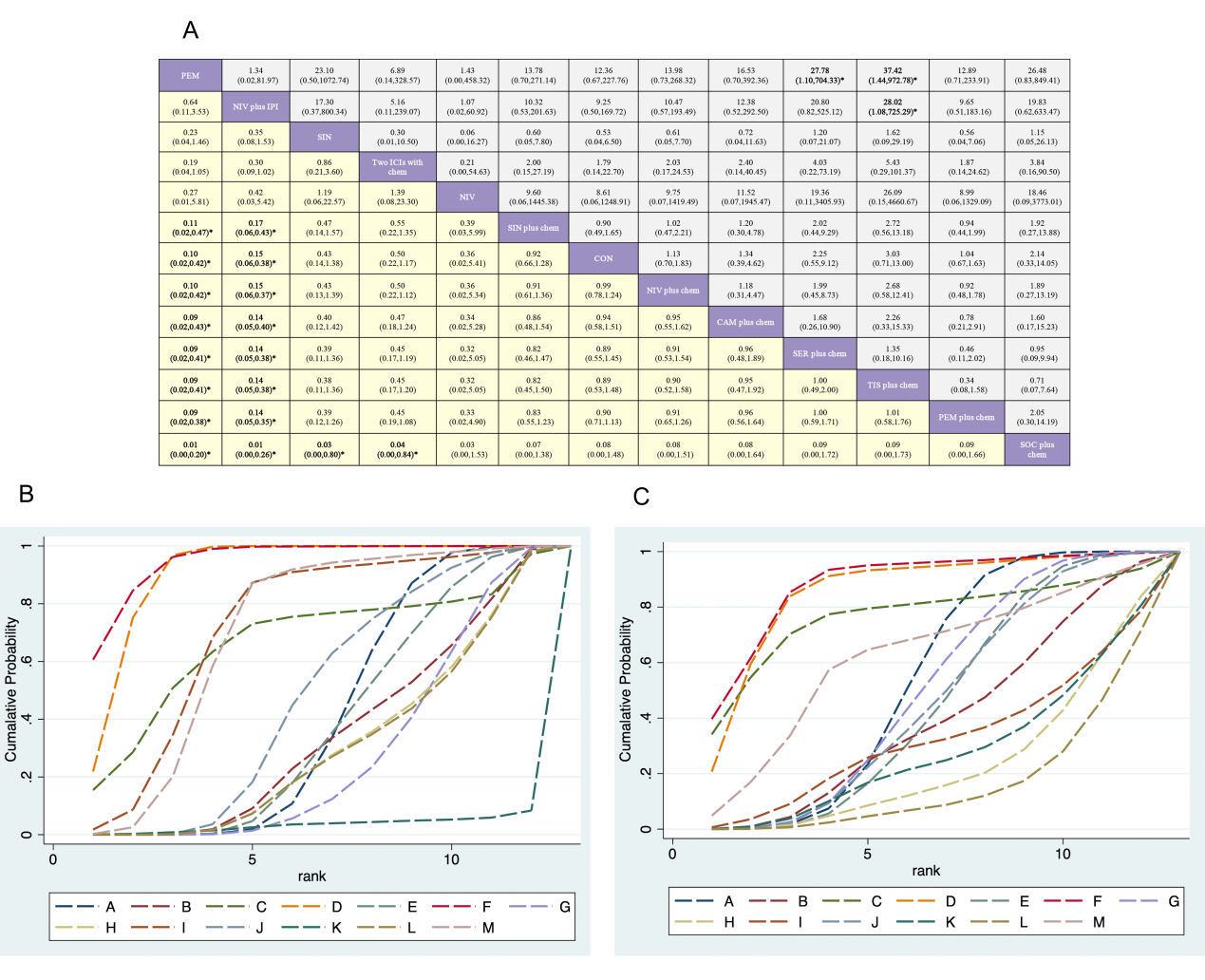


**Supplementary Figure 4.** Safety profile (A), ranking curves of grade 1-5 (B), ranking curves of grade 3-5 (C) according to the drug based network meta-analysis in platelet count decreased. ICI=immune checkpoint inhibitor.

* High certainty of evidence.

Abbreviations in Fig S4B: A = chemotherapy with/without placebo; B= camrelizumab plus chemotherapy; C = nivolumab; D = nivolumab plus ipilimumab; E = nivolumab plus chemotherapy; F = pembrolizumab; G = pembrolizumab plus chemotherapy; H =serplulimab plus chemotherapy; I = sintilimab; J = sintilimab plus chemotherapy; K = socazolimab plus chemotherapy; L = tislelizumab plus chemotherapy; M = two ICI drugs with chemotherapy.

Abbreviations in Fig S4B: A = chemotherapy with/without placebo; B= camrelizumab plus chemotherapy; C = nivolumab; D = nivolumab plus ipilimumab; E = nivolumab plus chemotherapy; F = pembrolizumab; G = pembrolizumab plus chemotherapy; H =serplulimab plus chemotherapy; I = sintilimab; J = sintilimab plus chemotherapy; K = socazolimab plus chemotherapy; L = tislelizumab plus chemotherapy; M = two ICI drugs with chemotherapy.


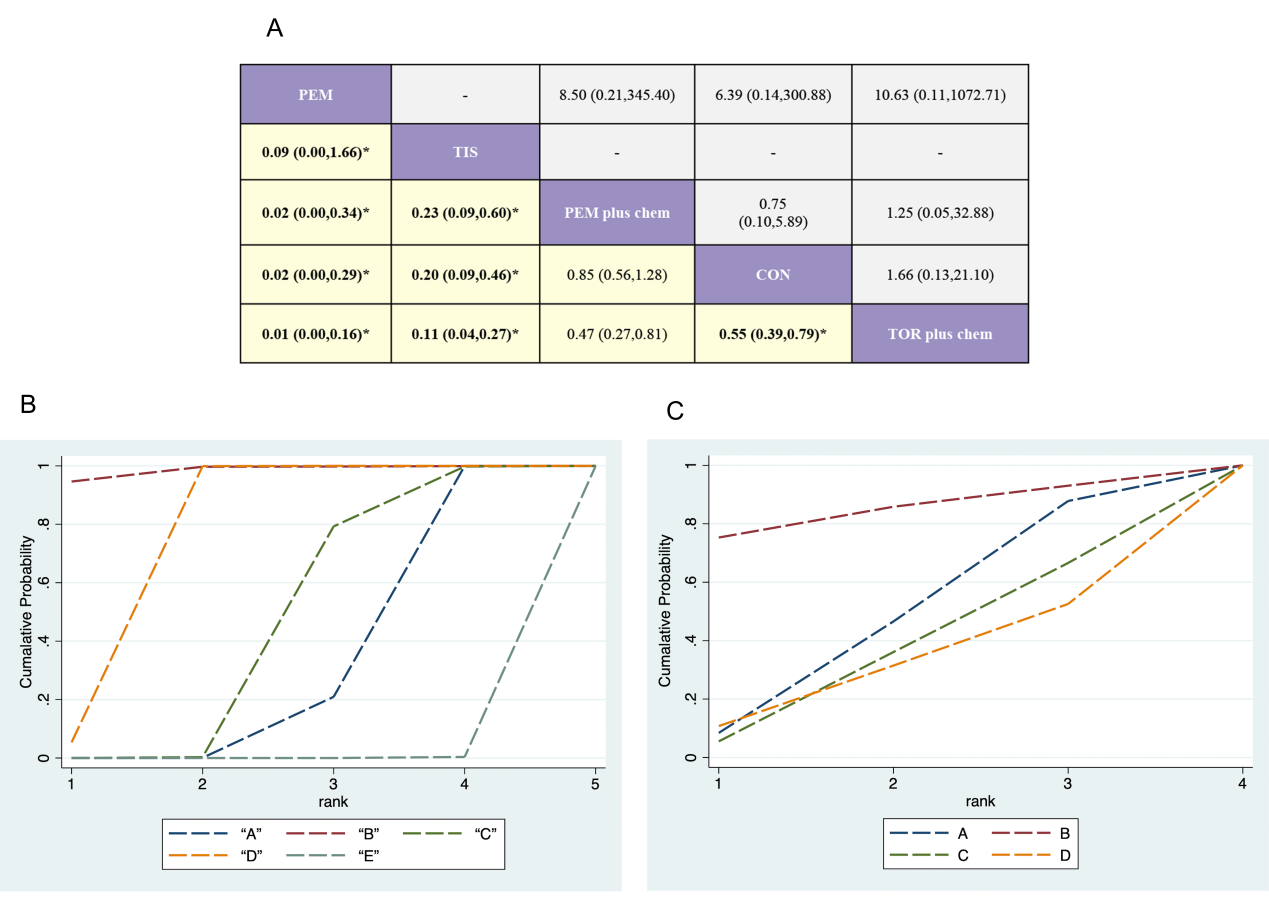


**Supplementary Figure 5.** Safety profile (A), ranking curves of grade 1-5 (B), ranking curves of grade 3-5 (C) according to the drug based network meta-analysis in leukopenia.

* High certainty of evidence.

Abbreviations in Fig S5B: A = chemotherapy with/without placebo; B = pembrolizumab; C = pembrolizumab plus chemotherapy; D = tislelizumab; E = toripalimab plus chemotherapy.

Abbreviations in Fig S5C: A = chemotherapy with/without placebo; B = pembrolizumab; C = pembrolizumab plus chemotherapy; D = toripalimab plus chemotherapy.


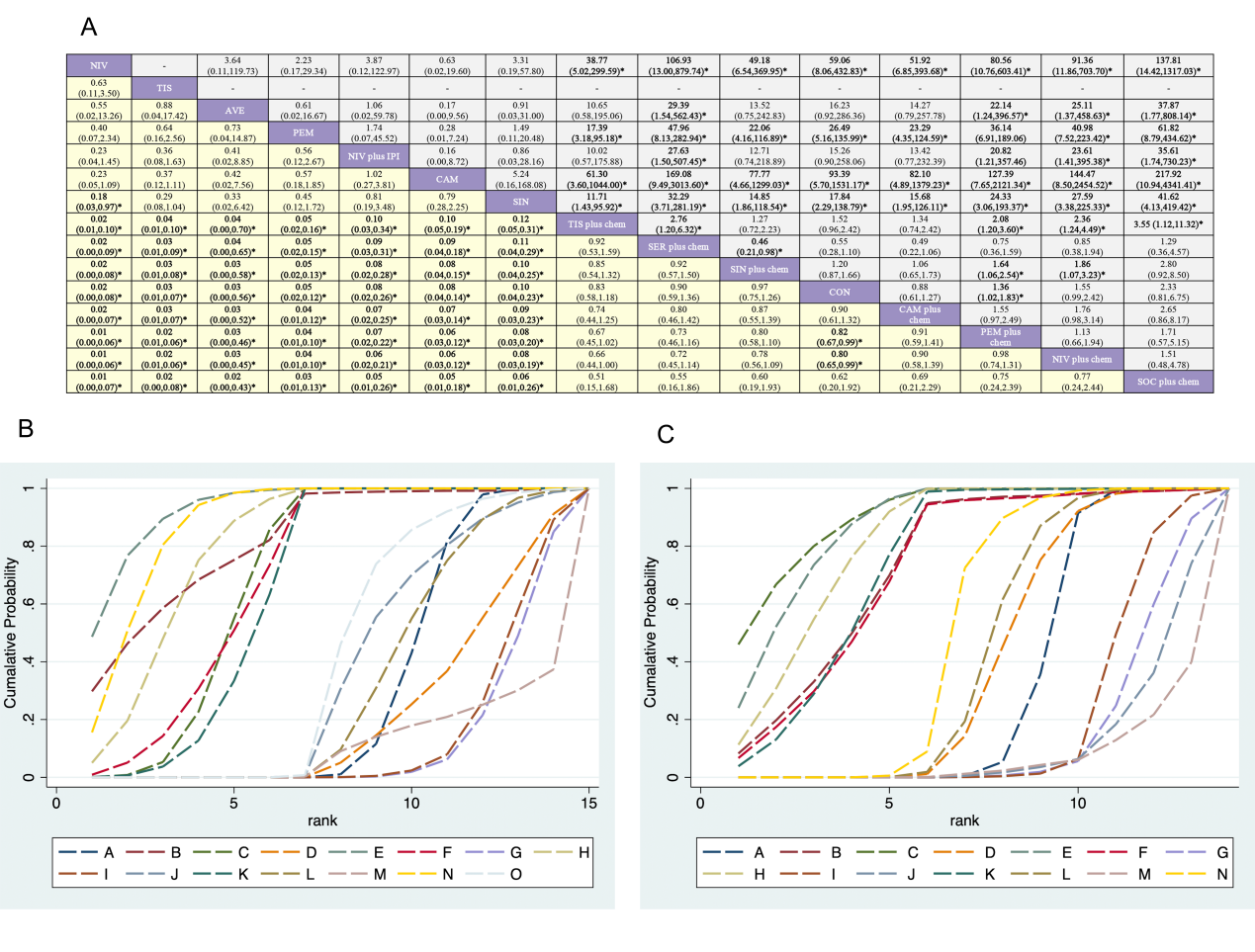


**Supplementary Figure 6.** Safety profile (A), ranking curves of grade 1-5 (B), ranking curves of grade 3-5 (C) according to the drug based network meta-analysis in white blood cell count decreased.

* High certainty of evidence.

Abbreviations in Fig S6B: A = chemotherapy with/without placebo; B = avelumab; C= camrelizumab; D= camrelizumab plus chemotherapy; E = nivolumab; F = nivolumab plus ipilimumab; G = nivolumab plus chemotherapy; H = pembrolizumab; I = pembrolizumab plus chemotherapy; J =serplulimab plus chemotherapy; K = sintilimab; L = sintilimab plus chemotherapy; M = socazolimab plus chemotherapy; N = tislelizumab; O = tislelizumab plus chemotherapy.

Abbreviations in Fig S6C: A = chemotherapy with/without placebo; B = avelumab; C= camrelizumab; D= camrelizumab plus chemotherapy; E = nivolumab; F = nivolumab plus ipilimumab; G = nivolumab plus chemotherapy; H = pembrolizumab; I = pembrolizumab plus chemotherapy; J =serplulimab plus chemotherapy; K = sintilimab; L = sintilimab plus chemotherapy; M = socazolimab plus chemotherapy; N = tislelizumab plus chemotherapy.


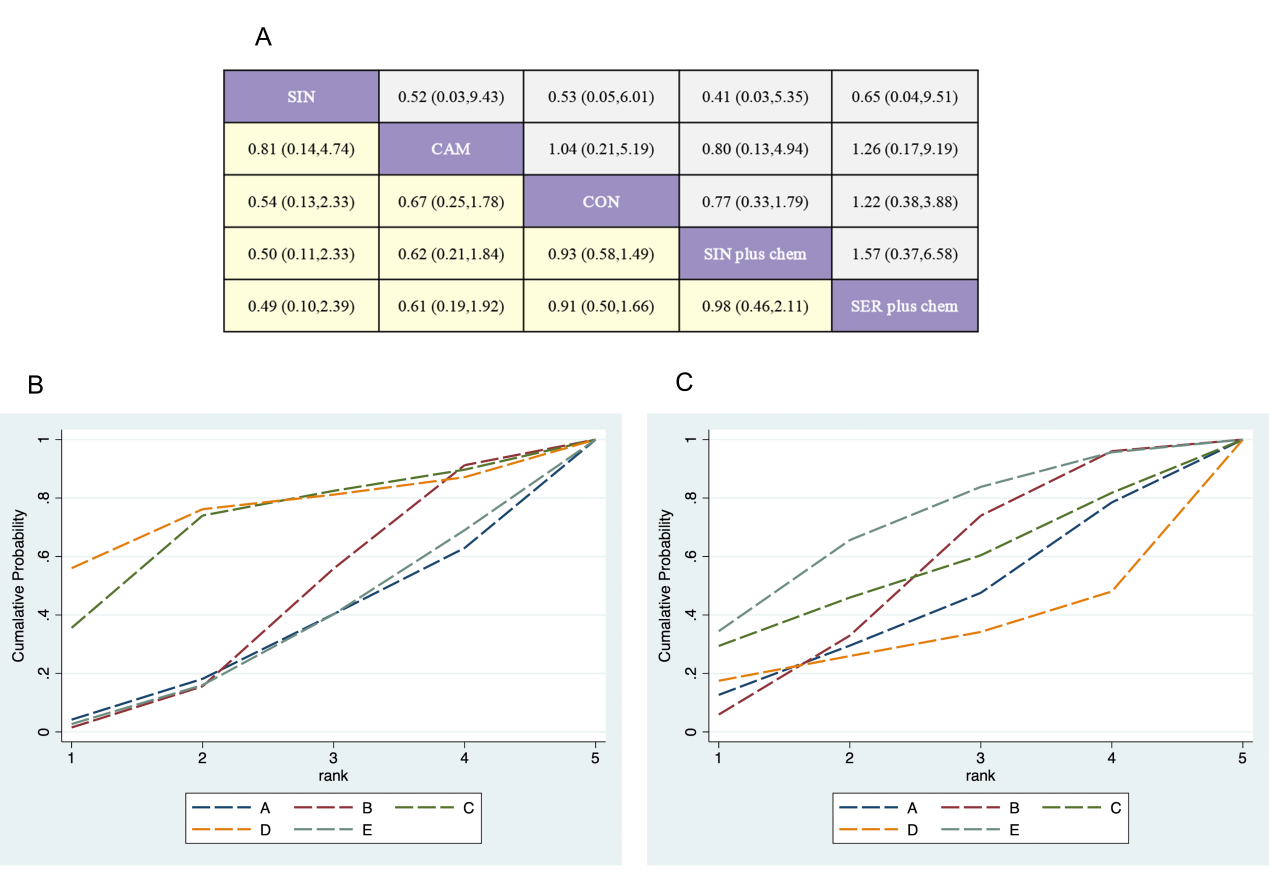


**Supplementary Figure 7.** Safety profile (A), ranking curves of grade 1-5 (B); ranking curves of grade 3-5 (C) according to the drug based network meta-analysis in lymphocyte count decreased.

* High certainty of evidence.

Abbreviations in Fig S7B: A = sintilimab; B = camrelizumab; C= chemotherapy with/without placebo; D= sintilimab plus chemotherapy; E = serplulimab plus chemotherapy.

Abbreviations in Fig S7C: A = sintilimab plus chemotherapy; B = camrelizumab; C= chemotherapy with/without placebo; D= serplulimab plus chemotherapy; E = sintilimab.


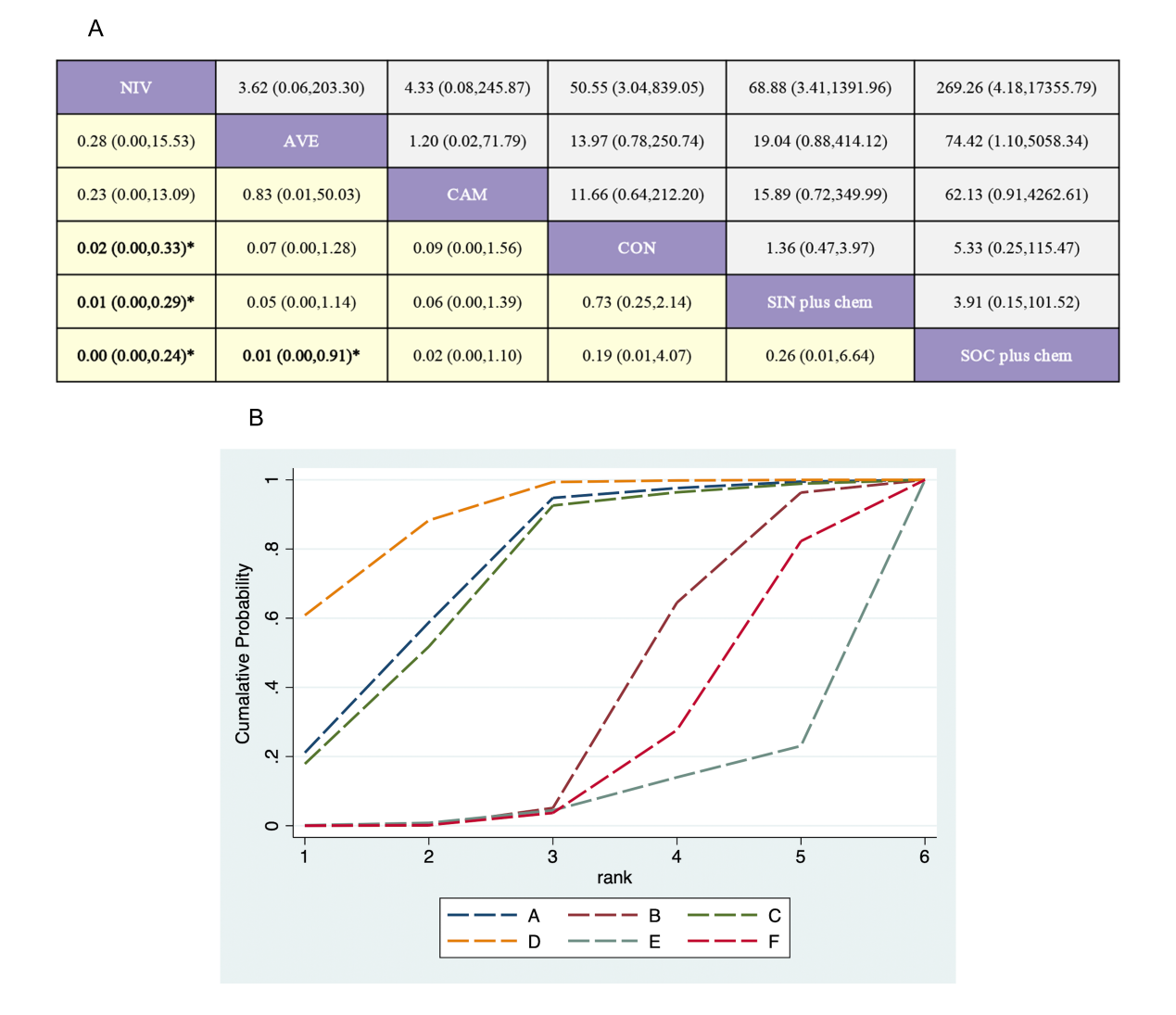


**Supplementary Figure 8.** Safety profile (A); ranking curves of grade 3-5 (B) according to the drug based network meta-analysis in FN.

* High certainty of evidence.

Abbreviations in Fig S8B: A = nivolumab; B = avelumab; C= camrelizumab; D= chemotherapy with/without placebo; E = sintilimab plus chemotherapy; F = socazolimab plus chemotherapy.

Abbreviations in Fig S8C: A = nivolumab; B = avelumab; C= camrelizumab; D= chemotherapy with/without placebo; E = sintilimab plus chemotherapy; F = socazolimab plus chemotherapy.

**
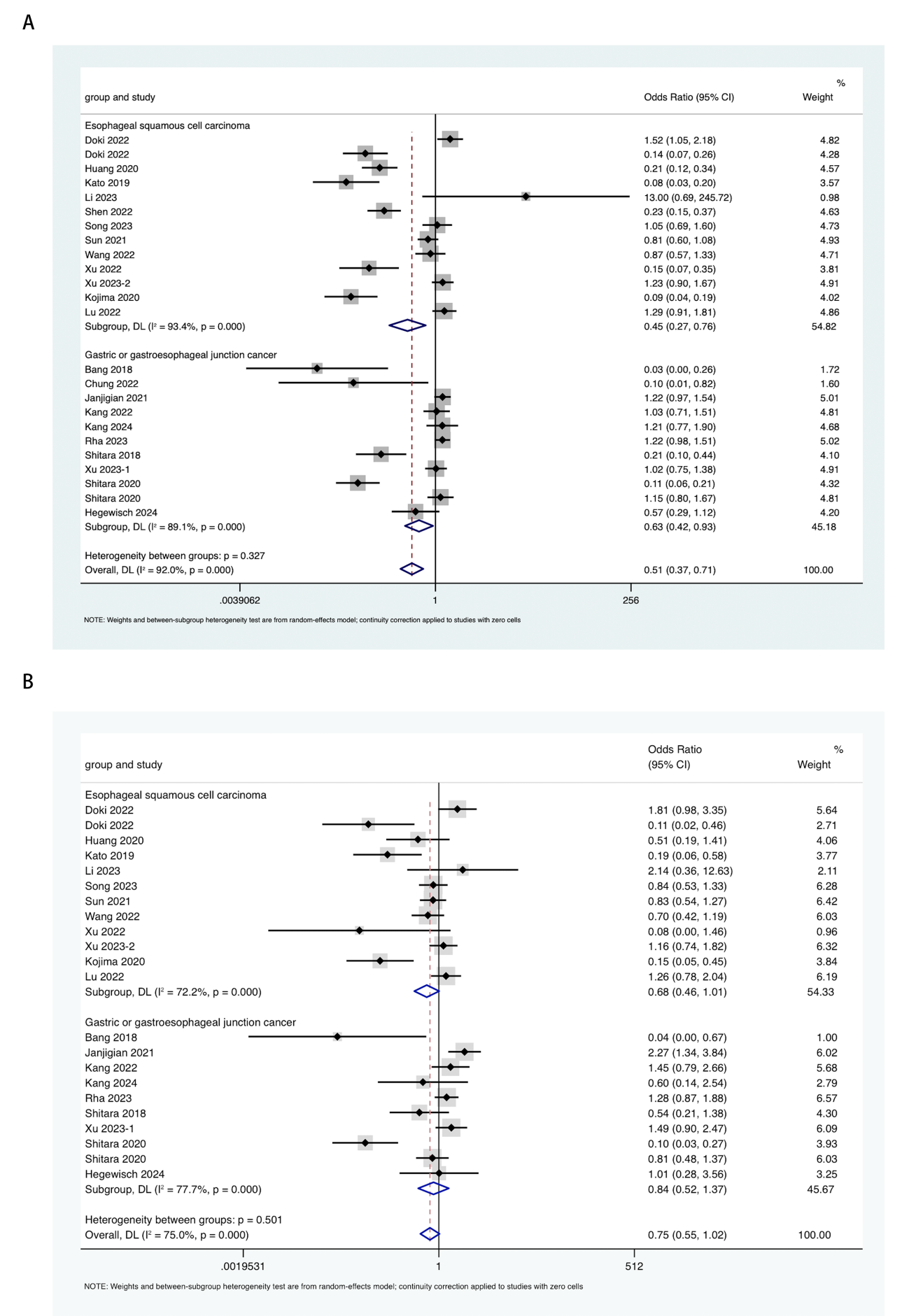
**

**Supplementary Figure 9. Subgroup analysis based on tumor type (Esophageal cancer versus Gastric or gastro-oesophageal junction cancer).** (A) Grade 1-5; (B) Grade 3-5.

**
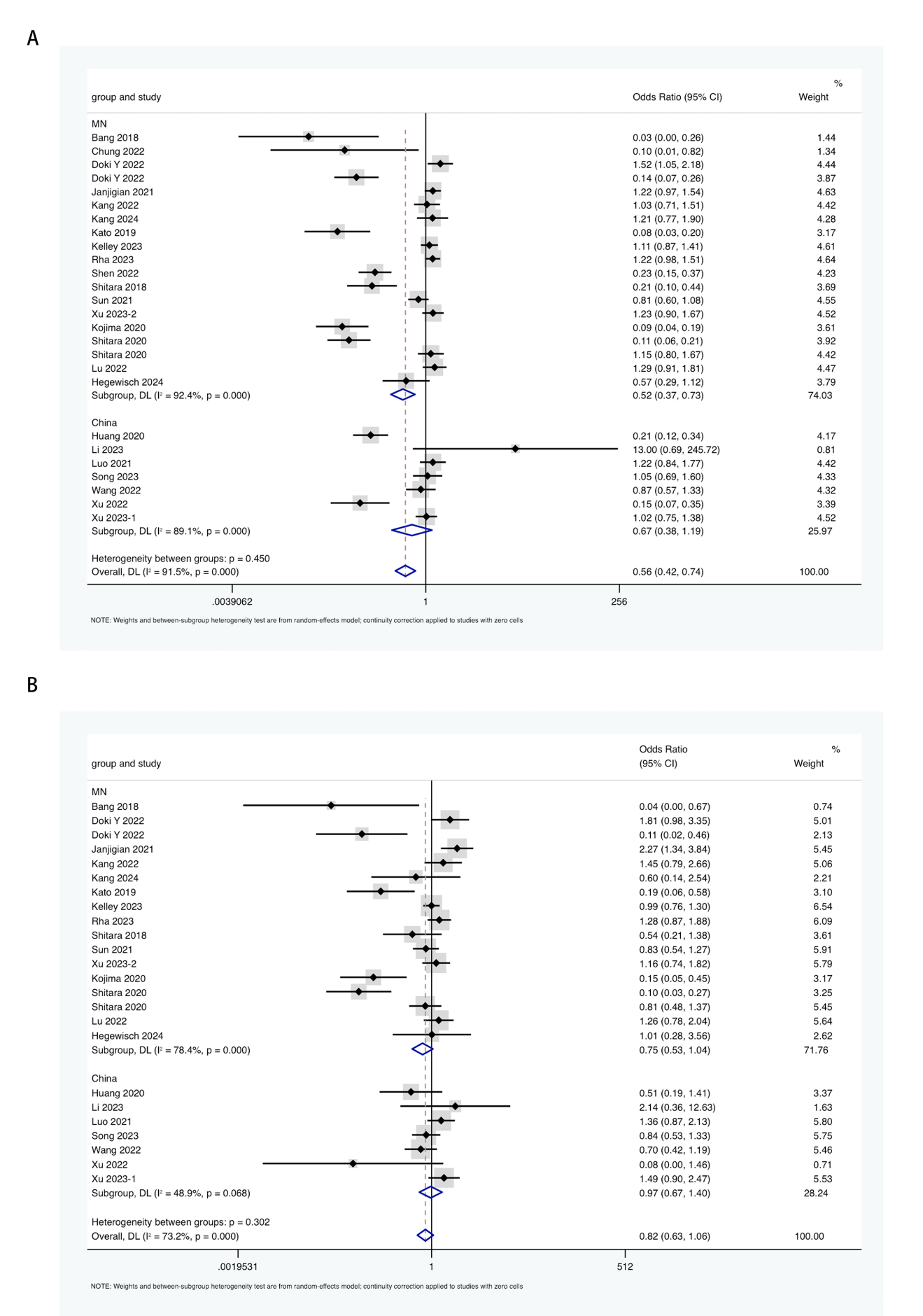
**

**Supplementary Figure 10. Subgroup analysis based on country category (MN versus China).** (A) Grade 1-5; (B) Grade 3-5.

**
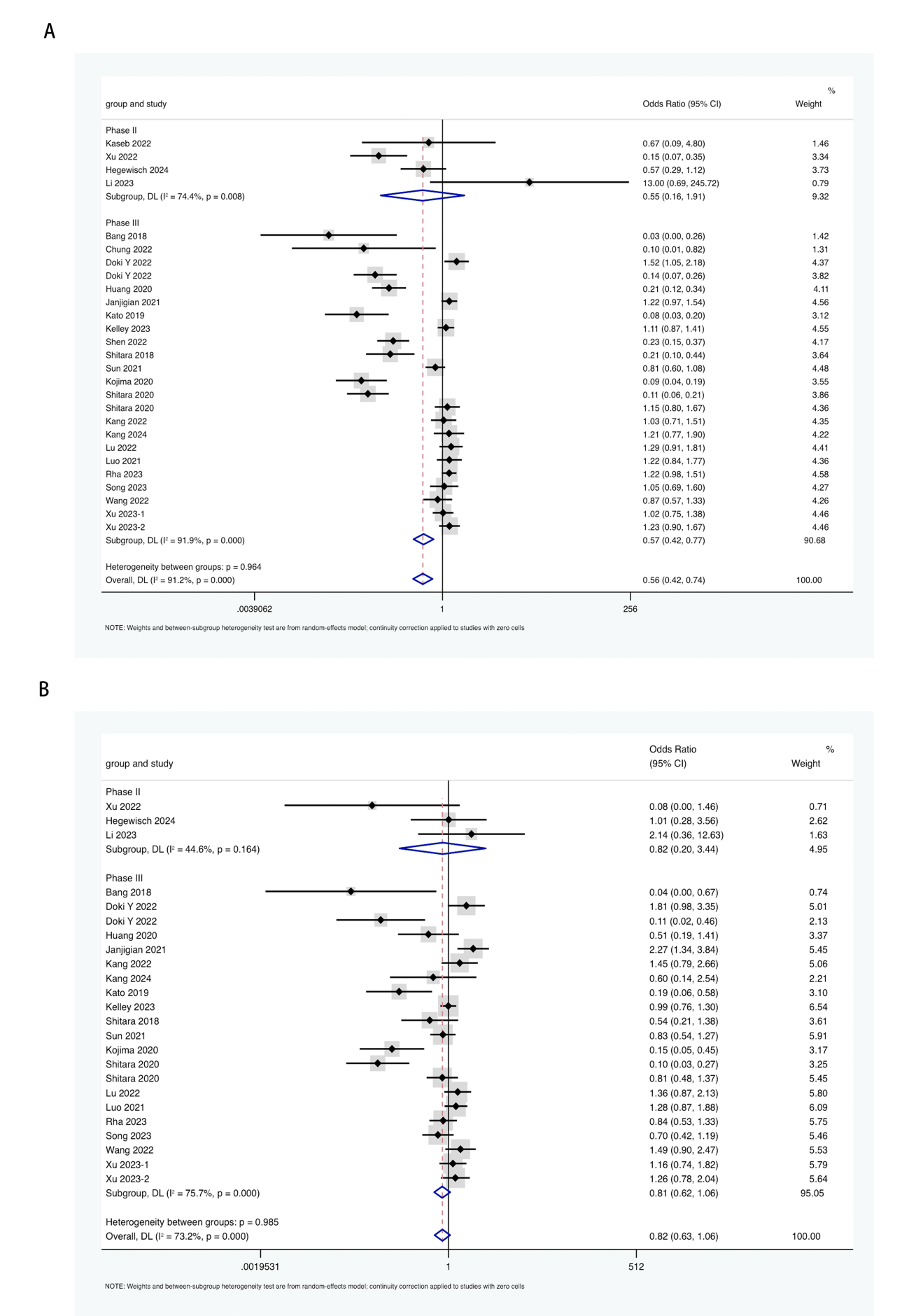
**

**Supplementary Figure 11. Subgroup analysis based on study phase (Phase II versus Phase III).** (A) Grade 1-5; (B) Grade 3-5.

**
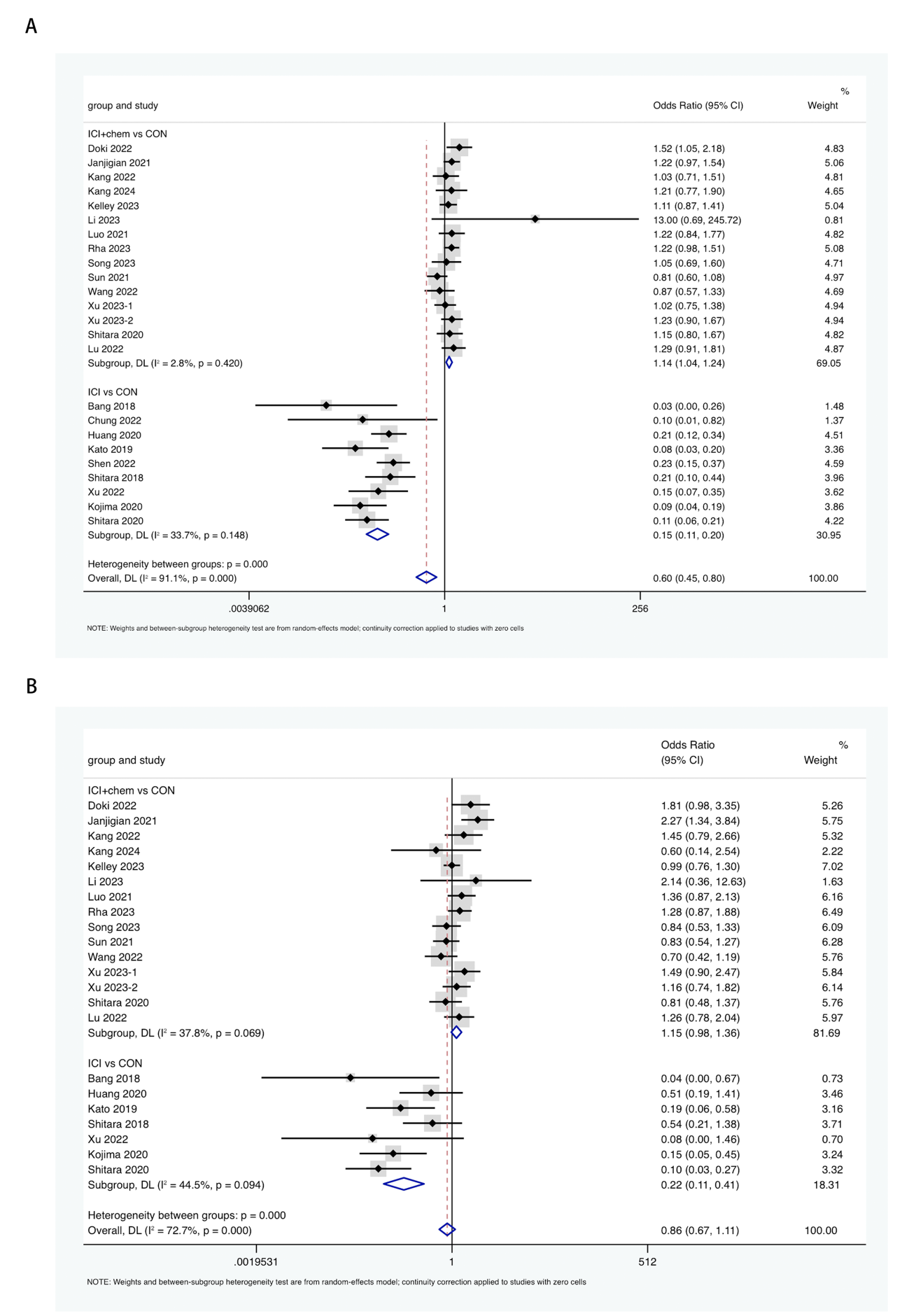
**

**Supplementary Figure 12. Subgroup analysis based on ICI regimen (ICI plus chemotherapy versus ICI).** (A) Grade 1-5; (B) Grade 3-5.

**
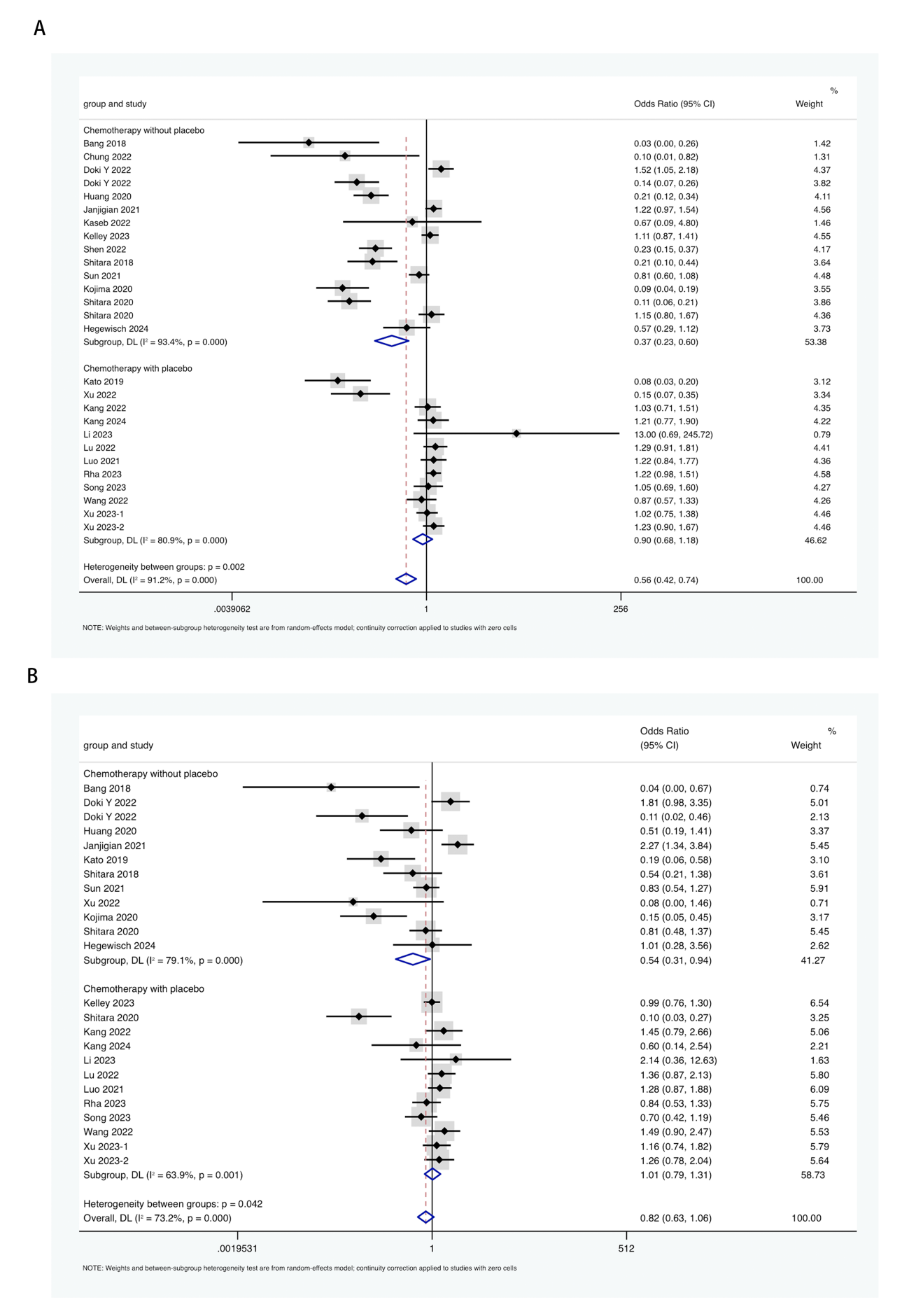
**

**Supplementary Figure 13. Subgroup analysis based on control group (chemotherapy without placebo versus chemotherapy with placebo).** (A) Grade 1-5; (B) Grade 3-5.

**
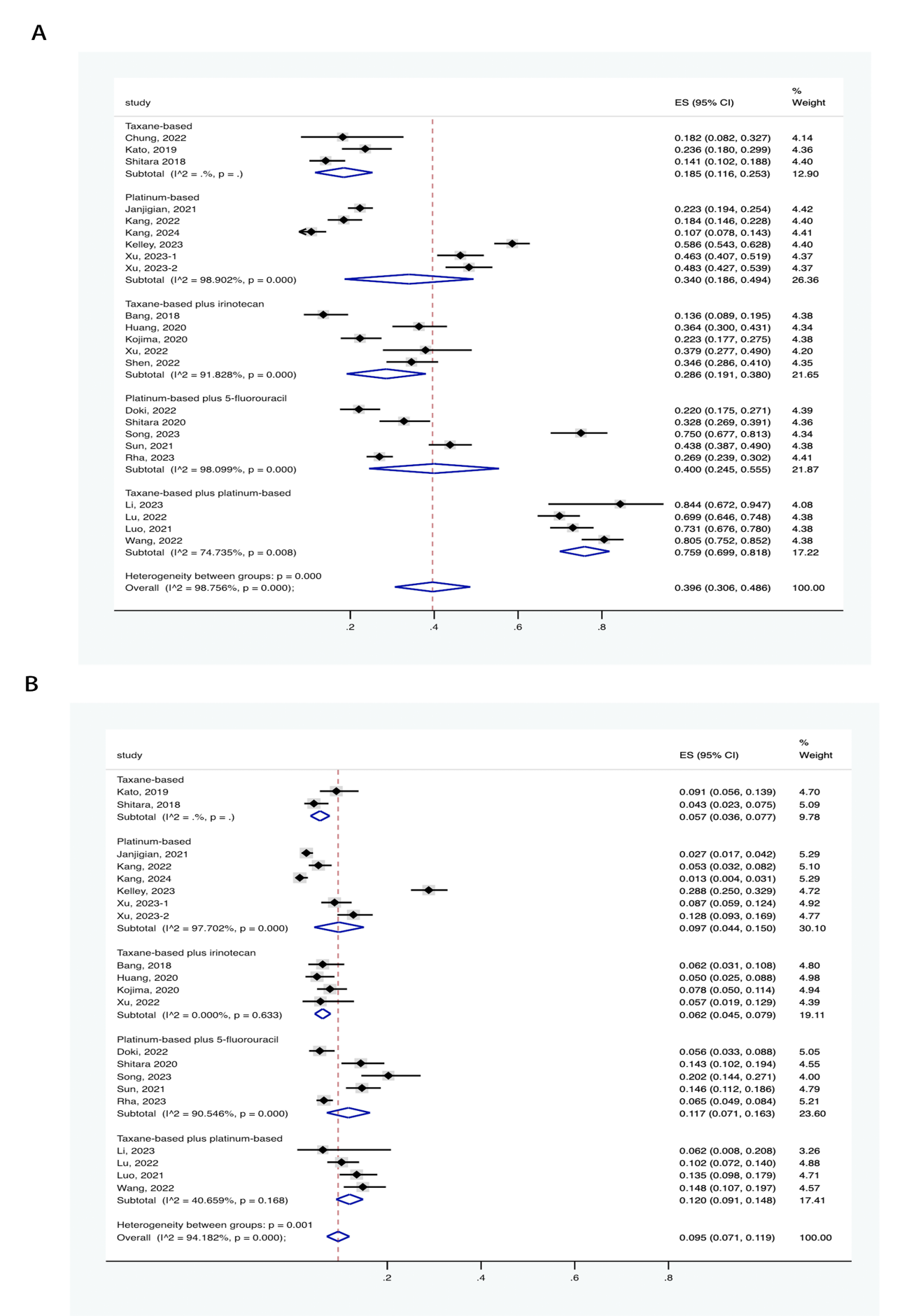
**

**Supplementary Figure 14. Subgroup analysis based on chemotherapy regimen (taxane-based versus platinum-based versus taxane-based plus irinotecan versus platinum-based plus 5-fluorouracil versus taxane-based plus platinum-based).** (A) Grade 1-5; (B) Grade 3-5.

**
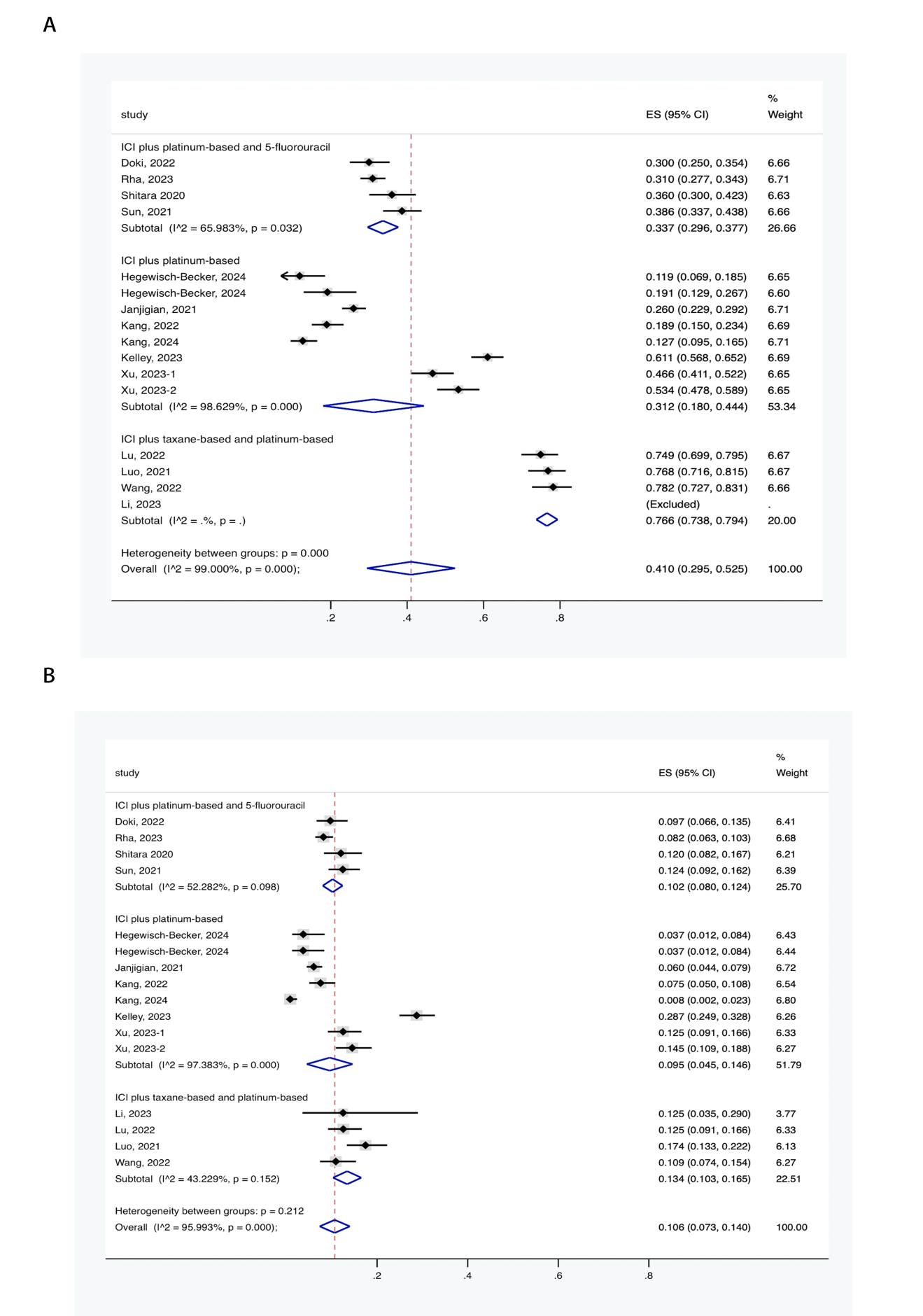
**

**Supplementary Figure 15. Subgroup analysis based on ICI plus different chemotherapy regimens (ICI plus platinum-based, ICI plus platinum- based and 5-fluorouracil, ICI plus taxane-based and platinum-based).** (A) Grade 1-5; (B) Grade 3-5.


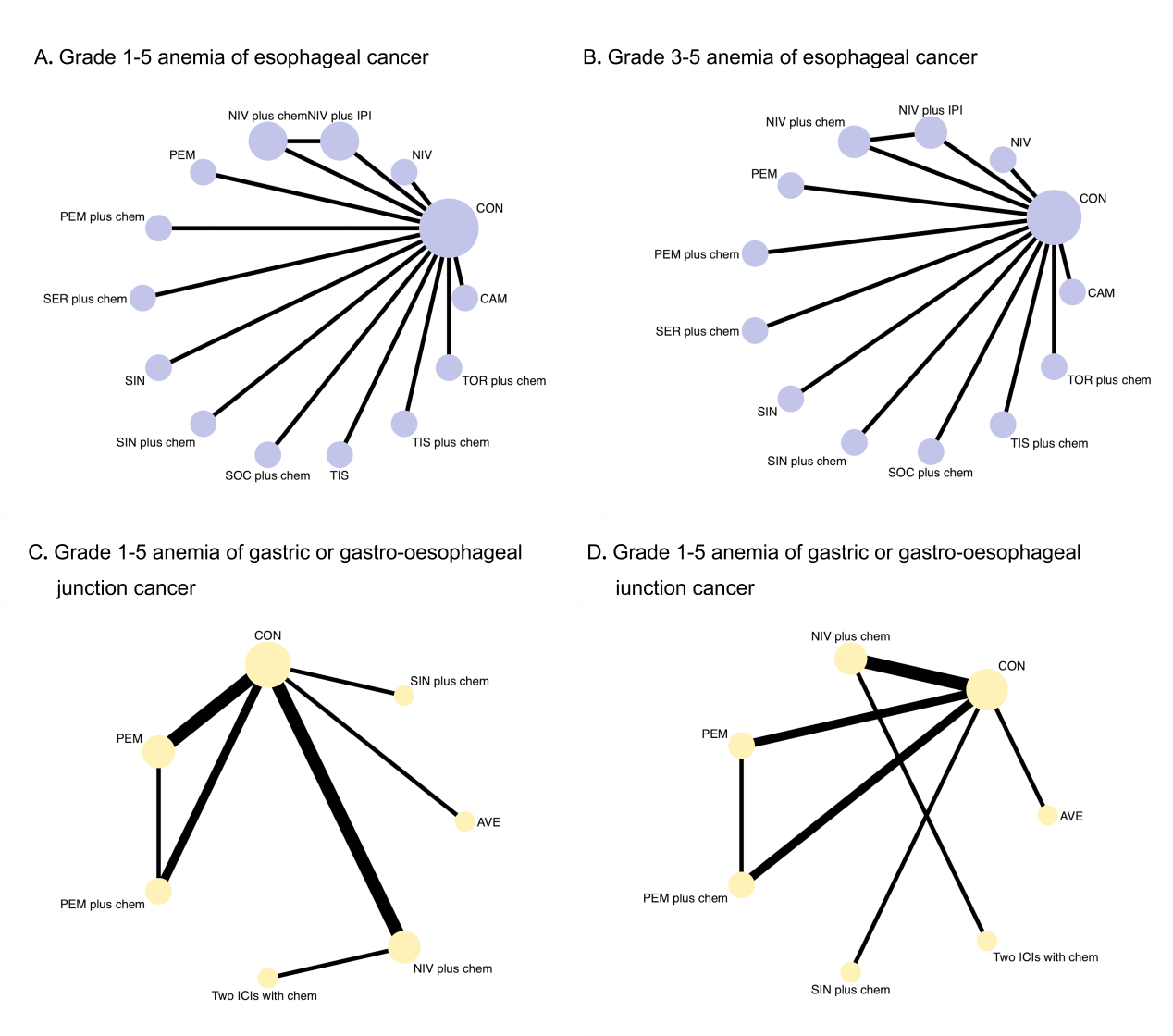


**Supplementary Figure 16.** Network plots of eligible direct comparisons (Subgroup analysis based on cancer type)

Each circular node represents a type of treatment. The circle size is proportional to the total number of patients. The width of lines is proportional to the number of studies performing head-to-head comparisons in the same study.

ICI = immune checkpoint inhibitor; AVE = avelumab; CAM = camrelizumab; CAM plus chem = camrelizumab plus chemotherapy; CON = chemotherapy with/without placebo; NIV = nivolumab; NIV plus IPI = nivolumab plus ipilimumab; NIV plus chem = nivolumab plus chemotherapy; PEM = pembrolizumab; PEM plus chem = pembrolizumab plus chemotherapy; SER plus chem = serplulimab plus chemotherapy; SIN = sintilimab; SIN plus chem = sintilimab plus chemotherapy; SOC plus chem = socazolimab plus chemotherapy; TIS = tislelizumab; TIS plus chem = tislelizumab plus chemotherapy; TOR plus chem = toripalimab plus chemotherapy; Two ICIs with chem = two ICI drugs with chemotherapy.


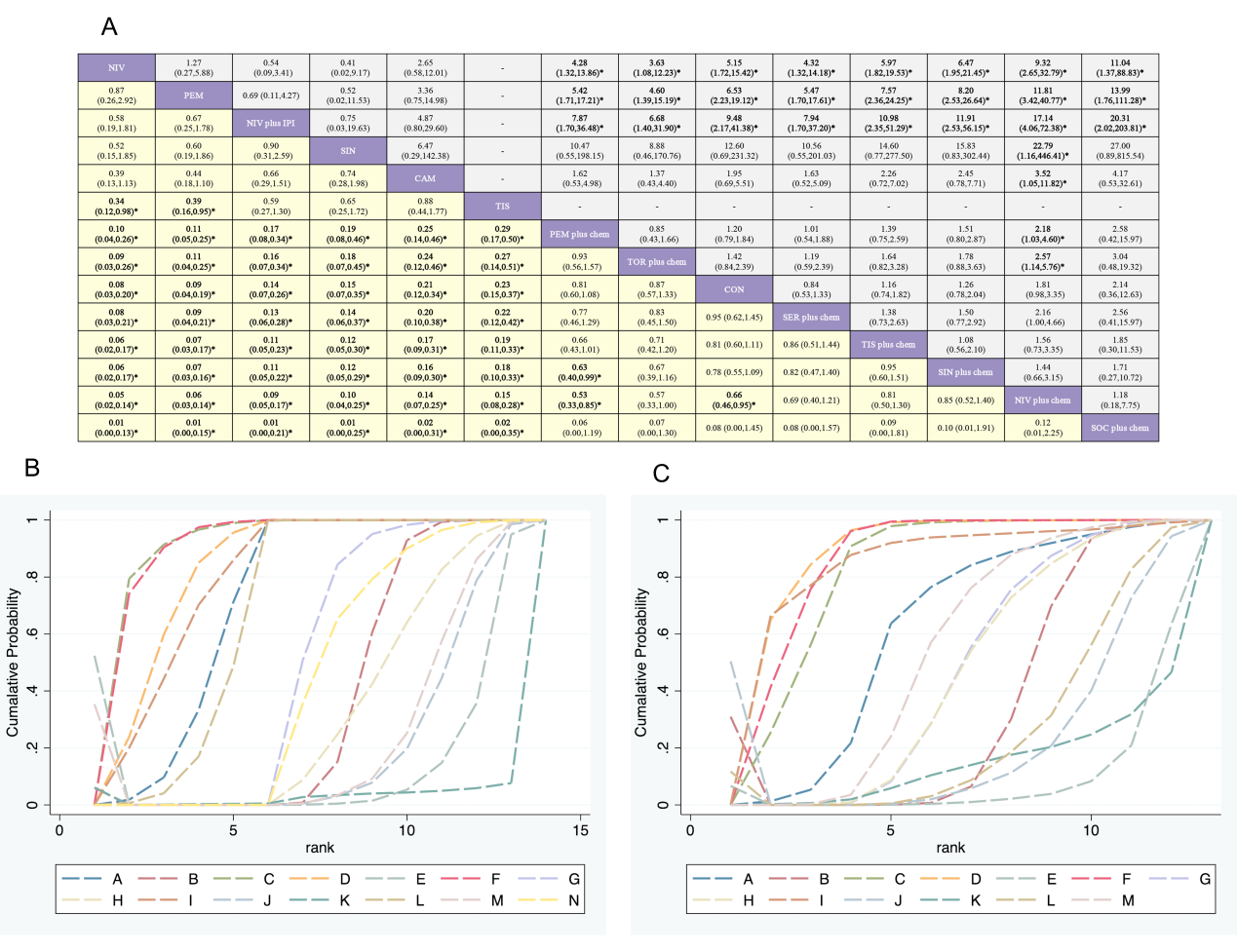


**Supplementary Figure 17.** Safety profile (A), ranking curves of grade 1-5 anemia (B), ranking curves of grade 3-5 anemia (C) according to the drug based network meta-analysis in esophageal cancer.

* High certainty of evidence.

Abbreviations in Fig S9B: A = nivolumab plus chemotherapy; B = nivolumab plus ipilimumab; C= chemotherapy with/without placebo; D= camrelizumab; E = nivolumab; F = socazolimab plus chemotherapy; G = tislelizumab; H = serplulimab plus chemotherapy; I = pembrolizumab plus chemotherapy; J = toripalimab plus chemotherapy; K = sintilimab; L = tislelizumab plus chemotherapy; M = pembrolizumab; N = sintilimab plus chemotherapy.

Abbreviations in Fig S9C: A = nivolumab plus chemotherapy; B = nivolumab plus ipilimumab; C= chemotherapy with/without placebo; D= camrelizumab; E = nivolumab; F = socazolimab plus chemotherapy; G = serplulimab plus chemotherapy; H = pembrolizumab plus chemotherapy; I = toripalimab plus chemotherapy; J = sintilimab; K = tislelizumab plus chemotherapy; L = pembrolizumab; M = sintilimab plus chemotherapy.


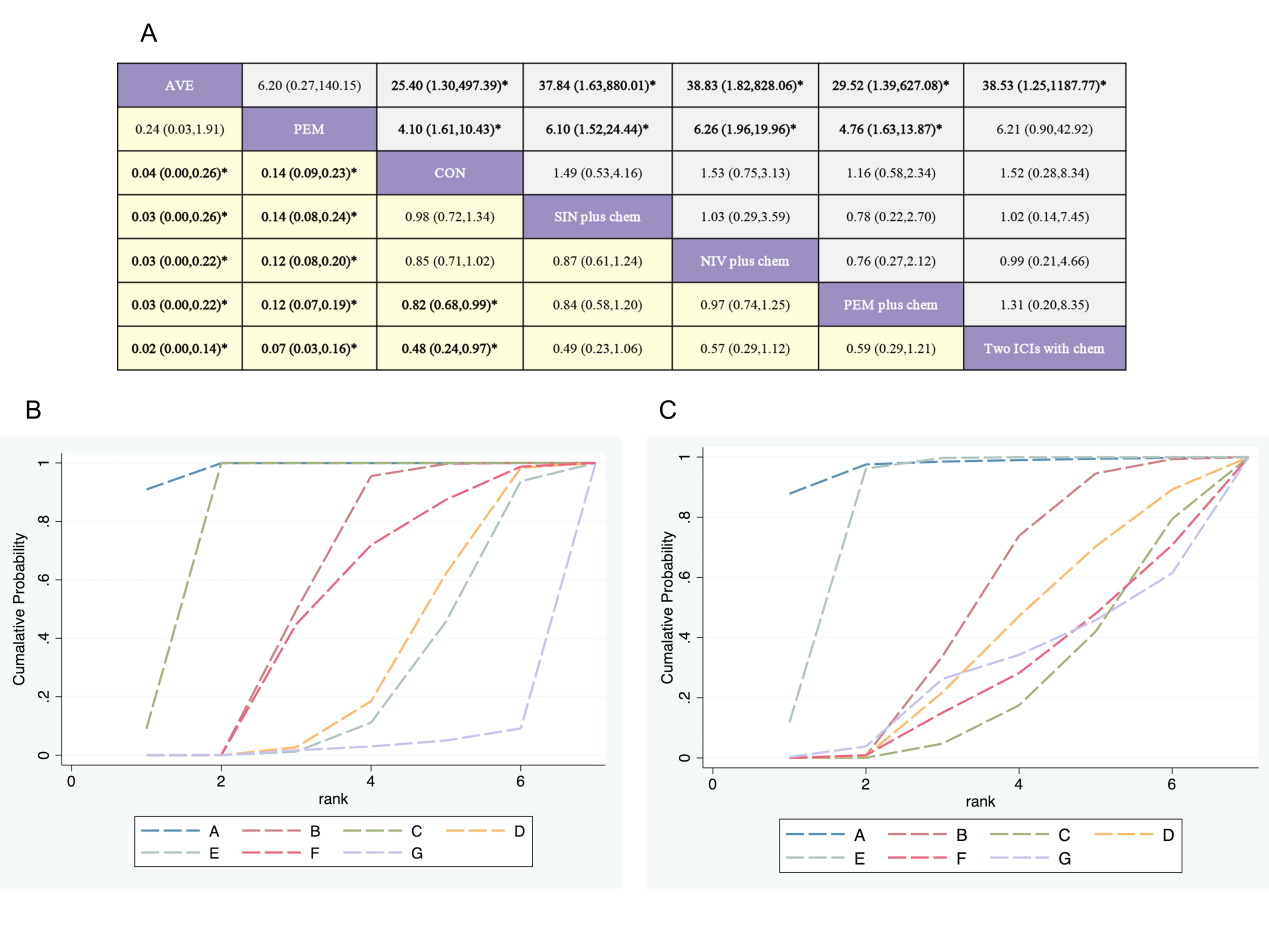


**Supplementary Figure 18.** Safety profile (A), ranking curves of grade 1-5 anemia (B), ranking curves of grade 3-5 anemia (C) according to the drug based network meta-analysis in gastric or gastro-oesophageal junction cancer. ICI=immune checkpoint inhibitor.

* High certainty of evidence.

Abbreviations in Fig S10B: A = avelumab; B = chemotherapy with/without placebo; C= pembrolizumab; D = nivolumab plus ipilimumab; E = pembrolizumab plus chemotherapy; F = sintilimab plus chemotherapy; G = two ICI drugs with chemotherapy.

Abbreviations in Fig S10C: A = avelumab; B = chemotherapy with/without placebo; C= nivolumab plus ipilimumab; D = pembrolizumab plus chemotherapy; E = pembrolizumab; F = sintilimab plus chemotherapy; G = two ICI drugs with chemotherapy.

**
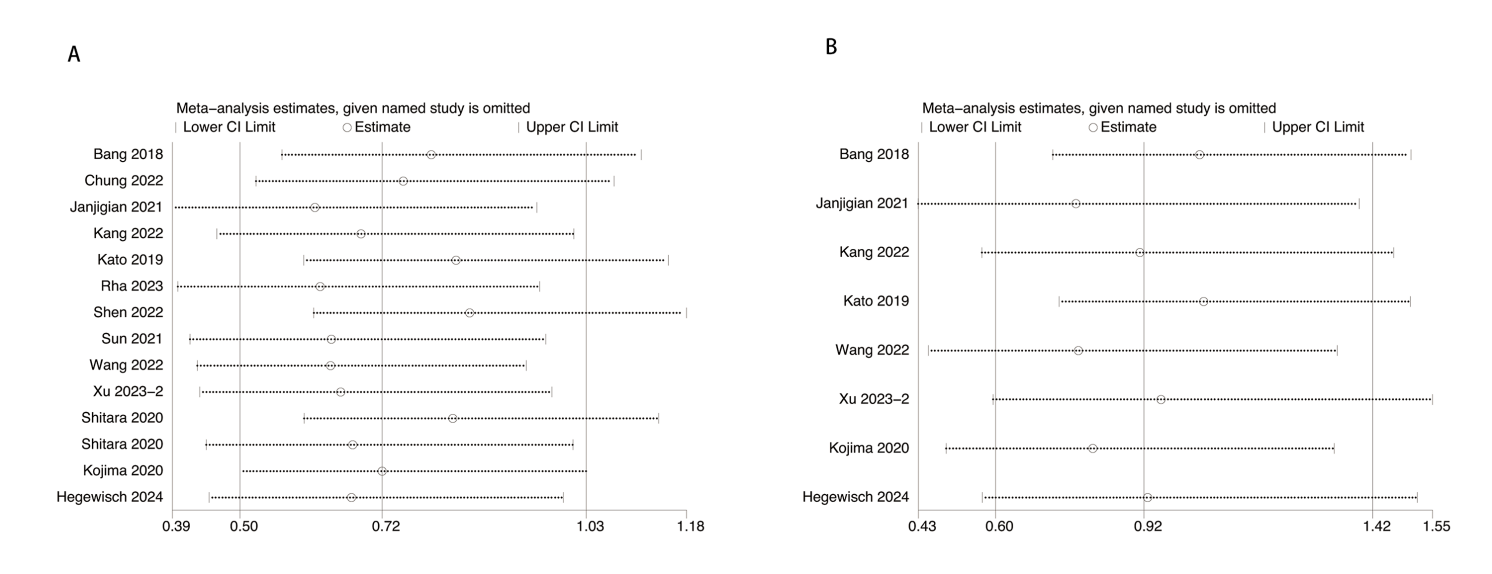
**

**Supplementary Figure 19. Sensitivity analysis: the influence of single study on the total merger effect.** (A) grade 1-5 neutropenia; (B) grade 3-5 neutropenia.

**
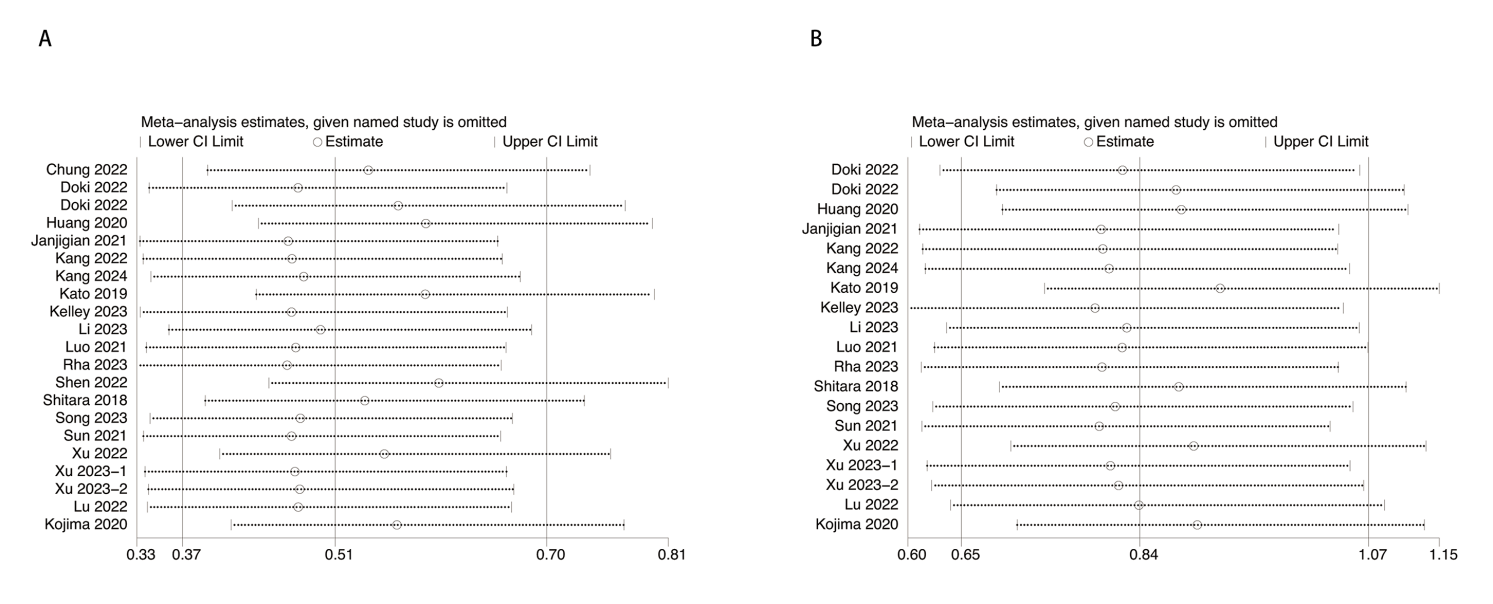
**

**Supplementary Figure 20. Sensitivity analysis: the influence of single study on the total merger effect.** (A) grade 1-5 neutrophil count decreased; (B) grade 3-5 neutrophil count decreased.

**
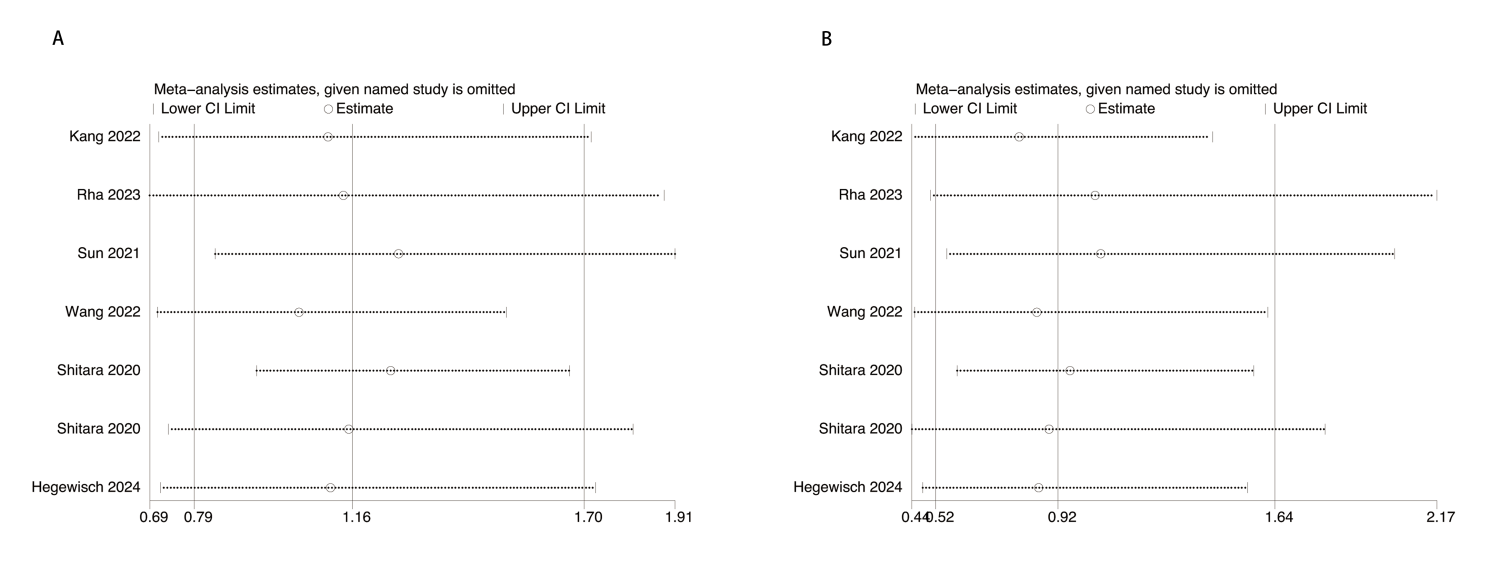
**

**Supplementary Figure 21. Sensitivity analysis: the influence of single study on the total merger effect.** (A) grade 1-5 thrombocytopenia; (B) grade 3-5 thrombocytopenia.

**
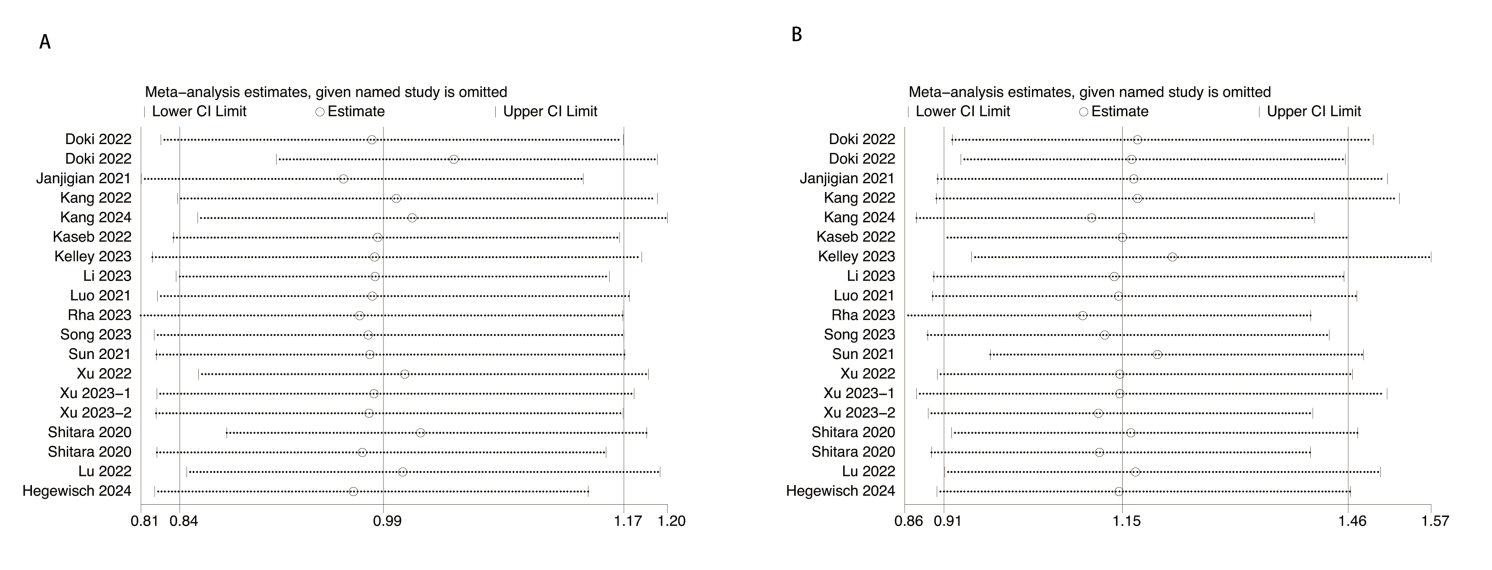
**

**Supplementary Figure 22. Sensitivity analysis: the influence of single study on the total merger effect.** (A) grade 1-5 platelet count decreased; (B) grade 3-5 platelet count decreased.

**
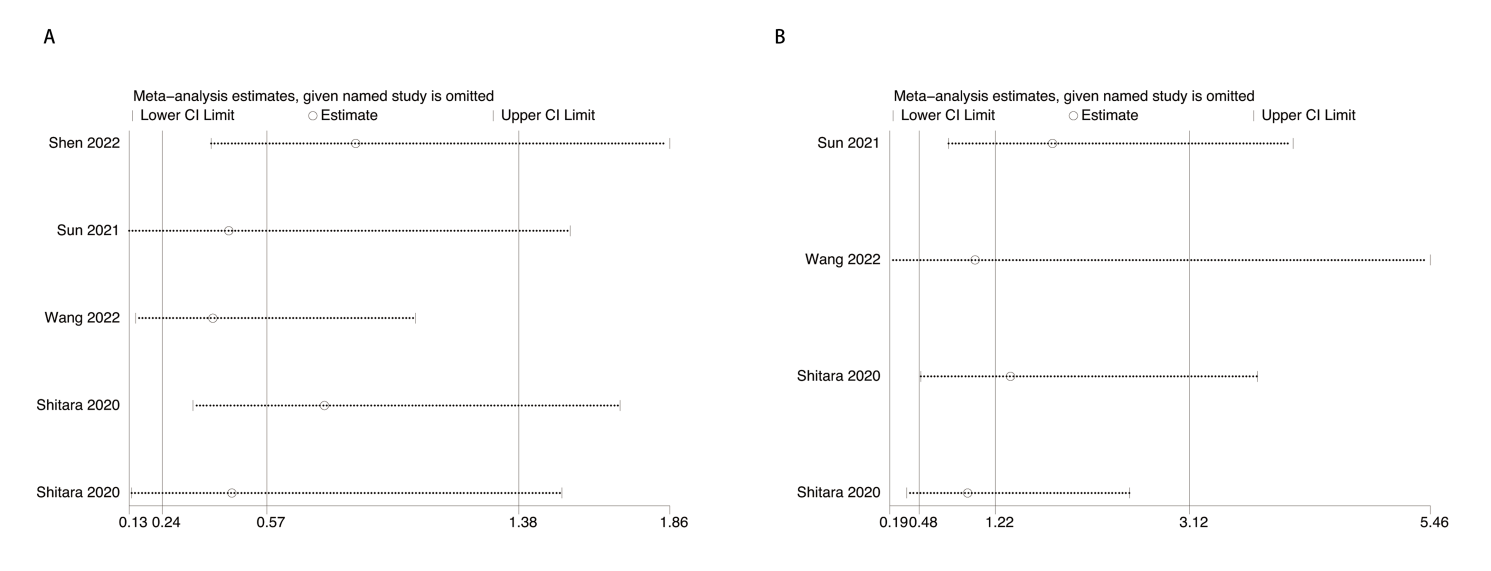
**

**Supplementary Figure 23. Sensitivity analysis: the influence of single study on the total merger effect.** (A) grade 1-5 leukopenia; (B) grade 3-5 leukopenia.

**
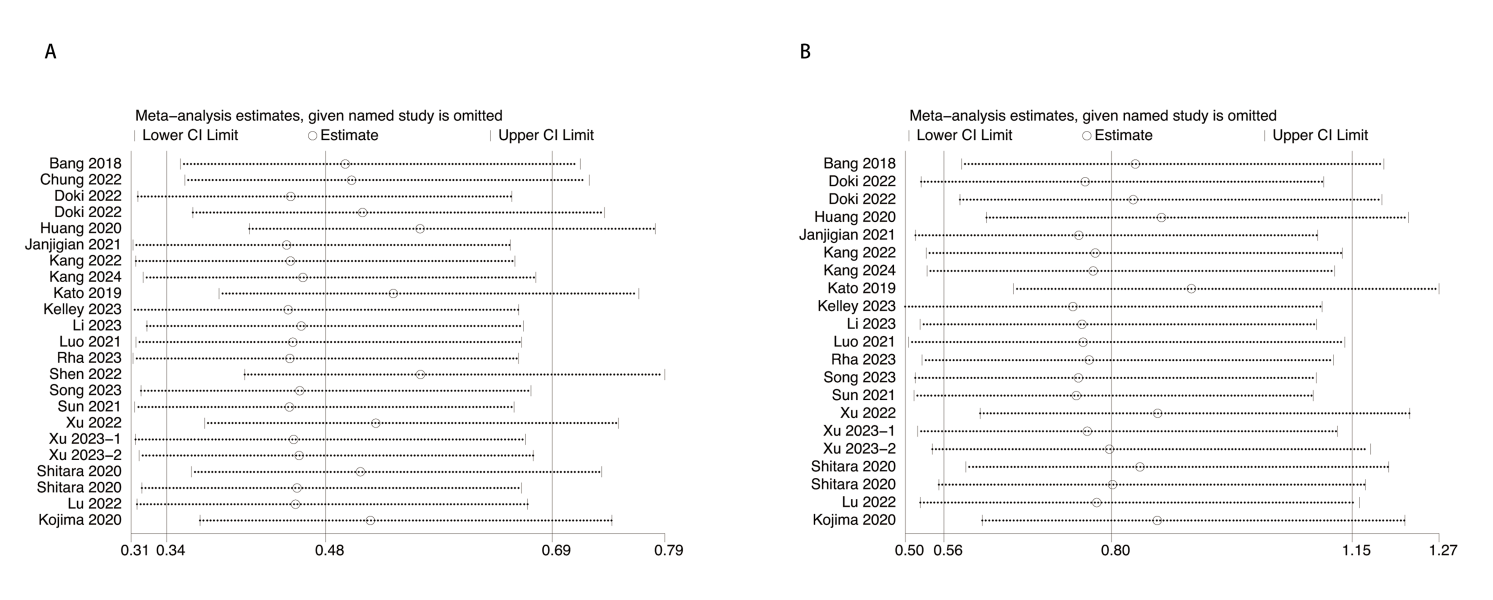
**

**Supplementary Figure 24. Sensitivity analysis: the influence of single study on the total merger effect.** (A) grade 1-5 WBC count decreased; (B) grade 3-5 WBC count decreased.

**
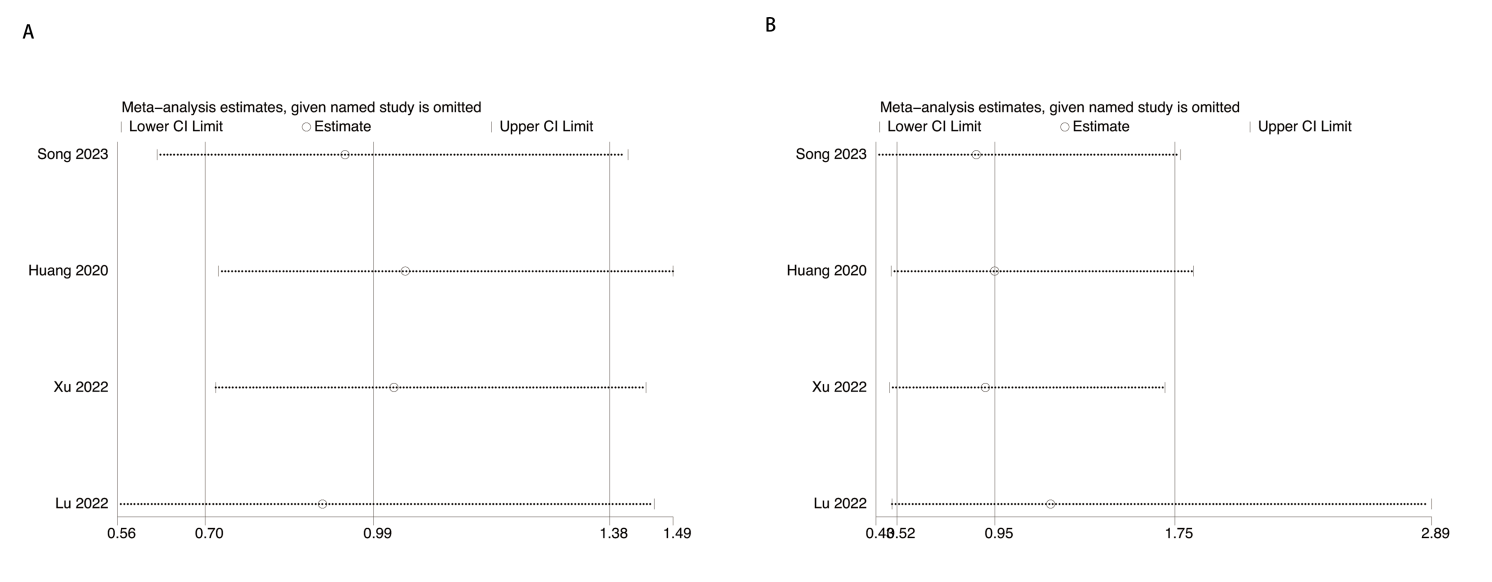
**

**Supplementary Figure 25. Sensitivity analysis: the influence of single study on the total merger effect.** (A) grade 1-5 lymphocyte count decreased; (B) grade 3-5 lymphocyte count decreased.

**
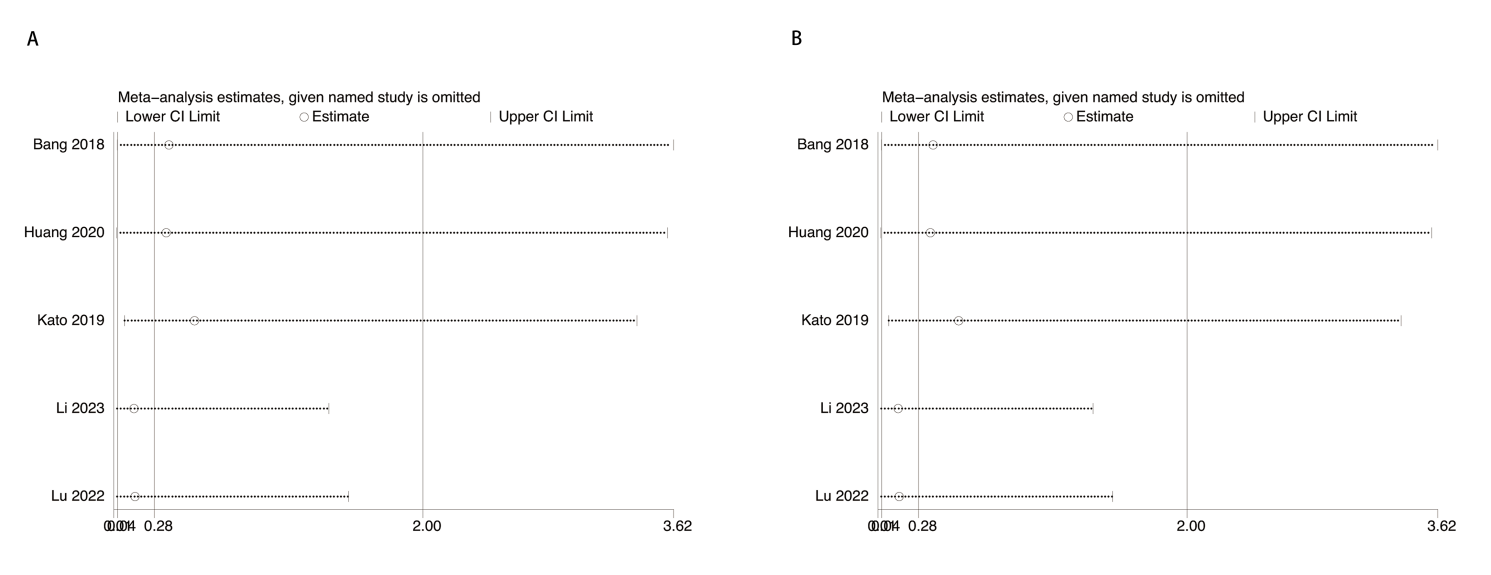
**

**Supplementary Figure 26. Sensitivity analysis: the influence of single study on the total merger effect.** (A) grade 1-5 FN; (B) grade 3-5 FN.
